# Chloride sensing by WNK1 regulates NLRP3 inflammasome activation and pyroptosis

**DOI:** 10.1038/s41467-021-24784-4

**Published:** 2021-07-27

**Authors:** Lindsey Mayes-Hopfinger, Aura Enache, Jian Xie, Chou-Long Huang, Robert Köchl, Victor L. J. Tybulewicz, Teresa Fernandes-Alnemri, Emad S. Alnemri

**Affiliations:** 1 Department of Biochemistry and Molecular Biology, Thomas Jefferson University, Philadelphia, PA USA; 2 Department of Medicine, Division of Nephrology, University of Iowa Carver College of Medicine, Iowa City, IA USA; 3 The Francis Crick Institute, London, UK; 4 Kings College London, London, UK; 5 Imperial College, London, UK; 6 Sidney Kimmel Cancer Center, Thomas Jefferson University, Philadelphia, PA USA

**Keywords:** Cell death, Inflammasome, Monocytes and macrophages

## Abstract

The NLRP3 inflammasome mediates the production of proinflammatory cytokines and initiates inflammatory cell death. Although NLRP3 is essential for innate immunity, aberrant NLRP3 inflammasome activation contributes to a wide variety of inflammatory diseases. Understanding the pathways that control NLRP3 activation will help develop strategies to treat these diseases. Here we identify WNK1 as a negative regulator of the NLRP3 inflammasome. Macrophages deficient in WNK1 protein or kinase activity have increased NLRP3 activation and pyroptosis compared with control macrophages. Mice with conditional knockout of WNK1 in macrophages have increased IL-1β production in response to NLRP3 stimulation compared with control mice. Mechanistically, WNK1 tempers NLRP3 activation by balancing intracellular Cl^–^ and K^+^ concentrations during NLRP3 activation. Collectively, this work shows that the WNK1 pathway has a critical function in suppressing NLRP3 activation and suggests that pharmacological inhibition of this pathway to treat hypertension might have negative clinical implications.

## Abstract (web summary)

The serine/threonine kinase WNK1 is an inhibitor of chloride efflux. Here the authors show that this inhibition is a means of negatively regulating the activation of the NLRP3 inflammasome in macrophages, leading to reduced inflammatory responses.

## Introduction

The NLRP3 inflammasome is an intracellular multi-protein complex that assembles and mounts an inflammatory immune response following exposure to pathogens and tissue damage. This complex is primarily found in macrophages and consists of NLRP3 protein, ASC adapter protein, and the precursor to caspase-1, procaspase-1. NLRP3 protein (Nod-like receptor protein 3) serves as a “sensor protein” by detecting cellular changes caused by pathogen-associated molecular patterns (PAMPs) from microbial components and danger-associated molecular patterns (DAMPs) from endogenous signals related to cellular injury^1,2^. Canonical activation of the NLRP3 inflammasome requires two steps—the priming step and the activation step. In the priming step, Toll-like receptors (TLRs) are primed by TLR agonists such as lipopolysaccharide (LPS). This induces NF-κB-mediated NLRP3 and pro-IL-1β expression^3^, but also importantly causes several posttranslational modifications of NLRP3 that are required for its activation^4–8^. In the second signal, NLRP3 is activated by a variety of ligands including uric acid, cholesterol, silica, aluminum salts, amyloid deposits, and various microbial products^9^. These diverse stimuli are believed to activate NLRP3 through several different mechanisms such as K^+^ efflux, Cl^–^ efflux, Ca^2+^ signaling, lysosomal destabilization, and reactive oxygen species (ROS) production^2,10^. Recent work showed that these NLRP3 stimuli cause disassembly of the trans-Golgi network (TGN), resulting in dispersed TGN (dTGN)^11^. NLRP3 is then recruited to dTGN through ionic binding to phosphatidylinositol 4-phosphate (PtdIns4P) on the surface of dTGN, providing a platform for NLRP3 to oligomerize^11^. Importantly, this work demonstrates that although NLRP3 stimuli are diverse, dTGNs may be the common signal to activate NLRP3. Following NLRP3’s localization to dTGN, oligomerized NLRP3 is transported via HDAC6-dynein machinery to the microtubule-organizing center (MTOC) for final inflammasome assembly^12^ with ASC adapter protein, recruiting procaspase-1. NEK7 has also been shown to be required for NLRP3 assembly^13–16^, likely bridging NLRP3 subunits in the complex^17^. Once recruited to the complex, procaspase-1 is autoproteolytically cleaved to its active caspase-1 form. Activation of caspase-1 is considered the point of no return in the NLRP3 inflammatory process. Once active, caspase-1 cleaves and activates interleukin-1β (IL-1β) and interleukin-18 (IL-18), which trigger a proinflammatory response. In addition to activating cytokines, caspase-1 causes an inflammatory form of cell death called pyroptosis, via cleavage of a protein called gasdermin D (GSDMD)^18,19^. This cleavage generates an N-terminal fragment that localizes to the plasma membrane and forms pores, causing physical rupture of the cell and release of the activated IL-1β and IL-18. This release of cytoplasmic contents from dying cells provides signals to initiate an inflammatory cascade for clearance of pathogens from the host^18,20^.

The NLRP3 inflammasome is a central regulator of inflammatory processes; however, excessive inflammation caused by aberrant NLRP3 inflammasome activity is attributed to several diseases such as inflammatory bowel diseases, atherosclerosis, rheumatoid arthritis, gout, type 2 diabetes, and Alzheimer’s disease^21^. Despite mounting research on NLRP3 inflammasome involvement in human health and disease, we still do not understand the exact molecular mechanisms that control NLRP3 activation. Therefore, there is a pressing need to fill this gap in our knowledge of NLRP3 regulation to effectively develop novel strategies to treat NLRP3-related inflammatory diseases.

With no lysine [K] (WNK) serine/threonine kinases are a subfamily of kinases found in all mammals that were named from their lack of a catalytic lysine in subdomain II that is typically conserved in kinases for binding to ATP^22,23^. During osmotic stress or low intracellular Cl^–^, WNK is activated and phosphorylates effector kinases SPS/Ste20-related proline-alanine-rich kinase (STK39) and oxidative stress responsive 1 (OXSR1). STK39 and OXSR1 control the activity of the SLC12 family of cation-Cl^–^ cotransporters, thus regulating cellular ion flux, namely Na^+^, K^+^, and Cl^–^ ^24–26^. The WNK signaling pathway is critical for maintaining blood pressure and kidney homeostasis, and gain of function mutations of WNK1 and WNK4 are responsible for several forms of hypertension^27,28^. Although WNKs were formerly primarily studied in the cardiovascular and renal fields, WNK1 has gained popularity due to its involvement in a wide variety of other processes including, but not limited to, regulation of cell volume^29^, neuronal Cl^–^ homeostasis^29,30^, autophagy^31^, cancer^32–34^, and mitosis and abscission^23^. Interestingly, WNK1 also inversely and independently balances both T cell adhesion and migration^35^, demonstrating the first published evidence implicating a role for WNK in the immune system. Moreover, a recent study revealed a new role for WNK1 signaling in the removal of apoptotic cells by efferocytosis^36^. The study demonstrated that disruption of WNK1 or its downstream target SLC12A2 cotransporter results in a significant increase in apoptotic corpse uptake and a switch from an anti-inflammatory to a pro-inflammatory response in phagocytes^36^, suggesting a role for WNK1 signaling in suppressing inflammation.

As K^+^ and Cl^–^ effluxes are critical steps in NLRP3 inflammasome activation by pore-forming stimuli^37,38^, here we investigate whether the WNK kinases that function as major regulators of intracellular ion homeostasis and cell volume are also involved in the regulation of NLRP3 inflammasome activation in macrophages. We show that WNK1 suppresses NLRP3-dependent inflammation by dampening NLRP3 inflammasome activation through its action on cation-Cl^–^cotransporters, thus adding another layer of regulation to the NLRP3 activation pathway.

## Results

### Inhibition of WNK1 increases NLRP3 inflammasome activation

**Fig. 1:**
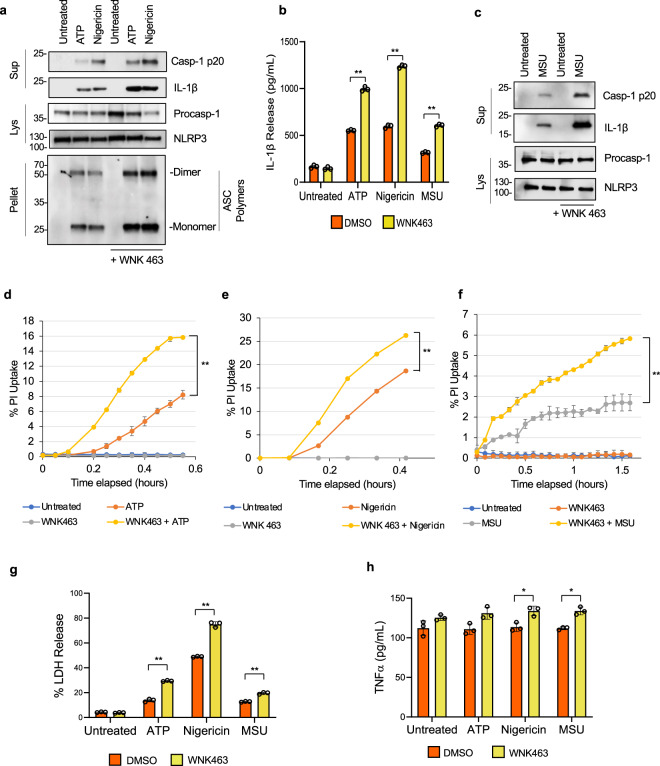
WNK inhibition by WNK463 increases NLRP3 inflammasome activation in macrophages. **a** Immunoblots of caspase-1 p20 and mature IL-1β released in culture supernatants (Sup), procaspase-1 and NLRP3 in cell lysates (Lys) or ASC in NP40-insoluble pellets (Pellet) of LPS-primed (4 h) primary wild type bone marrow macrophages pretreated with or without 1 µM WNK 463 inhibitor for 10 m followed by 5 mM ATP or 10 µM nigericin as indicated. **b** IL-1β release in culture supernatants of LPS-primed (4 h) primary wild type bone marrow macrophages treated with or without WNK 463 inhibitor followed by ATP, nigericin, or MSU stimulation as indicated. *P* values are 0.383, 0.00052, 0.00055, and 0.0011. **c** Immunoblots of caspase-1 p20 and mature IL-1β released in culture supernatants (Sup) or procaspase-1 and NLRP3 in cell lysates (Lys) of LPS-primed (4 h) primary wild type bone marrow macrophages treated with or without 1 µM WNK 463 inhibitor for 10 m followed by MSU. Propidium iodide uptake of LPS-primed (4 h) primary BMDMs treated with or without WNK 463 inhibitor followed by ATP (**d**), nigericin (**e**) or MSU (**f**) stimulation as measured on the IncuCyte over time. *P* value of **d** is 0.0032; **e** is 0.0037; **f** is 0.0035. LDH (**g**) and TNFα (**h**) release in culture supernatants of LPS-primed (4 h) primary wild type bone marrow macrophages treated with or without WNK 463 inhibitor followed by ATP, nigericin, or MSU stimulation as indicated. *P* values in **g** are 0.292, 0.00132, 0.00149, and 0.00132; **h** are 0.1512, 0.1249, 0.0303, and 0.0165. Results are representative of at least three independent experiments performed in duplicate or triplicate. Error bars in **b**, **d**–**h** are presented as mean values ± standard deviation (S.D.), with *n* = 3. Two-sided Student’s *t* test, *p < 0.05, **p < 0.005.

K^+^ and Cl^−^ efflux are critical steps for NLRP3 inflammasome activation by pore-forming stimuli^16,38–41^. To investigate whether the so-called with-no-lysine (WNK) kinases, which function as major regulators of intracellular ion homeostasis and cell volume^29,42,43^, are involved in the regulation of NLRP3 activation in macrophages, we assessed the effect of pharmacological inhibition of WNK kinases on inflammasome activation. We treated LPS-primed primary bone marrow-derived macrophages (BMDMs) with ATP, nigericin, or monosodium urate (MSU) crystals in the presence or absence of the selective pan WNK kinases inhibitor WNK463^42,44^. We observed a significant increase in NLRP3 activation as evidenced by increased caspase-1 activation, IL-1β production, and ASC oligomerization in cells treated with ATP (Fig. 1a, b), nigericin (Fig. 1a, b), or MSU (Fig. 1b, c) in the presence of WNK463 compared with the control treatments. We also observed an increase in propidium iodide uptake (Fig. 1d–f) as well as an increase in lactate dehydrogenase (LDH) release (Fig. 1g) in cells treated with ATP, nigericin, or MSU in the presence of WNK463 compared with controls, indicating increased pyroptosis. Although WNK463 treatment alone induced significant increase in TNF production without inflammasome activation (Fig. 1h), inflammasome activation with ATP, nigericin or MSU did not further increase TNF production. This effect of WNK463 on TNF appears to be specific for this drug and was not observed when BMDMs were treated with WNK1 inhibitor 11 or when their WNK1 was deleted (see below).

Intrigued by these findings, we next aimed to investigate which of the four WNK kinases plays a dominant role in the negative regulation of NLRP3 activation in macrophages. As WNK1 is expressed in most tissues^45^ and has been shown to play a role in the immune system by regulating T cell activities^35^, we tested the effect of a recently developed specific WNK1 inhibitor 11 (WNK-IN-11)^46^ on NLRP3 inflammasome activation in LPS-primed primary BMDMs. Similar to WNK463, WNK-IN-11 increased NLRP3 inflammasome activation as measured by caspase 1 activation and IL-1β production in cell culture supernatants treated with ATP, nigericin, or MSU (Supplementary Fig. 1a, b). WNK-IN-11 treatment also increased propidium iodide uptake and LDH release in BMDMs treated with ATP, nigericin, or MSU compared to control cells (Supplementary Fig. 1d-g). WNK-IN-11 had no effect on TNF production (Supplementary Fig. 1h).

To investigate whether WNK1 inhibition increases NLRP3 activation by directly affecting its oligomerization, we treated immortalized NLRP3-KO BMDMs stably expressing EGFP-tagged NLRP3 (NLRP3-EGFP) with ATP or nigericin in the presence or absence of WNK-IN-11. We observed a significant increase in the number of cells containing NLRP3 specks in WNK-IN-11-treated compared to control DMSO treated cells (Supplementary Fig. 2). Together, these results indicate that WNK1 plays a dominant inhibitory role in NLRP3 inflammasome activation and its inhibition augments inflammasome activation by increasing NLRP3 oligomerization.

### Conditional knockout of WNK1 increases NLRP3 inflammasome activation by NLRP3 stimuli

To further address the role of WNK1 in the regulation of NLRP3 inflammasome activation, we attempted to generate immortalized WNK1 knockout macrophage cell lines by CRISPR/Cas9 approaches, as complete *Wnk1* deletion in mice is lethal due to defects in cardiovascular development^47^. To this end we transduced immortalized wild type and NLRP3-KO BMDM lines with sgRNA targeting *Wnk1*. To our surprise, we were not able to achieve a complete WNK1 knockout in immortalized wild type BMDMs, suggesting a loss of WNK1 is unfavorable for immortalized macrophage survival. Interestingly, complete WNK1 knockout lines were obtained in immortalized NLRP3-KO BMDMs (Table 1). Similarly, complete WNK1 knockout lines were obtained in immortalized ASC-KO or caspase-1-KO BMDMs but not in immortalized RIPK1-KO BMDMs (Table 1). As WNK1 inhibition increased inflammasome-induced pyroptosis (Fig. 1, Supplementary Fig. 1), these results suggest that WNK1 deletion in immortalized BMDMs containing functional NLRP3 inflammasome likely leads to pyroptotic cell death and highlights the importance of WNK1 activity in cells with functional NLRP3 inflammasome.

**Table 1:** Wnk1 knockout is lethal in immortalized macrophages with fully functional NLRP3 inflammasome activity.

	#Clones tested	#WT clones	#Het clones	#KO clones
Wild type	31	27	4	0
Rip1 KO	36	31	5	0
NLRP3 KO	18	10	5	3
Caspase 1 KO	18	14	1	3
ASC KO	36	20	8	8

**Fig. 2:**
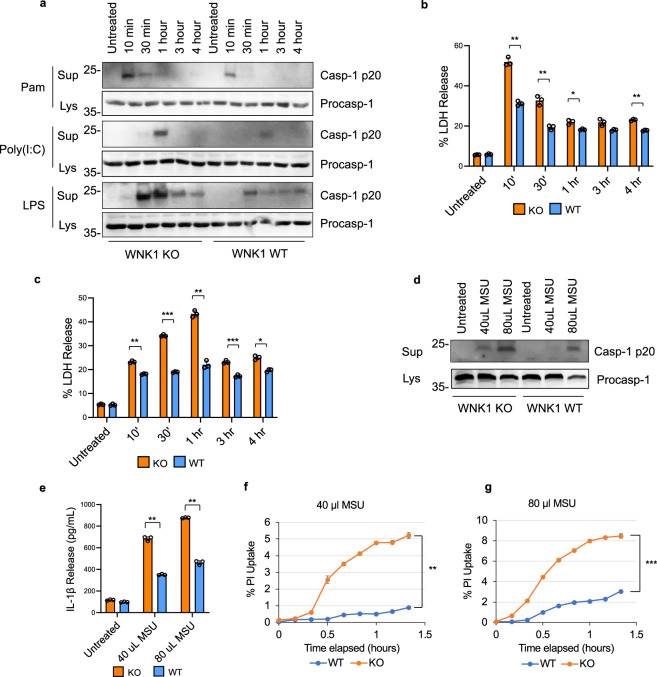
Tamoxifen-induced WNK1 knockout increases NLRP3 inflammasome activation in macrophages. **a** Immunoblots of caspase-1 in culture supernatant (Sup) and cell lysates (Lys) of immortalized *Wnk1*^*flox/flox*^-CreERT2^+^ (WNK1 KO) or *Wnk1*^*+/+*^ (WT) macrophages treated with tamoxifen for 48 h prior to stimulation with Pam3CSK4, Poly(I:C) or LPS for the indicated times followed by ATP (45 min). LDH release of immortalized *Wnk1*^*flox/flox*^-CreERT2^+^ (WNK1 KO) or *Wnk1*^*+/+*^ (WT) macrophages treated with tamoxifen for 48 h prior to stimulation with Pam3CSK4 (**b**) or Poly(I:C) (**c**) for the indicated times followed by ATP (45 min). *P* values for **b** are 0.321, 0.00473, 0.0036, 0.0475, 0.0754, and 0.0027; **c** are 0.716, 0.0055, 0.0004, 0.0006, 0.0001, and 0.0191. **d** Immunoblots of caspase-1 in culture supernatant (Sup) and cell lysates (Lys) of immortalized Wnk1^flox/flox^-CreERT2^+^ (WNK1 KO) or *Wnk1*^*+/+*^ (WT) macrophages treated with tamoxifen for 48 h prior to stimulation with LPS for 3 h followed by MSU (40 uL or 80 uL 5 mg mL^−^ MSU) for 80 m. IL-1β release (**e**) and propidium iodide uptake (**f**, **g**) of immortalized Wnk1^flox/flox^-CreERT2^+^ (WNK1 KO) or *Wnk1*^*+/+*^ (WT) macrophages treated with tamoxifen for 48 h prior to stimulation with LPS for 3 h followed by MSU (40 uL (**f**) or 80 uL (**g**) 5 mg mL^−^ MSU) for 80 m. *P* values for **e** are 0.061, 0.00077, and 0.00094; **f** is 0.0005; **g** is 0.00034. Results are representative of at least three independent experiments performed in duplicate or triplicate. Error bars in **b**, **c**, **e**–**g** are presented as mean values ± standard deviation (S.D.), with *n* = 3. Two-sided Student’s *t* test, *p < 0.05, **p < 0.005, ***p < 0.0005.

As we were unable to obtain immortalized WNK1 KO BMDMs with functional NLRP3 inflammasome, we generated immortalized BMDMs (iBMDMs) from bone marrow isolated from mice carrying a floxed *Wnk1* gene under the control of the tamoxifen-inducible CreERT2 (Wnk1^-/flox^-CreERT2^+^)^35^. Upon treatment of these iBMDMs with tamoxifen for 48 h, WNK1 was deleted (Supplementary Fig. 3). To assess the effect of conditional WNK1 deletion on NLRP3 inflammasome activation, we primed tamoxifen-treated wild type and *Wnk1*^*-/flox*^-CreERT2^+^ iBMDMs with Pam3CSK4, Poly I:C, or LPS followed by ATP (Fig. 2a). Regardless of the priming pathway activated by these stimuli, whether it is short (Pam3CSK4, 10 min), intermediate (poly I:C, 30–60 min) or long (LPS, 10–240 min)^7,8^, we observed a significant increase in NLRP3 activation in the tamoxifen-induced WNK1 knockout cells compared to control tamoxifen-treated wild type cells. This indicates that WNK1 deletion does not affect the NLRP3 priming step (signal 1), but it augments the NLRP3 activation step (signal 2). This increase was associated with increased LDH release (Fig. 2b, c), indicating increased pyroptosis. Similarly, increased caspase 1 activation (Fig. 2d), IL-1β release (Fig. 2e), and PI uptake (Fig. 2f, g) were seen after stimulation of tamoxifen-treated and LPS-primed *Wnk1*^*-/flox*^-CreERT2^+^ iBMDMs with MSU, in a dose-dependent manner. Similar results were seen in primary *Wnk1*^*-/flox*^-CreERT2^+^ BMDMs (not shown). We also observed an increase in NLRP3 activation in tamoxifen-induced *Wnk1*^*-/flox*^-CreERT2^+^ cells (homozygous knockout) versus *Wnk1*^*flox/+*^-CreERT2^+^ cells (heterozygous), indicating that one copy of *Wnk1* gene is sufficient to prevent excessive NLRP3 activation (Supplementary Fig. 4). Altogether, these results validate our WNK inhibitors data and underscore the importance of WNK1 in the regulation of NLRP3 inflammasome activation.

**Fig. 3:**
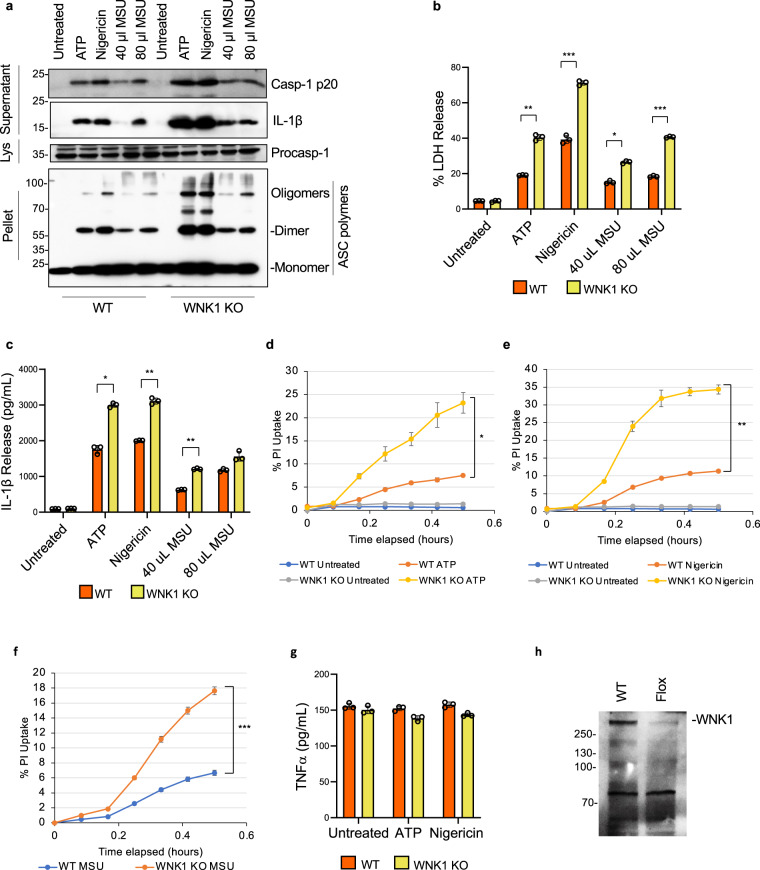
Conditional WNK1 knockout increases NLPR3 inflammasome activation in macrophages. **a** Immunoblots of caspase-1 p20 and mature IL-1β released in culture supernatants (Supernatant), procaspase-1 in cell lysates (Lys) or ASC in NP40-insoluble pellets (Pellet) of LPS-primed (4 h) primary *Wnk1*^*+/+*^ LysMCre^*+*^ (WNK1 WT) and *Wnk1*^*flox/flox*^ LysMCre^*+*^ (WNK1 KO) BMDMs treated with ATP (5 mM), nigericin (10 µM), or MSU (40 ul or 80 ul 5 mg ml^−^) as indicated. LDH (**b**) and IL-1β (**c**) release in culture supernatants of LPS-primed (4 h) primary *Wnk1*^*+/+*^ LysMCre^*+*^ (WNK1 WT) and *Wnk1*^*flox/flox*^ LysMCre^*+*^ (WNK1 KO) BMDMs treated with ATP, nigericin, or MSU (40 ul or 80 ul 5 mg ml^−^ MSU) as indicated. *P* values of **b** are 0.6525, 0.00177,0.00044, 0.00618, and 0.00017; **c** are 0.0536, 0.00595, 0.0026, 0.0008, and 0.0602. Propidium iodide uptake of LPS-primed (4 h) primary *Wnk1*^*+/+*^ LysMCre^*+*^ (WNK1 WT) and *Wnk1*^*flox/flox*^ LysMCre^*+*^ (WNK1 KO) BMDMs treated with ATP (**d**), nigericin (**e**), or MSU (**f**). *P* value of **d** is 0.0072; **e** is 0.00065; **f** is 0.00004. **g** TNFα release in culture supernatants of LPS-primed (4 h) primary *Wnk1*^*+/+*^ LysMCre^*+*^ (WNK1 WT) and *Wnk1*^*flox/flox*^ LysMCre^*+*^ (WNK1 KO) BMDMs treated with ATP or nigericin. **h** Immunoblot of WNK1 in cell lysates of *Wnk1*^*+/+*^ LysMCre^*+*^ (WNK1 WT) and *Wnk1*^*flox/flox*^ LysMCre^*+*^ (WNK1 KO) BMDMs. Results are representative of at least three independent experiments performed in duplicate or triplicate. Error bars in **b**–**g** are presented as mean values ± standard deviation (S.D.), with *n* = 3. Two-sided Student’s *t* test, *p < 0.05, **p < 0.005, ***p < 0.0005.

To develop a model without the use of tamoxifen and to allow for assessment of WNK1 deficiency on NLRP3 inflammasome activation in vivo, we generated mice carrying a floxed *Wnk1* gene under the control of the myeloid-specific Cre recombinase LysMCre. These mice delete *Wnk1* only in fully differentiated macrophages without disturbing cardiovascular development. The WNK1 conditional knockout mice (*Wnk1*^*flox/flox*^ LysMCre^*+*^) survived and were phenotypically indistinguishable from their wild type siblings. BMDMs isolated from these mice were primed with LPS and treated with ATP, nigericin, or MSU and analyzed for caspase 1 activation and ASC oligomerization (Fig. 3a), LDH release (Fig. 3b), IL-1β release (Fig. 3a, c), and PI uptake (Fig. 3d–f). Consistent with our results with the WNK1 inhibitors and tamoxifen-induced *Wnk1* deletion, we observed a significant increase in NLRP3 activation and pyroptosis in conditional WNK1 KO BMDMs compared to their wild type controls (Fig. 3a–f). This increase of NLRP3 activation and pyroptosis was observed under conditions of short priming with LPS or Pam3CSK4 (10 min) or long priming with LPS (4 h) (Supplementary Fig. 5). Collectively, these data further validate the crucial role of WNK1 in suppressing NLRP3 inflammasome activation in macrophages.

It was shown previously that imiquimod may activate NLRP3 by a K^+^ efflux-independent mechanism^48^. Our results demonstrate that imiquimod-induced NLRP3 activation as measured by caspase-1 activation, IL-1β production and pyroptosis in BMDMs is enhanced by deletion or inhibition of WNK1 (Supplementary Fig. 6). These results indicate that imiquimod-induced NLRP3 activation is also regulated by WNK1.

To investigate the specificity of WNK1 regulation of the NLRP3 inflammasome, we tested the impact of WNK1 inhibition or deletion on other inflammasomes such as NLRC4, NLRP1, non-canonical caspase-11 and AIM2. Our results show that WNK1 inhibition or deletion does not impact the activity of the NLRC4, NLRP1 and non-canonical caspase-11 inflammasomes (Supplementary Figs. 7–9). However, we observed that WNK1 inhibition or deletion increases the activity of the AIM2 inflammasome (Supplementary Fig. 10). Nevertheless, this increased AIM2 activation, unlike the case with the NLRP3 inflammasome (see below), was not regulated by Cl^−^ or K^+^ (Supplementary Fig. 11). Future research should provide more insight on the mechanism of regulation of the AIM2 inflammasome by WNK1.

### The WNK1/ OXSR1/ STK39 signaling pathway regulates NLRP3 activation

**Fig. 4:**
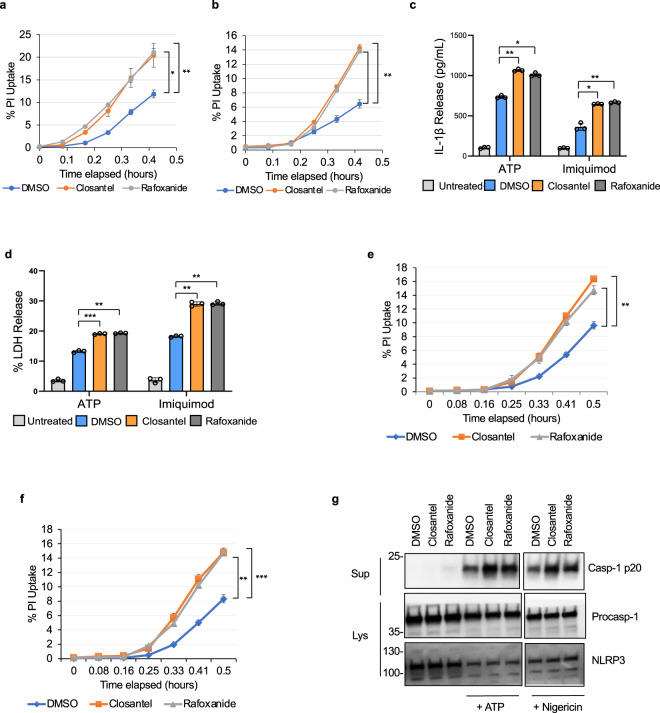
Pharmacological inhibition of STK39/OXSR1 increases NLRP3 inflammasome activation in macrophages. Propidium iodide uptake of LPS-primed (4 h) primary wild type BMDMs treated with or without 1 uL of DMSO or 2 µM Closantel or Rafoxanide for 10 m prior to treatment with ATP (**a**) or imiquimod (**b**). *P* values for **a** are 0.011432 and 0.00492; **b** are 0.00162 and 0.00091. IL-1β (**c**) and LDH (**d**) release in culture supernatants of LPS-primed (4 h) primary wild type bone marrow macrophages treated with or without 1 uL of DMSO or 2 µM Closantel or Rafoxanide for 10 m prior to treatment with ATP or imiquimod. *P* values for **c** are 0.00291, 0.00585, 0.00707, and 0.0048; **d** are 0.00042, 0.00129, 0.00162, and 0.00071. Propidium iodide uptake of LPS-primed (15 m) immortalized wild type BMDMs treated with or without 1 uL of DMSO or 2 µM Closantel or Rafoxanide for 10 m prior to treatment with ATP (**e**) or imiquimod (**f**). *P* values for **e** are 0.00126 and 0.00149; **f** are 0.00164 and 0.000296. **g** Immunoblots of caspase-1 p20 released in culture supernatants (Sup) or procaspase-1 and NLRP3 in cell lysates (Lys) of LPS-primed (15 m) treated with or without 1 uL of DMSO or 2 µM Closantel or Rafoxanide for 10 m prior to treatment with ATP or nigericin as indicated. The split caspase 1 p20 represents two exposures of the same membrane. Results are representative of at least three independent experiments performed in duplicate or triplicate. Error bars in **a**–**f** are presented as mean values ± standard deviation (S.D.), with *n* = 3. Two-sided Student’s *t* test, *p < 0.05, **p < 0.005, ***p < 0.0005.

WNK1 regulates intracellular ion concentrations by activating oxidative stress responsive 1 (OXSR1) and STE20/SPS1-related proline/alanine-rich (STK39) kinases, which regulate intracellular Na^+^, K^+^ and Cl^−^ ion flux by acting on plasma membrane Na^+^/K^+^-Cl^−^ co-transporters^29,49^. Considering the important role of Cl^−^ and K^+^ efflux in NLRP3 activation^16,38–41^, we investigated whether WNK1 regulation of NLRP3 activation is dependent on its canonical OXSR1/ STK39 signaling pathway. We used two different inhibitors, Rafoxanide and Closantel, which inhibit both STK39 and OXSR1 kinases through binding to an allosteric site on their C-terminal domains^50^. Pretreatment of LPS-primed primary wild type BMDMs with Closantel or Rafoxanide for 10 m prior to NLRP3 stimulation with ATP (Fig. 4a) or imiquimod (Fig. 4b) resulted in more propidium iodide uptake compared with vehicle controls. These cells also showed more IL-1β production (Fig. 4c) and LDH release (Fig. 4d) than the vehicle controls. Similar results were obtained when these inhibitors were tested in immortalized wild type BMDMs (Fig. 4e–g). Together, these results indicate that canonical WNK1/OXSR1/STK39 kinase signaling is required to control NLRP3 inflammasome activation.

Activation of OXSR1/STK39 pathway results in phosphorylation and activation of their target cation-Cl^−^ co-transporters, which in turn stimulate cation (Na^+^/K^+^) and Cl^−^ uptake^22,25,41,42^ (Supplementary Fig. 12a). Loop and thiazide diuretics are selective inhibitors of several cation-Cl^−^ co-transporters and are thus prescribed to block WNK signaling to control high blood pressure in hypertension patients^44,45^. Notably, both thiazide and loop diuretics have been shown to increase the risk of developing gout, an inflammatory disease mediated by NLRP3^51^, perhaps because of their ability to elevate uric acid in the blood^52^. Interestingly, we found that treatment of LPS-primed BMDMs with Hydrochlorothiazide (HCT, a thiazide diuretic and a selective inhibitor of the SLC12A3 cotransporter) enhances activation of the NLRP3 inflammasome and pyroptosis by ATP (Supplementary Fig. 12b, c, e) and MSU (Supplementary Fig. 12d, f). As MSU crystals have been found in body fluids of patient on thiazide drugs^52^, our finding suggests that inhibition of cation-Cl^−^ co-transporter downstream of OXSR1/STK39 may increase the severity of gout in these patients by increasing NLRP3 inflammasome activation.

### WNK1 regulates NLRP3 activation by a Cl^−^ sensing mechanism

**Fig. 5:**
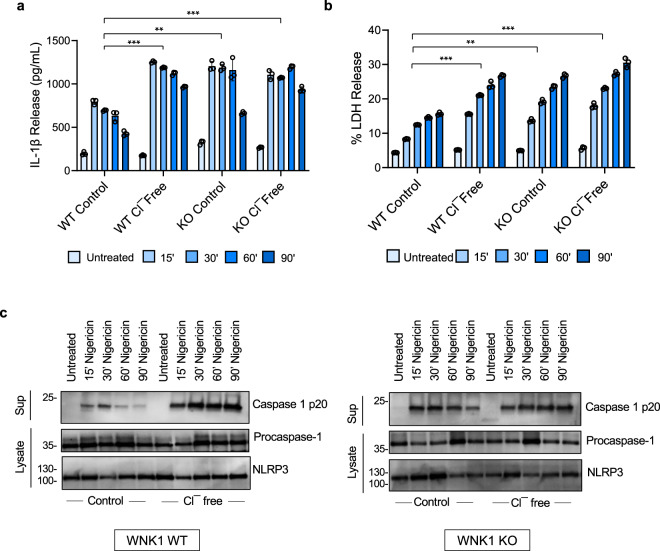
WNK1 regulates NLRP3 inflammasome activation by a Cl^−^-sensing mechanism. IL-1β (**a**) and LDH (**b**) release in culture supernatants of LPS-primed (4 h) primary *Wnk1*^*+/+*^ LysMCre^*+*^ (WNK1 WT) and *Wnk1*^*flox/flox*^ LysMCre^*+*^ (WNK1 KO) BMDMs reperfused with the indicated isotonic salt solution for 1 h prior to stimulation with nigericin for the indicated times. *P* values in a are 0.000255, 0.001413, and 0.00003; **b** are 0.00018, 0.00229, and 0.000208. **c** Immunoblots of caspase-1 p20 released in culture supernatants (Sup) or procaspase-1 and NLRP3 in cell lysates (Lysate) of LPS-primed (4 h) primary *Wnk1*^*+/+*^ LysMCre^*+*^ (WNK1 WT) and *Wnk1*^*flox/flox*^ LysMCre^*+*^ (WNK1 KO) BMDMs reperfused with the indicated isotonic salt solution for 1 h prior to stimulation with nigericin for the indicated times. Results are representative of at least three independent experiments performed in duplicate or triplicate. Error bars in **a** and **b** are presented as mean values ± standard deviation (S.D.). with *n* = 3. Two-sided Student’s *t* test, **p < 0.005, ***p < 0.0005.

WNK1 has been shown to directly sense low intracellular Cl^−^ concentration leading to its activation^53^. Once activated, WNK1 phosphorylates OXSR1 and STK39 kinases, which in turn phosphorylate and activate the SLC12A2 (NKCC1), SLC12A1 (NKCC2) and SLC12A3 (NCC) Na^+^/K^+^/Cl^−^ co-transporters to maintain normal intracellular Na^+^, K^+^ and Cl^−^ ion concentrations^29,49^. Considering that Cl^−^ and K^+^ effluxes play important roles in NLRP3 activation^16,38,40^, we decided to test the hypothesis that WNK1 dampens NLRP3 activation by activating the cation-Cl^−^ cotransporters when it senses a drop in intracellular Cl^−^. The activated co-transporters offset Cl^−^ and K^+^ loss by mediating Cl^−^ and K^+^ influx from the extracellular medium. Accordingly, WNK1 will not be able to offset Cl^−^ and K^+^ loss when wild type BMDMs are stimulated in a Cl^−^-free medium, because cation-Cl^−^ cotransporters have an absolute requirement for Cl^−^ for their activity. Supporting this hypothesis, LPS-primed wild type BMDMs showed significantly higher IL-1β production, LDH release and caspase-1 activation (Fig. 5a–c) when stimulated with nigericin in Cl^−^-free media compared to control media. In addition, stimulation of LPS-primed WNK1 deficient BMDMs, which are expected to have no cotransporter activity, with nigericin in a Cl^−^-free medium caused no increase in IL-1β production, LDH release or caspase 1 activation above that seen in a normal control medium (Fig. 5). In fact, the levels of inflammasome activation as quantified by IL-1β and LDH release in WNK1 KO cells were similar to those seen in wild type cells in Cl^−^-free media (Fig. 5a, b), indicating that the increased activation of the NLRP3 inflammasome in WNK1-KO compared to wild type BMDMs in normal medium is due to inhibition of the co-transporters activity as a result of WNK1 deletion. These results demonstrate that inhibition of cotransporters activity by incubation in Cl^−^-free medium can produce the same effect on NLRP3 activation as deletion/inhibition of WNK1. Similar results were obtained when these experiments were repeated in wild type cells in the presence or absence of the WNK1 inhibitor WNK-IN-11 (Supplementary Fig. 13). Consistent with the above results, intracellular K^+^ levels were significantly lower in WT cells incubated in a Cl^−^-free medium compared with normal medium (Supplementary Fig. 14a). In contrast intracellular K^+^ levels in WNK1-KO cells were similar in Cl^−^-free and normal media (Supplementary Fig. 14b). Overall, these results demonstrate that WNK1 suppresses NLRP3 inflammasome activation by activating cation-Cl^−^ cotransporters when it senses low intracellular Cl^−^.

**Fig. 6:**
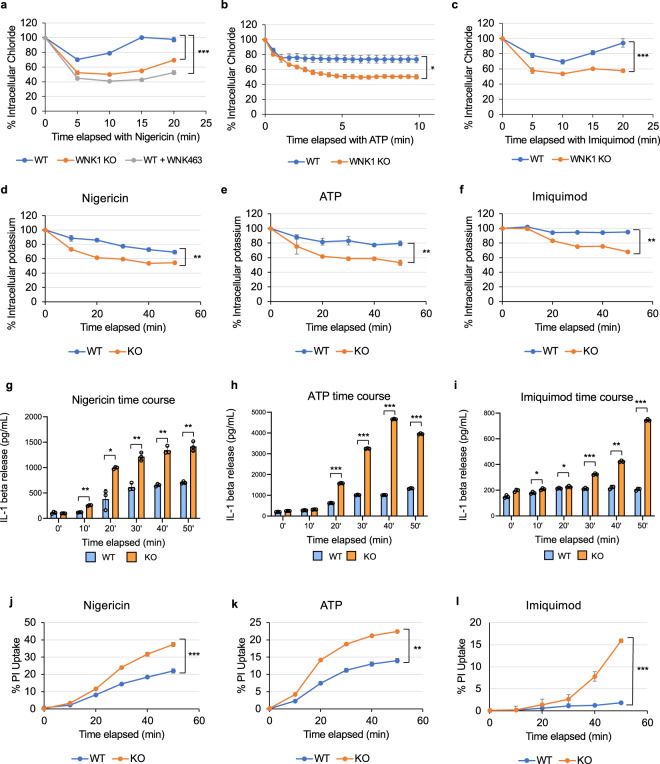
WNK1 regulates intracellular Cl^−^ in macrophages. Quantification of intracellular Cl^−^ (**a**–**c**), K^+^ (**d**–**f**), IL-1β release (**g**–**i**) and propidium iodide uptake (**j–l**) in LPS-primed (4 h) primary *Wnk1*^*+/+*^ LysMCre^*+*^ (WNK1 WT), *Wnk1*^*flox/flox*^ LysMCre^*+*^ (WNK1 KO), or WNK463-treated wild type BMDMs following treatment with nigericin (**a**, **d**, **g**, **j**), ATP (**b**, **e**, **h**, **k**), or Imiquimod (**c**, **f**, **i**, **l**) in normal medium. *P* values of **a** 0.001839, 0.00135; **b** 0.01758; **c** 0.001793; **d** 0.001072; **e** 0.00327; **f** 0.00135; **g** 0.4217, 0.00072976, 0.0323, 0.00101, 0.00219, and 0.00345; **h** 0.058, 0.0594, 0.000284, 0.000056, 0.0000013, and 0.0000127; **i** 0.0556, 0.0146, 0.01339, 0.0000265, 0.000953, and 0.000084; **j** 0.00000030; **k** 0.00242; **l** 0.000111. Results are representative of at least three independent experiments performed in duplicate or triplicate. Error bars in **a**–**l** are presented as mean values ± standard deviation (S.D.), with *n* = 3. Two-sided Student’s *t* test, *p < 0.05, **p < 0.005, ***p < 0.0005.

The importance of cation-Cl^−^ cotransporters in WNK1-mediated regulation of NLRP3 activation was further validated by measuring intracellular Cl^−^ using MQAE, a fluorescent Cl^−^ ion indicator. At resting state both wild type and conditional WNK1 knockout BMDMs had similar levels of intracellular Cl^−^ as measured by fluorescence (Supplementary Fig. 15a). When treated with NLRP3 stimuli nigericin, ATP or imiquimod, but not AIM2 inflammasome activator dA:dT, wild type BMDMs had significantly lower loss of intracellular Cl^−^ compared to WNK1 KO BMDMs (Fig. 6a–c, Supplementary Fig. 15b). Wild type BMDMs had a loss of ~25% of their intracellular Cl^−^ by 5 min after nigericin or ATP treatment (Fig. 6a, b), and about 30% of their intracellular Cl^−^ by 10 min after imiquimod treatment (Fig. 6c). In contrast, conditional WNK1 KO cells as well as wild type BMDMs treated with WNK463 lost about 50% of their Cl^−^ by five to 10 min (Fig. 6a–c). The effect of NLRP3 stimuli on intracellular Cl^−^ levels is independent of NLRP3 status, as NLRP3 knockout BMDMs treated with nigericin in the presence or absence of WNK1 inhibitor showed a similar pattern of Cl^−^ loss as observed in wild type BMDMs treated similarly (Supplementary Fig. 15c). Since WNK1-regulated K^+^ flux via cation-Cl^−^ cotransporters is coupled to Cl^−^ flux (Supplementary Fig. 16), there was also a greater loss of intracellular K^+^ in WNK1-KO BMDMs compared to WT controls stimulated with the same NLRP3 stimuli (Fig. 6d–f). These results demonstrate that without WNK1, BMDMs are not able to offset their intracellular Cl^−^ and K^+^ loss during NLRP3 activation because of their inability to regulate their cation-Cl^−^ cotransporters (Supplementary Fig. 16), which explains increased NLRP3 activation and pyroptosis (Fig. 6g–l).

### K^+^ and Cl^−^ effluxes drive NLRP3 activation in WT and WNK1 KO cells

**Fig. 7:**
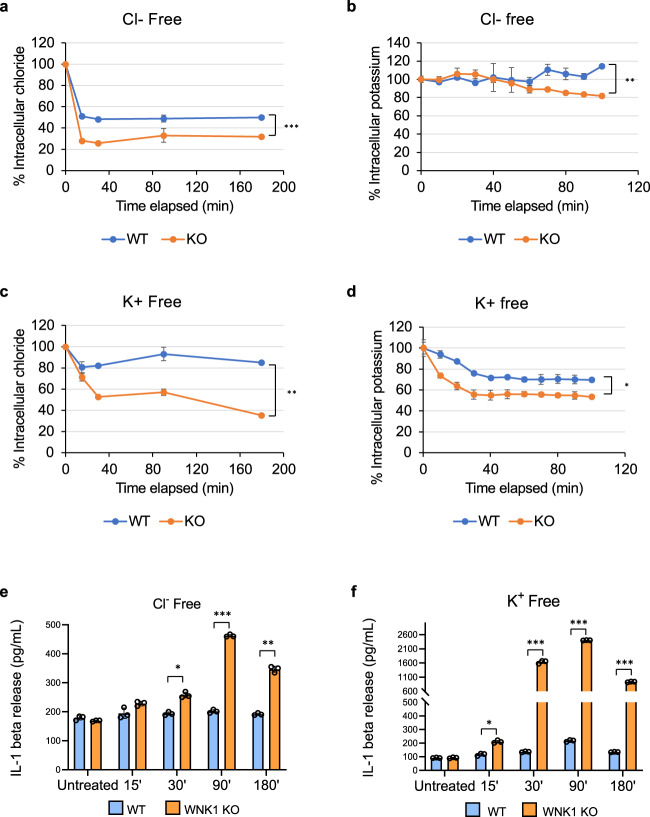
Potassium and Cl^−^ effluxes drives NLRP3 activation in WT and WNK-KO cells. **a**, **c** Quantification of intracellular Cl^−^ of LPS-primed (4 h) primary *Wnk1*^*+/+*^ LysMCre^*+*^ (WNK1 WT) and *Wnk1*^*flox/flox*^ LysMCre^*+*^ (WNK1 KO) BMDMs in Cl^−^ free (**a**) or K^+^ free (**b**) media. Quantification of intracellular K^+^ of LPS-primed (4 h) primary *Wnk1*^*+/+*^ LysMCre^*+*^ (WNK1 WT) and *Wnk1*^*flox/flox*^ LysMCre^*+*^ (WNK1 KO) BMDMs in Cl^−^ free (**b**) or K^+^ free (**d**) media. *P* value of **a** is 0.000288; **b** is 0.003453; **c** is 0.00227. Measurement of IL-1β release in culture supernatants of LPS-primed (4 h) primary *Wnk1*^*+/+*^ LysMCre^*+*^ (WNK1 WT) and *Wnk1*^*flox/flox*^ LysMCre^*+*^ (WNK1 KO) BMDMs in Cl^−^ free (**e**) or K^+^ free (**f**) media for the indicated time points. *P* values of **e** are 0.07975, 0.15334, 0.01091, 0.00002, and 0.00126; **f** are 0.8922, 0.01337, 0.00036, 0.00000008, and 0.000062. Results are representative of at least three independent experiments performed in duplicate or triplicate. Error bars in **a**–**f** are presented as mean values ± standard deviation (S.D.), with *n* = 3. Two-sided Student’s *t* test, *p < 0.05, **p < 0.005, ***p < 0.0005.

To investigate whether K^+^ or Cl^−^ efflux drives activation of NLRP3 in WNK1 deficient BMDMs, we tested whether incubation of BMDMs in a Cl^−^-free or K^+^-free medium can activate the NLRP3 inflammasome without an exogenous NLRP3 stimulus (signal 2). Incubation in a Cl^−^ -free medium resulted in a rapid decline in intracellular Cl^−^ in both WT, and to a larger extent, in WNK1-KO cells (Fig. 7a). However, there was very little or no change in the level of intracellular K^+^ in the first 50 min of incubation in both the WT and WNK1-KO cells (Fig. 7b). Nevertheless, the level of intracellular K^+^ declined modestly only in WNK1-KO cells at later time points (Fig. 7b). Incubation in a K^+^-free medium resulted in a small decline in intracellular Cl^−^ and a modest decline in K^+^ in WT cells, but a greater decline in both intracellular Cl^−^ and K^+^ was observed in WNK1-KO cells (Fig. 7c, d). For WT cells in Cl^−^ free medium, the decline in intracellular Cl^−^ in the absence of a similar decline in intracellular K^+^ (Fig. 7a, b) did not activate the NLRP3 inflammasome as measured by IL-1β secretion (Fig. 7e). In contrast, a decline in intracellular Cl^−^ and an eventual small decline in intracellular K^+^ levels in WNK1-KO cells in Cl^−^ free media (Fig. 7a, b) resulted in NLRP3 activation (Fig. 7e). A decline in both K^+^ and Cl^−^ levels in K^+^-free media activated the NLRP3 inflammasome in both WT and WNK1-KO cells, but NLRP3 activation was much greater in WNK1-KO cells (Fig. 7c, d, f). These results suggest that both Cl^−^ and K^+^ effluxes drive NLRP3 activation, and these fluxes are regulated by WNK1.

Although imiquimod-induced NLRP3 activation is believed to be independent of K^+^ efflux^48^, our results showing enhancement of imiquimod-induced NLRP3 activation by deletion or inhibition of WNK1 suggest that Cl^−^ or K^+^ efflux or both play an important role in the imiquimod-induced NLRP3 activation mechanism. Supporting this, imiquimod stimulated K^+^ and Cl^−^ efflux in WNK1-KO BMDMs (Fig. 6c, f). Furthermore, excess extracellular Cl^−^ or K^+^, but not sodium, inhibited the decline in intracellular K^+^ levels (Supplementary Fig. 17), and blocked NLRP3 activation and IL-1β generation (Supplementary Figs. 18 and 19) in both WT and WNK1 KO cells in response to ATP or imiquimod.

In response to hypotonic stress, cells undergo regulatory volume decrease (RVD) by activating ion channels and transporters, which cause effluxes of K^+^, Cl^−^, and H_2_O, leading to cell shrinkage^54^. As WNK1 has been reported to be activated by hypotonic stress^55^ and because hypotonicity-induced RVD has also been shown to activate NLRP3^56^, we tested whether WNK1 deficiency affects NLRP3 activation in response to hypotonic stress. Our results show that incubation in a hypotonic medium induces a greater increase in NLRP3 inflammasome activation in WNK1-KO compared with WT BMDMs, as measured by caspase-1 activation, IL-1β secretion, PI uptake and LDH release (Supplementary Fig. 20). These results indicate that WNK1 plays a critical role in suppressing hypotonicity-induced NLRP3 activation.

### WNK1 regulates innate immune responses in vivo

**Fig. 8:**
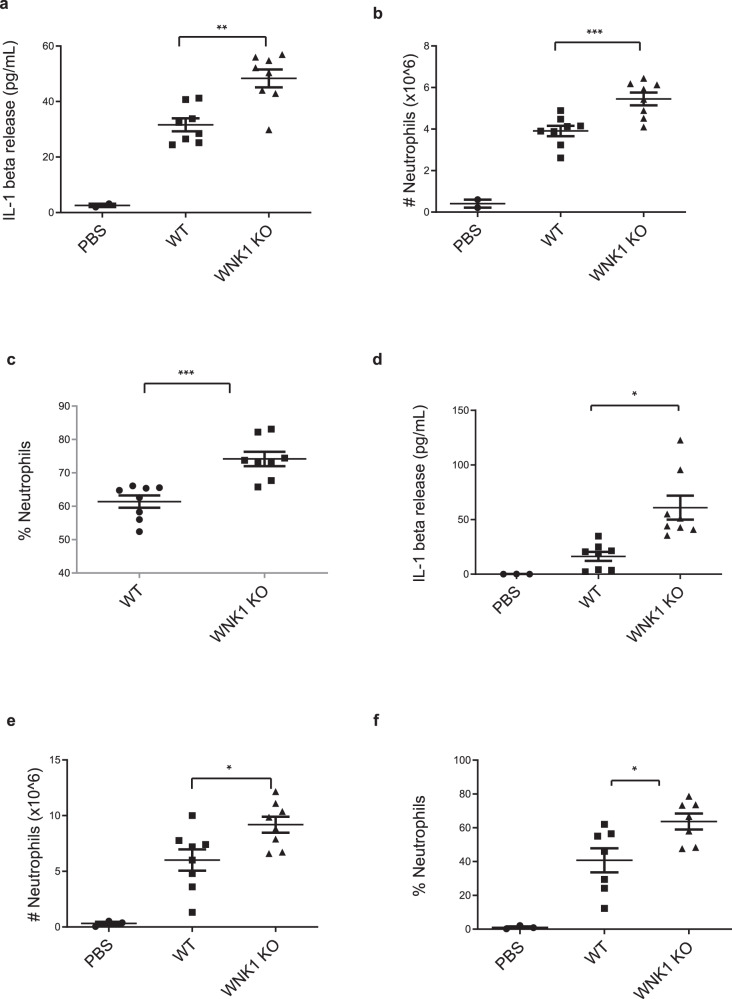
WNK1 knockout in macrophages increases NLRP3 inflammasome activation in vivo. IL-1β release (**a**), number of infiltrating neutrophils (**b**), and percentage of neutrophils (**c**) were measured from peritoneal exudate of *Wnk1*^*+/+*^ LysMCre^*+*^ (WNK1 WT) and *Wnk1*^*flox/flox*^ LysMCre^*+*^ (WNK1 KO) mice injected with 1 mg MSU for 6 h; *n* = 8 mice per group. *P* value for **a** is 0.002018; **b** is 0.00009; **c** is 0.00012. IL-1β release (**d**), number of infiltrating neutrophils (**e**), and percentage of neutrophils (**f**) were measured from peritoneal exudate of *Wnk1*^*+/+*^ LysMCre^*+*^ (WNK1 WT) and *Wnk1*^*flox/flox*^ LysMCre^*+*^ (WNK1 KO) mice injected with 1 mg MSU for 16 h; *n* = 8 mice per group. *P* value for **d** is 0.01299; **e** is 0.04810; **f** is 0.04467. 3 mice per experiment were injected with PBS as a negative control. Error bars in **a**–**f** are presented as mean values ± standard deviation (S.D.). Two-sided Student’s *t* test, *p < 0.05, **p < 0.005, ***p < 0.0005.

To provide physiological relevance to our in vitro findings, we investigated the role of WNK1 in NLRP3-dependent innate immune response in vivo. Intraperitoneal injection of monosodium urate (MSU) crystals initiates a NLRP3-dependent immune response, characterized by neutrophil infiltration and IL-1β release into the peritoneum^51,57^. We injected 8–12-week-old *Wnk1*^*flox/flox*^ LysMCre^*+*^ mice and *Wnk1*^*+/+*^ LysMCre^*+*^ littermates with 1 mg MSU for a 6 h “short” activation or a 16 h “long” activation of NLRP3, before collecting and analyzing peritoneal exudate. We found that after 6 h of MSU treatment, conditional WNK1 KO mice had significantly higher levels of IL-1β in their peritoneal exudate compared to wild type littermates (Fig. 8a). FACs analysis of exudate revealed an increase in the number of infiltrating neutrophils (Fig. 8b) as well as an increase in the percent population of neutrophils (Fig. 8c) in the conditional WNK1 KO mice. After 16 h of MSU treatment, we observed an increase in IL-1β release (Fig. 8d), number of infiltrating neutrophils (Fig. 8e), and percent neutrophils in the exudate population (Fig. 8f) in the conditional WNK1 KO mice compared to their wild type littermates. We did not observe significant differences in TNFα levels after either the short or long MSU treatment (Supplementary Fig. 21). Together, these results indicated that WNK1 plays an important role in NLRP3 activation in vivo.

### WNK4 does not regulate NLRP3 activation

**Fig. 9:**
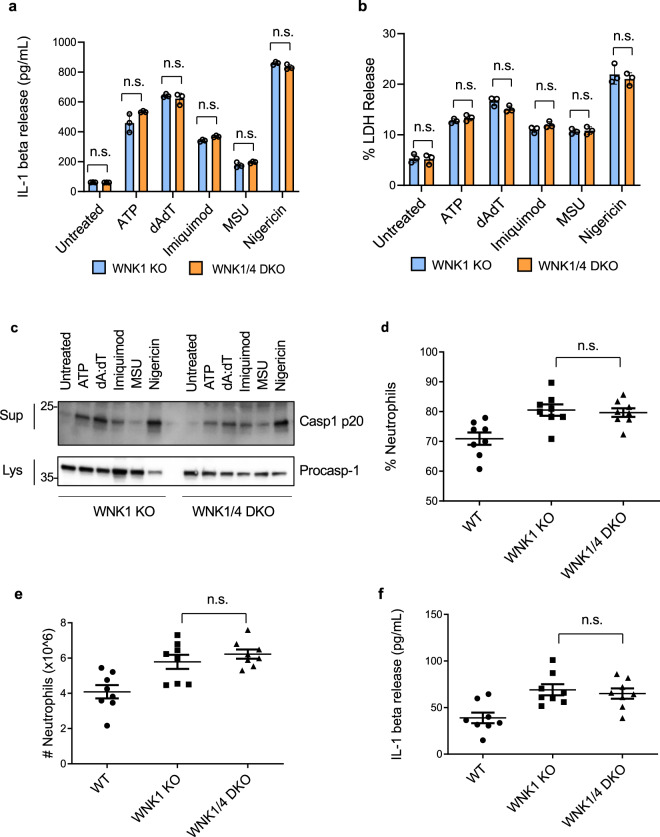
WNK4 knockout does not increase NLRP3 inflammasome activation in vitro or in vivo. IL-1β (**a**) and LDH (**b**) release of culture supernatant of LPS-primed (4 h) primary *Wnk1*^*flox/flox*^ LysMCre^*+*^ (WNK1 KO) and *Wnk1*^*flox/flox*^ LysMCre^*+*^ *Wnk4*^*−/−*^ BMDMs (WNK1/4/ DKO) treated with ATP, dAdT transfection, imiquimod, MSU, and nigericin as indicated. *P* values in **a** are 0.3684, 0.1465, 0.5583, 0.0642, 0.1596, and 0.2239; **b** are 0.8798, 0.3554, 0.0879, 0.1037, 0.8199, and 0.2771. **c** Immunoblots of caspase-1 p20 released in culture supernatants (Sup) and procaspase-1 in cell lysates (Lys) of LPS-primed (4 h) primary *Wnk1*^*flox/flox*^ LysMCre^*+*^ (WNK1 KO) and *Wnk1*^*flox/flox*^ LysMCre^*+*^ *Wnk4*^*−/−*^ BMDMs (WNK1/4/ DKO) treated with ATP, dAdT transfection, imiquimod, MSU, and nigericin as indicated. Percentage of neutrophils (**d**), number of infiltrating neutrophils (**e**), and IL-1β release (**f**) were measured from peritoneal exudate of *Wnk1*^*flox/flox*^ LysMCre^*+*^ (WNK1 KO) and *Wnk1*^*flox/flox*^ LysMCre^*+*^ *Wnk4*^*−/−*^ BMDMs (WNK1/4/ DKO) mice injected with 1 mg MSU for 6 h; *n* = 8 mice per group. *n* = 8 mice per group. *P* value **d** is 0.5569, **e** is 0.5483, and **f** is 0.4089. 3 mice per experiment were injected with PBS as a negative control. Error bars in **a**, **b**, **d**–**f** are presented as mean values ± standard deviation (S.D.), with *n* = 3. Two-sided Student’s *t* test, n.s. not significant.

WNK1 and WNK4 are both Cl^−^ sensitive kinases^53,58,59^, so we investigated if WNK4 enhances inflammasome activation like WNK1, or if WNK4 plays a compensatory role for WNK1, and a WNK1/WNK4 double knockout would have even more activation. We did not observe an increase in NLRP3 activation in WNK4 KO BMDMs compared to wild type (Supplementary Fig. 22). Since *Wnk4* knockout is not lethal in mice, we were able to develop a *Wnk1*^*flox/flox*^ LysMCre^*+*^ *Wnk4*^*−/−*^ mouse (referred to as WNK1/4 DKO mouse). We did not observe an additive effect on NLRP3 activation in vitro when comparing conditional WNK1 KO BMDMs to WNK1/4 DKO BMDMs as measured by IL-1β release (Fig. 9a), LDH release (Fig. 9b), or caspase 1 activation (Fig. 9c) when stimulated with a variety of inflammasome stimuli. We also did not see an observable difference between WNK1 KO and WNK1/4 DKO in vivo when measuring immune response after MSU injection as measured by percent neutrophils in the exudate (Fig. 9d), number of infiltrating neutrophils to the peritoneum (Fig. 9e), or IL-1β release (Fig. 9f). These results show WNK1 but not WNK4 is critical in regulating NLRP3 activation and immune response. In addition, WNK4 activity does not substitute for a loss of WNK1, as the WNK1/4 DKO showed no significant effect compared to the conditional WNK1 KO.

## Discussion

**Fig. 10:**
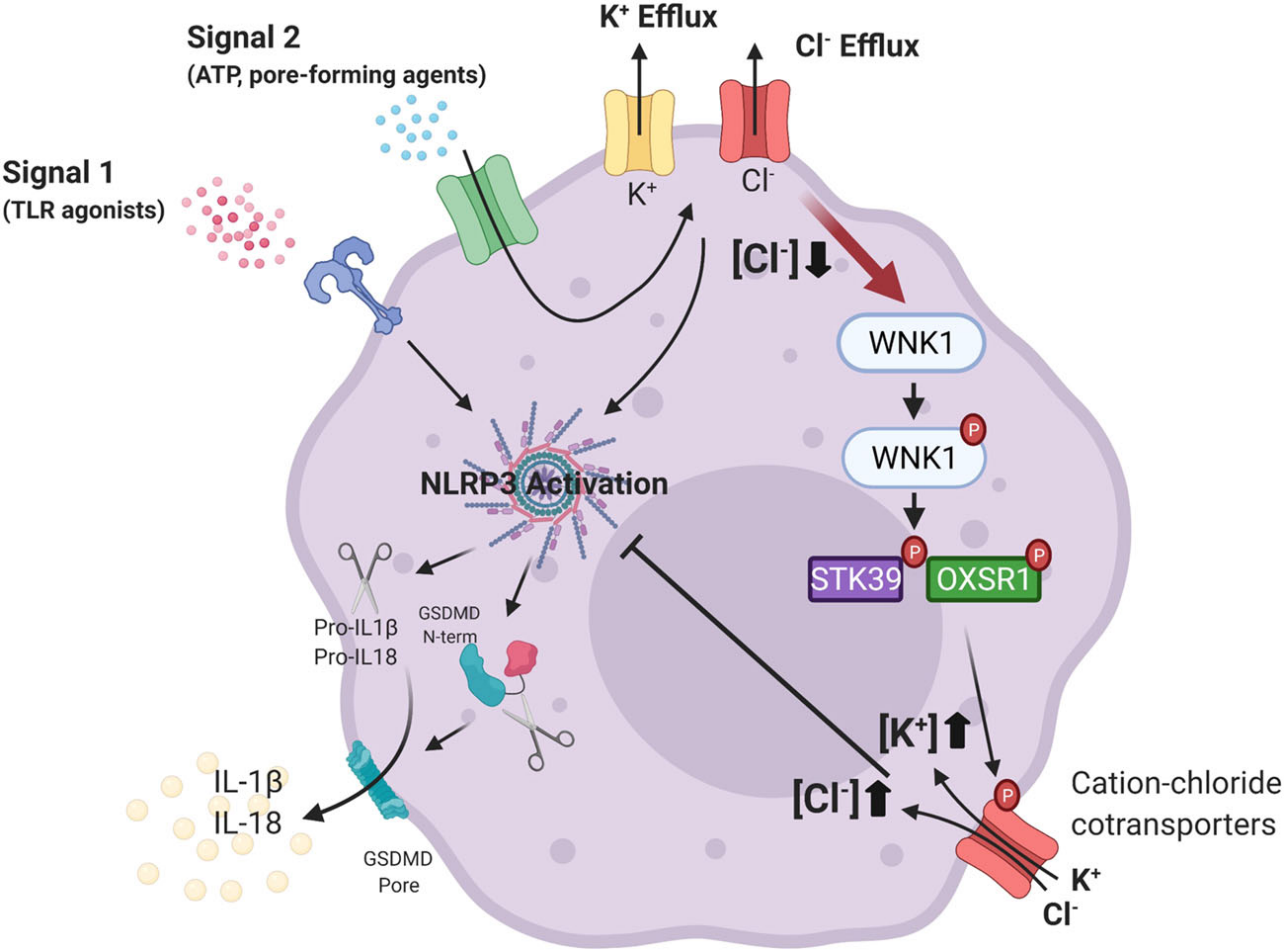
Model for regulation of NLRP3 activation and pyroptosis by WNK1 signaling. Following the NLRP3 inflammasome priming step (signal 1), NLRP3 activating stimuli (signal 2) induce K^+^ and Cl^−^ efflux from the cell which trigger NLRP3 inflammasome assembly. WNK1 senses the resulting decrease in intracellular Cl^−^ and becomes autophosphorylated. WNK1 proceeds to phosphorylate its downstream targets STK39/OXSR1, which activate cation-Cl^−^ cotransporters to restore ion concentrations as well as inhibit further Cl^−^ efflux. Restoration of normal intracellular Cl^−^ concentration inhibits further NLRP3 inflammasome activation and pyroptosis. (Created with www.BioRender.com).

The NLRP3 inflammasome is a powerful player in innate immunity to fight off invading pathogens and damage, but excess inflammation caused by dysregulated NLRP3 activation is attributed to a wide variety of diseases such as inflammatory bowel diseases, atherosclerosis, rheumatoid arthritis, gout, type 2 diabetes, and Alzheimer’s disease^21^. Thus, understanding NLRP3’s regulation is critical to develop treatment strategies to counter this excess inflammation. Our study has identified WNK1 as a critical regulator of NLRP3’s activation due to its ability to control intracellular Cl^−^ and K^+^ levels (Fig. 10).

Recent studies have uncovered Cl^−^ efflux as a critical step in NLRP3’s activation and have also implicated CLIC Cl^−^ channels in this process^16,38,40^. One study demonstrated that translocation of CLICs to the plasma membrane and CLICs-dependent Cl^−^ efflux in response to NLRP3 stimuli is an essential signaling event upstream of NLRP3 activation^38^. Our study provides further support for the importance of Cl^−^ efflux in NLRP3 activation by demonstrating that the Cl^−^ sensor WNK1 is a critical negative regulator of Cl^−^ efflux and NLRP3 activation in macrophages. BMDMs lacking WNK1 show increased Cl^−^ efflux and NLRP3 activation. In WT BMDMs, upon NLRP3 agonist, Cl^−^ is rapidly released from the cell, but homeostasis can be restored within 15–20 m of treatment. Without WNK1, BMDMs have a larger decline in Cl^−^ concentration upon NLRP3 agonist addition, and the cell is not able to prevent the loss of intracellular Cl^−^. Since intracellular Cl^−^ levels are regulated in part by WNK1’s action on cation-Cl^−^ cotransporters, which also regulate intracellular K^+^, any decline in intracellular Cl^−^ levels may also affect intracellular K^+^ levels and vice versa. Our results show that the loss of intracellular Cl^−^ leads to a greater loss in intracellular K^+^ in WNK1-KO cells resulting in stronger NLRP3 inflammasome activation by exogenous NLRP3 stimuli. Intriguingly, the loss of intracellular Cl^−^ when WNK-KO BMDMs are incubated in a K^+^-free medium leads to a greater loss of intracellular K^+^, resulting in the activation of the NLRP3 inflammasome without an exogenous signal 2. This underscores the importance of Cl^−^ sensing by WNK1, which not only impacts intracellular Cl^−^ but also K^+^ levels. This may also have important clinical consequences as one of the side effects of loop and thiazide diuretics is hypokalemia^60–62^. Since loop and thiazide diuretics inhibit downstream WNK1 signaling events by blocking cation-Cl^−^ co-transporters, it is likely that these two events play important roles in the severity of gout in patients taking diuretics.

It is currently unclear whether K^+^ or Cl^−^ efflux is the driving force for NLRP3 activation. Our data demonstrate that only when both intracellular Cl^−^ and K^+^ decline simultaneously, as a result of incubation in K^+^ or Cl^−^ -free media or stimulation with NLRP3 stimuli, there is strong activation of the NLRP3 inflammasome. This indicates that the combined loss of intracellular K^+^ and Cl^−^ is what drives NLRP3 activation. Supporting this, stimulation of both WT and WNK1-KO with NLRP3 stimuli in the presence of excess extracellular Cl^−^ or K^+^, but not sodium, is sufficient to block K^+^ efflux and NLRP3 activation.

Mutations in WNK1 cause an increase in WNK1 expression and gain of function activity, resulting in an autosomal dominant form of hypertension called Pseudohypoaldosteronism type II (PHAII)^63^. PHAII is dependent on Cl^−^ and is effectively treated by thiazide diuretics to inhibit excess WNK1 activity^63,64^. In addition to PHAII, patients with so-called “essential” hypertension, which represent up to 95% of hypertension cases, also respond to thiazide diuretics, suggesting variants of WNK1 may be more present in the general population than we realize^63^. Recently, WNK463 was tested in rats as a potential therapeutic for hypertension. While rats treated with this WNK inhibitor had positive effects of lowering blood pressure, the study was discontinued due to unspecified effects^44^. Interestingly, we observed that inhibition of cation-Cl^−^ cotransporter activity via WNK inhibitors, OXSR1/ STK39 inhibitors or thiazide diuretics causes a similar increase in NLRP3 activation. This suggests an important link between treating blood pressure by targeting the WNK1 pathway and increasing the risk of exaggerating NLRP3-mediated inflammatory diseases such as gout. In support of this is the known phenomenon that treatment with thiazides can induce gout, which is triggered by NLRP3 activation^45,51,65^. Although the mechanism by which diuretics induce gout is not fully understood, we speculate that the pathogenesis of gout following treatment with a thiazide diuretic is occurring in a two-step process. The first step is the well-characterized buildup of urate crystals in the body due to their overproduction and underexcretion^51,52^. The build-up of urate crystals provides the initial stimulus for NLRP3 activation^51^. In the second step the inhibition of Na^+^/Cl^−^ co-transporter NCC by the thiazide diuretic potentiates activation of the NLRP3 inflammasome by the urate crystals. As mentioned above, thiazide and loop diuretics are known to induce hypokalemia in vivo^60–62^, which according to our findings can also enhance NLRP3 activation. Perhaps we do not see a similar effect with furosemide on macrophages in vitro (Supplementary Fig. 12), because NCC and KCC cotransporters may compensate for the inhibition of NKCC1. It is also possible that furosemide might be less effective in inhibiting macrophage NKCC1, as NKCC1 exhibits different sensitivity to furosemide in different cell types^66^. Taken together, although diuretics are in many cases successfully given to people to treat high blood pressure, their ability to inhibit the WNK1 signaling pathway at the level of the Na^+^/K^+^/Cl^−^ co-transporters and their effect on blood K^+^ levels may increase the severity of NLRP3-related inflammatory complications such as gout.

Although WNK4 is more sensitive to Cl^−^ levels than WNK1^67^, we observed that WNK1 has an effect on NLRP3 activation but WNK4 does not. This could be explained by the possibility that WNK4 might not be expressed at functional levels in macrophages or the fact that WNK1’s activities are also regulated by cell volume, a known regulator of NLRP3, but WNK4 is not sensitive to volume changes^56,67^. Interestingly, a recent study found that WNK3 is sensitive to changes in cell volume^67^. WNK3 functions in promoting regulatory volume increase (RVI) and in turn inhibiting regulatory volume decrease (RVD), so it is possible that WNK3 may instead work in connection with WNK1 as a regulator of NLRP3 activation if WNK3 is present in macrophages.

Overall, our study identifies a previously unknown regulatory step involving WNK1 that controls NLRP3 activation and pyroptosis in macrophages. This study reinforces the importance of Cl^−^ regulation in NLRP3 inflammasome activation, which has recently gained interest in the field. By sensing low intracellular Cl^−^ levels in macrophages, WNK1 balances intracellular ions (Na^+^, K^+^, and Cl^−^) via activation of the cation-Cl^−^ cotransporters, which dampens NLRP3 inflammasome activation, thus preventing excess inflammation. Importantly, we also demonstrate for the first time that although the small molecule imiquimod is believed to activate NLRP3 by a K^+^-independent mechanism, its activation of the NLRP3 inflammasome is sensitive to WNK1 deletion or inhibition suggesting that ion (K^+^ and Cl^−^) homeostasis plays a critical role in imiquimod-induced NLRP3 activation. Finally, our study suggests clinical implications of inhibiting the WNK1 pathway, including the use of diuretics to treat high blood pressure, as inflammatory complications linked to NLRP3 regulation by the WNK1 pathway may arise.

## Methods

### Antibodies and reagents

Rabbit polyclonal antibodies against NLRP3 (immunogen: human amino acids 1–198) and caspase-1 (immunogen: mouse caspase 1 p20 subunit) were generated through Invitrogen. For validation, recombinant proteins were expressed in bacteria, affinity purified, and verified on Coomassie-stained PAGE gels. Titers >2048000 were obtained for both antibodies. NLRP3 antibody was used at 1:2000 dilution and caspase-1 antibody was used at 1:1500 dilution. Anti-mouse ASC antibody was a gift from Dr. Junji Sagara and used at 1:2000 dilution. Anti-IL-1β was from GeneTex (Catalog No. GTX74034) and used at 1:10,000 dilution. Anti-WNK1 (2360–2382) [Sheep No. S062B] antibody was obtained from MRC PPU Reagents and was used at a 1:250 dilution. For in vivo experiments, Alexa Fluor 594 anti-mouse CD3ε (BioLegend Cat No. 152317), Ly-6B.2 FITC (Biorad Ref MCA771FT), Anti-mouse Ly6G APC (Tonbo Ref 20–1276-UO25), and Anti-mouse CD45 violetFluor 450 (Tonbo Ref 75–0451-UO25) were used at 1:200 dilution each.

Lipopolysaccharide (LPS) (Cat No. L8274), Adenosine 5’-triphosphate disodium salt hydrate (ATP) (Cat No. A2383), propidium iodide (Cat No. P4170), Furosemide (Cat No. F4381), Hydrochlorothiazide (Cat No. H4749), N-acetyl cysteine (A7250), Closantel (Cat No. 34053), Rafoxanide (Cat No. 34042), and 1G244 (Cat No. SML2247) were obtained from Sigma. WNK463 inhibitor (Cat No. HY-100626) and WNK-IN-11 WNK1 inhibitor (Cat No. HY-112094) were obtained from MedChem Express. Mono-sodium urate crystals (Cat No. tlrl-msu), Poly(dA:dT) (Cat No. tlrl-patn), and nigericin (Cat No. tlrl-nig) were obtained from Invivogen. CytoTox96 LDH release kit (Cat No. G1780) was from Promega. (Z)−4 Hydroxytamoxifen (Cat No. ALX-550-361-M005) was obtained from Enzo Life Sciences. Imiquimod (Cat No. 3700) was obtained from Tocris. MQAE (N-(Ethoxycarbonylmethyl)−6-Methoxyquinolinium Bromide) (Cat No. E3101) was obtained from Invitrogen/Thermo Scientific.

### Cell culture and treatments

Primary bone marrow-derived cells were harvested from the femurs of wildtype (C57BL/6) (Jackson) and *WNK1/*LysM-Cre, *WNK4* knockout^68^, and *NLRP3*^51^ knockout mice and differentiated into BMDMs by culturing in Dulbecco’s modified Eagle’s medium (DMEM; Gibco) supplemented with 10% FBS, 10 mM HEPES pH 7.0 (Invitrogen), 100 U mL^−^ penicillin and streptomycin (complete DMEM), and 20% L929 supernatants in 10 cm dishes at 37 °C with 5% CO_2_ for 5–6 days.

Immortalized BMDMs were generated by transformation of primary BMDMs with J2-CRE retrovirus^7^. Immortalized caspase 1 KO macrophages were derived from a *Caspase 1−/−* mouse and NLRP3 KO macrophages were derived from a *NLRP3−/−* mouse^51^. Rip1 wildtype, Rip1 KO, and ASC KO macrophages were gifts from Dr. Kate Fitzgerald. Immortalized macrophages were maintained in Dulbecco’s modified Eagle’s medium (DMEM; Gibco) supplemented with 8% FBS, 10 mM HEPES pH 7.0 (Invitrogen), and 100U mL^−^ penicillin and streptomycin. Bone marrow was also isolated from mice carrying a floxed *Wnk1* gene and a tamoxifen-inducible CreERT2 expressed from the *ROSA26* locus (*Wnk1*^fl/fl^ CreERT2^+^) described in^35^. After differentiation in L929-supplemented medium, the differentiated BMDMs were immortalized by transformation with J2-CRE retrovirus. In some cases, the differentiated primary BMDMs were treated with 0.02 mg/mL 4 Hydroxytamoxifen (4HT) in supplemented DMEM for 48 h to allow the Cre recombinase to delete *Wnk1*. After 48 h, the *Wnk1*-deleted cells were used for experiments as indicated.

For the various treatments, BMDMs were seeded in six-well plates at a density of 1 × 10^6^ cells per well overnight. The next day BMDMs were pre-stimulated with the TLR ligands ultrapure LPS (500 ng ml^−1^) or Pam3CSK4 (1 μg ml^−1^) for 4 h followed by stimulation with ATP (5 mM), Nigericin (10 µM), imiquimod (70 µM), MSU (5 mg/mL), or transfected with poly(dA:dT) (1 μg ml^−1^) using Lipofectamine 2000 (7 μl ml^−1^) as per the manufacturer’s protocol (Invitrogen) in OPTI-MEM media for various times as indicated.

For measurement of propidium iodide (PI) uptake, cells are stained with 1.5 µM propidium iodide (Sigma- Cat No. P4170) and at the start of treatment, plates were placed in the IncuCyte® S3 Live Cell Analysis System and imaged at 20× magnification. Signal percentage was calculated with the formula (percent red confluence/percent phase confluence) × 100. Unless otherwise indicated, background signal from untreated samples was subtracted from treated sample signal.

### Measurement of NLRP3 oligomerization in GFP-tagged NLRP3 macrophages

Immortalized murine NLRP3 knockout macrophages derived from *NLRP3* knockout mice and reconstituted with GFP-tagged NLRP3 (C-terminus) were primed with LPS for 10 m followed by treatment with 30 µM of the pan-caspase inhibitor Emricasan (Sigma- Cat No. SML2227) for 10 m to allow for visualization of NLRP3 oligomerization without caspase 1 activation and pyroptosis. Macrophages were next incubated with DMSO or 1 µM WNK-IN-11 prior to treatment with ATP or Nigericin. Phase and fluorescent images of the cells were taken at 20× magnification every 5 min for 1 h on the IncuCyte® S3 Live Cell Analysis System, and the number of NLRP3-GFP oligomer specks were counted and normalized to cell count by the IncuCyte® S3.

### LDH release assay

Pyroptosis was quantitated by assaying the activity of LDH released into cell culture supernatants after various treatments using the CytoTox96 LDH release kit (Promega- Cat No. G1781) according to the manufacturer’s protocol. The LDH activity in the culture supernatant was expressed as a percentage of total LDH in the cell lysate.

### IL-1β release ELISA

IL-1β released into cell culture supernatants after various treatments was quantitated using the Mouse IL-1 beta/IL-1F2 Quantikine ELISA Kit (R&D- Cat No. MLB00C) according to the manufacturer’s protocol. The standard curve was generated using the kit’s standards.

### Immunoblot analysis

BMDMs were lysed in buffer containing 50 mM Tris, pH 7.5, 150 mM NaCl, 1 mM EDTA, 0.1% NP-40, and protease inhibitors and clarified by spinning at 20,000 × *g* for 10 min at 4 °C in an Eppendorf tabletop 5417 R refrigerated microcentrifuge. Cell lysates were fractionated by SDS–PAGE and then transferred to polyvinylidene difluoride (PVDF) membranes (Bio-Rad).

To detect WNK1, cell lysates were run on a 4–20% gradient gel (Bio-Rad) for 1 h. The fractionated proteins were then transferred to PVDF membrane (Bio-Rad) at 20 V for 16 h at 4 °C.

To examine caspase-1 p20 and IL-1β release, cell culture supernatants were precipitated by methanol/chloroform method^7^. Briefly, culture media were spun at 425 × *g* for 5 min to pellet cells and cell debris. Supernatants were transferred to a fresh tube and proteins were precipitated by the addition of an equal volume of methanol and 0.25 volumes of chloroform to each sample followed by centrifugation at 15,000 × *g* for 10 min. The upper phase was discarded and the same volume of methanol was again added to the interphase of each sample followed by centrifugation for 5 min at 20,000 × *g*. The resulting protein pellets were dried at room temperature, resuspended in 2× Laemmli buffer, and boiled for 10 min at 98 °C until dissolved. The resuspended proteins were fractionated on 12% SDS–PAGE followed by electroblotting onto PVDF membranes. Blots were probed with appropriate antibodies.

### ASC oligomerization assay

After stimulation of cells in OPTI-MEMI in six-well plates, the culture supernatants were collected and used for immunoblot analyses of secreted caspase-1 p20 as described above. Cells were lysed in 0.5 ml buffer containing 20 mM Hepes-KOH, pH7.5, 150 mM KCl, 1% NP40, 0.1 mM phenylmethylsulphonyl fluoride and protease inhibitor cocktail on ice. The cell lysates were centrifuged at 3825 × *g* at 4 °C for 10 min. The NP40-soluble supernatants were removed and the NP40-insloluble pellets were washed 1× in the lysis buffer and then resuspended in 50 μl of the same buffer. The resuspended pellets were cross-linked with fresh disuccinimidyl suberate (2 mM) for 30 min at room temperature and then immediately mixed with 50 μl 2 × SDS sample buffer, boiled and fractionated on 12% SDS–polyacrylamide gel followed by immunoblotting with anti-mouse ASC antibody.

### Generation of CRISPR/Cas9 knockout cell lines

The CRISPR design tools at MIT (http://crispr.mit.edu) or Benchling (https://benchling.com) were used to identify candidate single-guide RNA (sgRNA) sequences. Sequences targeting murine *Wnk1* exon 1 (5’ CCGAGAAGCAAAGCGGCACTC 3’) and (5’ TACAACGGCTTGTTCCCCAG 3’) (also listed in Supplementary Table 1) were cloned into lentiCRISPRv2GFP vector (Addgene) containing Cas9 fused to EGFP. Next, 5 µg lentiCRISPRv2GFP sgRNA plasmids were cotransfected with 3.75 µg psPAX2 and 2.5 µg VSVg plasmids (Addgene) using 25 µL Lipofectamine 2000 to 2 × 10^6^ 293Ts seeded the day before. At 72 h after transfection, media were collected and concentrated overnight using Lenti-X Concentrator (Takara Cat. No. 631232) as per the manufacturer’s protocol. Infected 1 × 10^6^ cells mL^−^ macrophages were plated in a 12-well plate with concentrated lentivirus. Cells were then enriched for Cas9-EGFP expression by flow cytometry and single cells were then isolated and screened by western blot analysis for protein expression. Sequencing was performed on PCR-amplified genomic DNA encompassing the sgRNA targeting sequences.

### Chloride replacement experiments

Adapted from Green et al.^16^. Primary BMDMs were primed with LPS (500 ng ml^−1^) for 4 h in supplemented DMEM. Following priming, the cells’ media was changed to the indicated isotonic salt solution: control (145 mM NaCl,5 mM KCl) or Cl¯ free (145 mM NaGluconate,5 mM KGluconate). When used, WNK1 inhibitors were added to the cells 10 m prior to the isotonic solutions and inhibitor was added to the isotonic solutions as well. Cells were then stimulated with nigericin (10 µM) for the indicated times.

### Measurement of intracellular chloride

Adapted from Tang et al.^38^, primary macrophages were treated as indicated in 12-well plates in DMEM containing 20% L929 conditioned medium. Following treatments, supernatant was removed and 200 µL of cell culture grade water was added to each well and plates were incubated for 15 m at 37 °C for lysis. The lysates were then transferred to 1.5 mL Eppendorf tubes and centrifuged at 10,000 × *g* for 5 m. 50 µL of lysate was then mixed with 50 µL MQAE (10 µM) and added to a black 96-well plate. The fluorescence intensity was measured at Excitation 350 nm and Emission 460 nm using the BMG Labtech CLARIOstar^*Plus*^ reader.

### Measurement of intracellular potassium

Primary macrophages were loaded with 5 μM ION Potassium Green-2 AM, K^+^ indicator (Abcam Cat No. 142806) in PBS containing 0.1% Pluronic F-127 (Thermo Cat No. P3000MP) and incubated at 37 °C for 30 m. Following incubation, the loading solution was removed and cells were washed 2× with regular PBS. Macrophages were then treated as indicated and green fluorescence intensity was measured on the IncuCyte® S3 Live Cell Analysis System at 10× magnification. Fluorescence intensity of each sample was normalized to the untreated intensity.

### Mice

Mice were all backcrossed (N6) and maintained on a C57BL/6 background. Mouse strains were maintained in specific pathogen-free conditions at 68–72° Fahrenheit and 30–70% humidity on a 6 pm/6am nocturnal dark/light cycle. The animal protocols were carried out in accordance with the guidelines set forth by the Thomas Jefferson University Institutional Animal Care and Use Committee. The authors complied with all relevant ethical regulations for animal testing and research. All experiments performed in this study received previous ethical approval and study protocols were approved by the Thomas Jefferson University Institutional Animal Care and Use Committee. Both male and female mice (age 2–6 months) were used for harvesting bone marrow-derived macrophages and for in vivo experiments.

### Generation of *Wnk1/*LysMCre mice

8-week-old homozygous LysMCre^*+*^ mice (ATCC stock #004781 B6.129P2-Lyz2^*tm1(cre)/fo*^/J) were mated with 8-week-old homozygous *Wnk1*^*flox/flox*^ mice^47,69,70^ to produce double heterozygous *Wnk1*^*+/flox*^ LysMCre^*+*^ mice. Double heterozygous mice were mated, resulting in *Wnk1*^*+/+*^ LysMCre^*+*^ and *Wnk1*^*flox/flox*^ LysMCre^*+*^ siblings, which were used for generating the study’s BMDMs. *Wnk1*^*+/+*^ LysMCre^*+*^ and *Wnk1*^*flox/flox*^ LysMCre^*+*^ cohorts were maintained and used for in vivo experiments.

### Generation of *Wnk1/*LysMCre*/Wnk4* mice

8-week-old *Wnk4*^−/−^ mice^68^ were mated with 8-week-old *Wnk1*^*flox/flox*^ LysMCre^*+*^ mice to generate triple heterozygous *Wnk1/*LysMCre*/Wnk4* mice. Triple heterozygous mice were mated, resulting in *Wnk1*^*flox/flox*^ LysMCre^*+*^ *Wnk4* and *Wnk1*^*f*lox/flox^ LysMCre^*+*^ *Wnk4*^*−/−*^ siblings, which were used for generating the study’s BMDMs. *Wnk1*^*flox/flox*^ LysMCre^*+*^ *Wnk4*^*+/+*^ and *Wnk1*^*flox/flox*^ LysMCre^*+*^ *Wnk4*^*−/−*^ cohorts were maintained and used for in vivo experiments.

### MSU-induced peritonitis

Protocol was adapted from bio-protocol’s “Mono Sodium Urate Crystal-induced Peritonitis for in vivo Assessment of Inflammasome Activation”^57^. In brief, adult mice were injected intra-peritoneally with 1 mg MSU crystals resuspended in 0.2 mL PBS. After 6 h or 16 h, mice are euthanized and injected with 4 mL PBS into the peritoneum. Lavage is collected and 1 × 10^6^ cells are stained for flow cytometry analysis as described below and supernatant is assayed for IL-1β using the ELISA kit mentioned above. IL-1β results were normalized to the number of cells collected in the corresponding lavage sample. TNFα was similarly assayed using Mouse TNF-alpha Quantikine ELISA Kit (R&D- Cat No. MTA00B) according to the manufacturer’s protocol. The standard curve was generated using the kit’s standards.

### Flow cytometry

Cells (1 × 10^6^) collected from the lavage were stained at 1:200 dilution of each of the following antibodies: Alexa Fluor 594 anti-mouse CD3ε (BioLegend Cat No. 152317), Ly-6B.2 FITC (Biorad Ref MCA771FT), Anti-mouse Ly6G APC (Tonbo Ref 20–1276-UO25), and Anti-mouse CD45 violetFluor 450 (Tonbo Ref 75–0451-UO25). Samples were fixed with BD Cytofix fixation buffer (BD Biosciences Cat No. 554655) before sorting on the BD LSRFortessa (BD) under the guidance of the SKCC Flow Cytometry and Cell Sorting Facility, Thomas Jefferson University, Philadelphia, PA. Analysis was performed using FlowJo software version 10. The gating strategy used to determine the neutrophil population is shown in Supplementary Fig. 23.

### Statistics

Statistical analyses were made with two-sided Student’s *t* test. For all experiments, *P < 0.05, **P < 0.01, and ***P < 0.001.

### Reporting summary

Further information on research design is available in the Nature Research Reporting Summary linked to this article.

## Supplementary information

Supplementary Information

Reporting Summary

## Data Availability

The data that support the findings of this study are available within the article and supplementary files including a Source Data file, or are available from the corresponding author upon request. Source data are provided with this paper.

## Source data

Source Data
