# Supplementary material for: Chloride sensing by WNK1 regulates NLRP3 inflammasome activation and pyroptosis
Source: Nat Commun. 2021 Jul 27;12:4546. doi: 10.1038/s41467-021-24784-4 (PMC8316491; doi:10.1038/s41467-021-24784-4)
Supplement: Supplementary file 1 — Supplementary Information [file 41467_2021_24784_MOESM1_ESM.pdf]

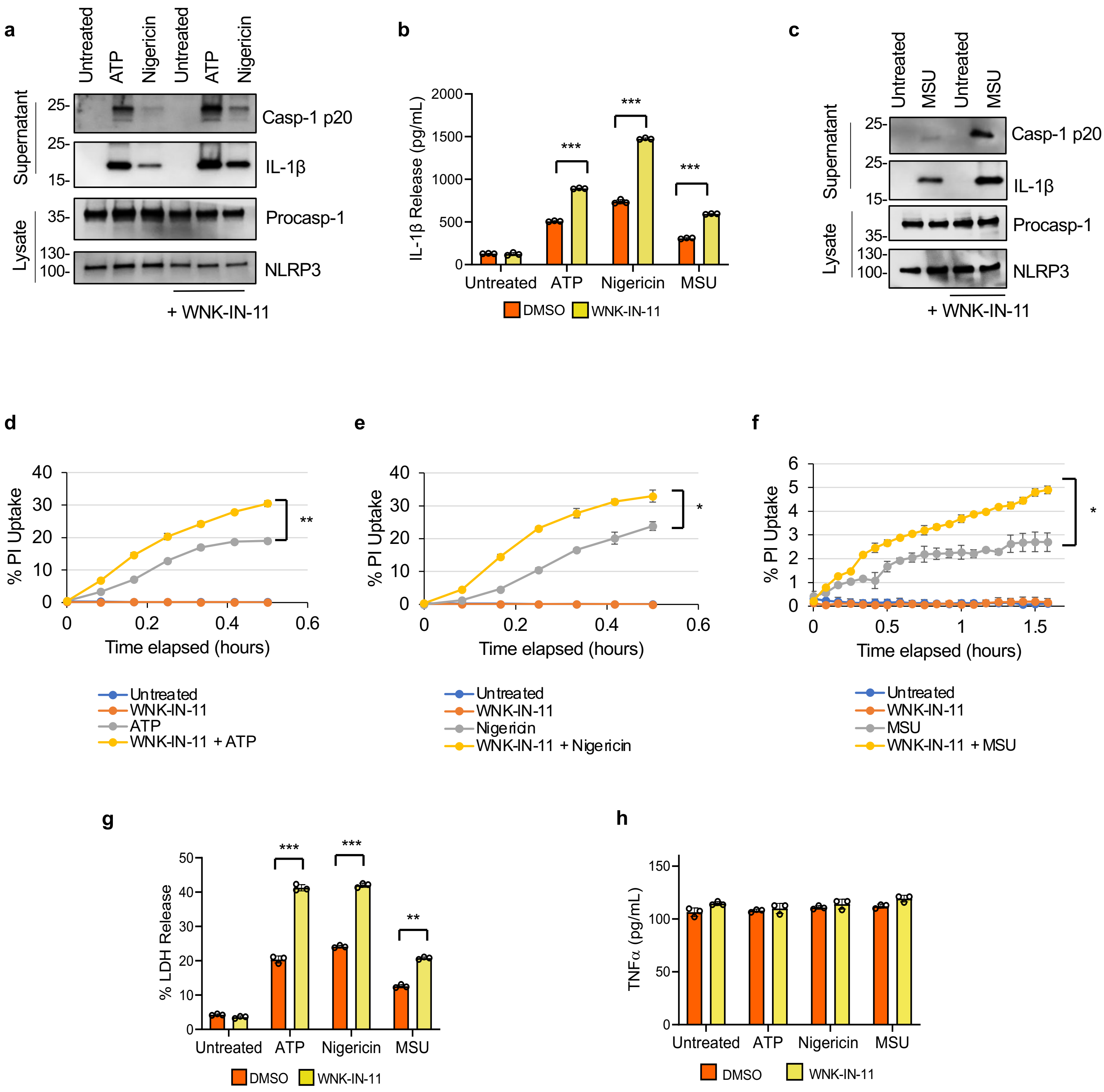

Supplementary Figure 1: WNK1 inhibition by WNK-IN-11 increases NLRP3 inflammasome activation in macrophages. **a** Immunoblots of caspase-1 p20 and mature IL-1β released in culture supernatants (Supernatant) or procaspase-1 and NLRP3 in cell lysates (Lysate) of LPS-primed (4 hours) primary wild type bone marrow macrophages pretreated with or without 1 μM WNK-IN 11 inhibitor for 10 minutes followed by 5 mM ATP or 10 μM Nigericin as indicated. **b** IL-1β release in culture supernatants of LPS-primed (4 hours) primary wild type bone marrow macrophages treated with or without WNK-IN-11 inhibitor followed by ATP, nigericin, or MSU stimulation as indicated. P values are 0.66, 0.00004, 0.00015, and 0.00004. **c** Immunoblots of caspase-1 p20 and mature IL-1β released in culture supernatants (Supernatant) or procaspase-1 and NLRP3 in cell lysates (Lysate) of LPS-primed (4 hours) primary wild type bone marrow macrophages treated with or without 1 μM WNK-IN 11 inhibitor for 10 minutes followed by MSU. **d-f** Propidium iodide uptake of LPS-primed (4 hours) primary BMDMs treated with or without WNK-IN 11 inhibitor followed by ATP (d), nigericin (e) or MSU (f) stimulation as measured on the IncuCyte over time. P value of d is 0.0046; e is 0.0366; f is 0.0069. **g-h** LDH (g) and TNFα (h) release in culture supernatants of LPS-primed (4 hours) primary wild type bone marrow macrophages treated with or without WNK-IN-11 inhibitor followed by ATP, nigericin, or MSU stimulation as indicated. P values in g are 0.078, 0.00037, 0.00032, and 0.003; h are 0.114, 0.885, 0.949, and 0.188. Results are representative of at least three independent experiments performed in duplicate or triplicate. Error bars in b, d-h are presented as mean values +/- standard deviation (S.D.), with n=3. Two-sided student's t-test, *p < 0.05, **p < 0.005, ***p < 0.0005.

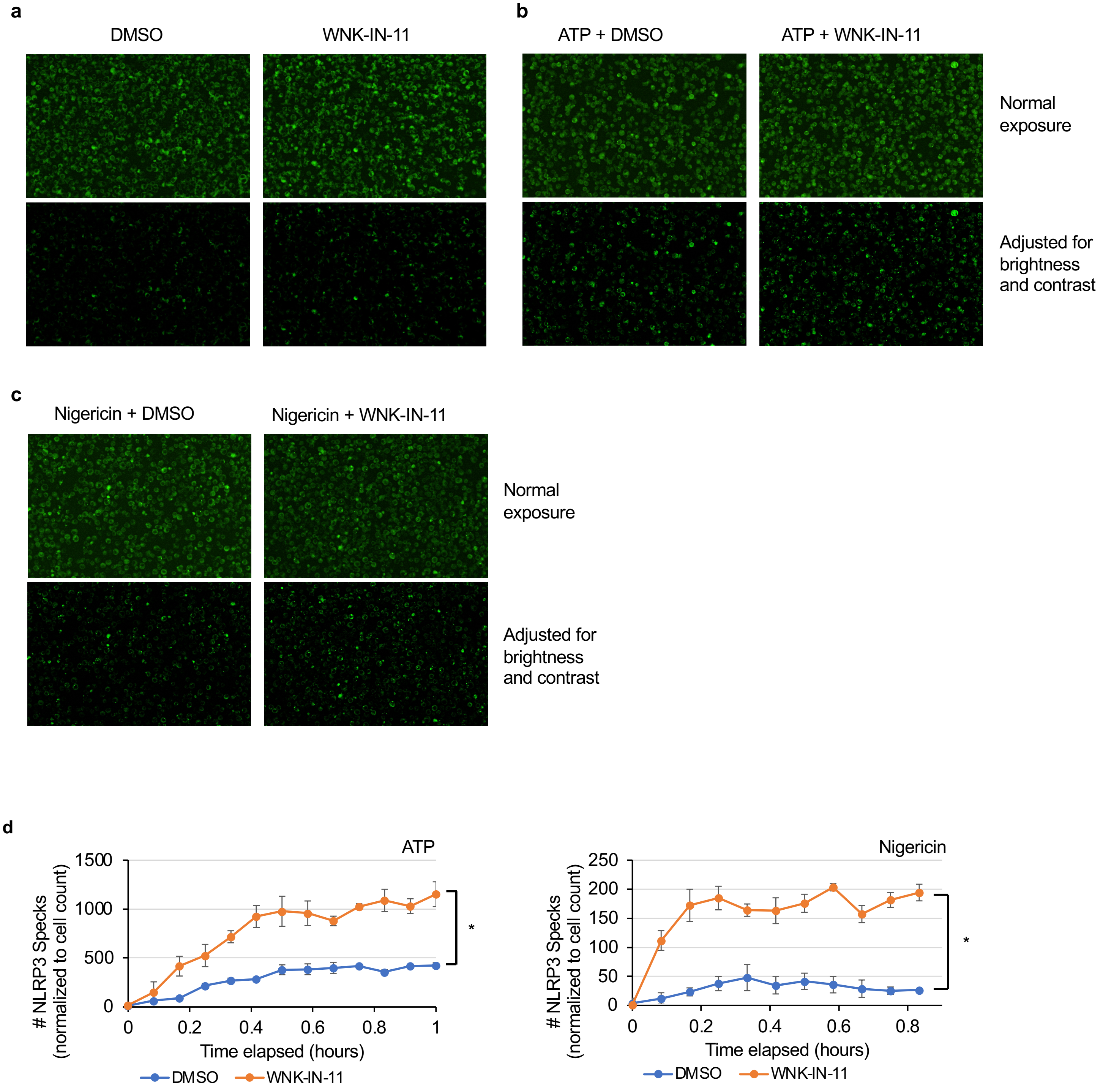

Supplementary Figure 2: WNK1 inhibition causes increased NLRP3 oligomerization. a-c IncuCyte images of LPS-primed (10 min) immortalized NLRP3-KO BMDMs stably expressing NLRP3-EGFP pretreated with DMSO or WNK-IN-11 for 10 minutes alone (**a**) or followed by ATP (**b**) or nigericin (**c**) as indicated. The bottom images in each panel are the same as the top images but their brightness and contrast were adjusted to highlight the small NLRP3-EGFP specks. (**d**) The numbers of NLRP3-EGFP specks in cells treated with ATP or nigericin as above were counted as a function of time in the IncuCyte. P values are 0.04593 and 0.04573. Data points are representative of the average count of four different images. Error bars in d are presented as mean values +/- standard deviation (S.D.). Two-sided student's t-test *$p < 0.05$.

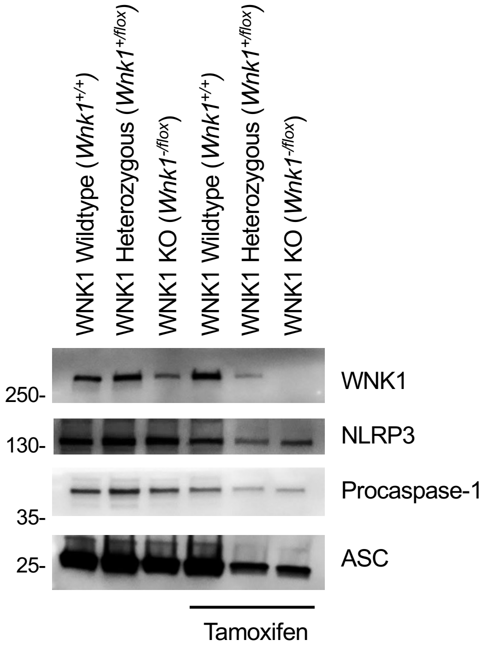

Supplementary Figure 3: *Wnk1* deletion in immortalized BMDMs carrying a floxed *Wnk1* gene under the control of the tamoxifen inducible CreERT2. Immunoblots of WNK1, NLRP3, procaspase 1, or ASC in cell lysates of immortalized BMDMs left untreated (1st to 3rd lanes) or treated (4th to 6th lanes) with 0.02mg/ml 4-Hydroxytamoxifen (4HT) for 48 hours. Results are representative of three independent experiments.

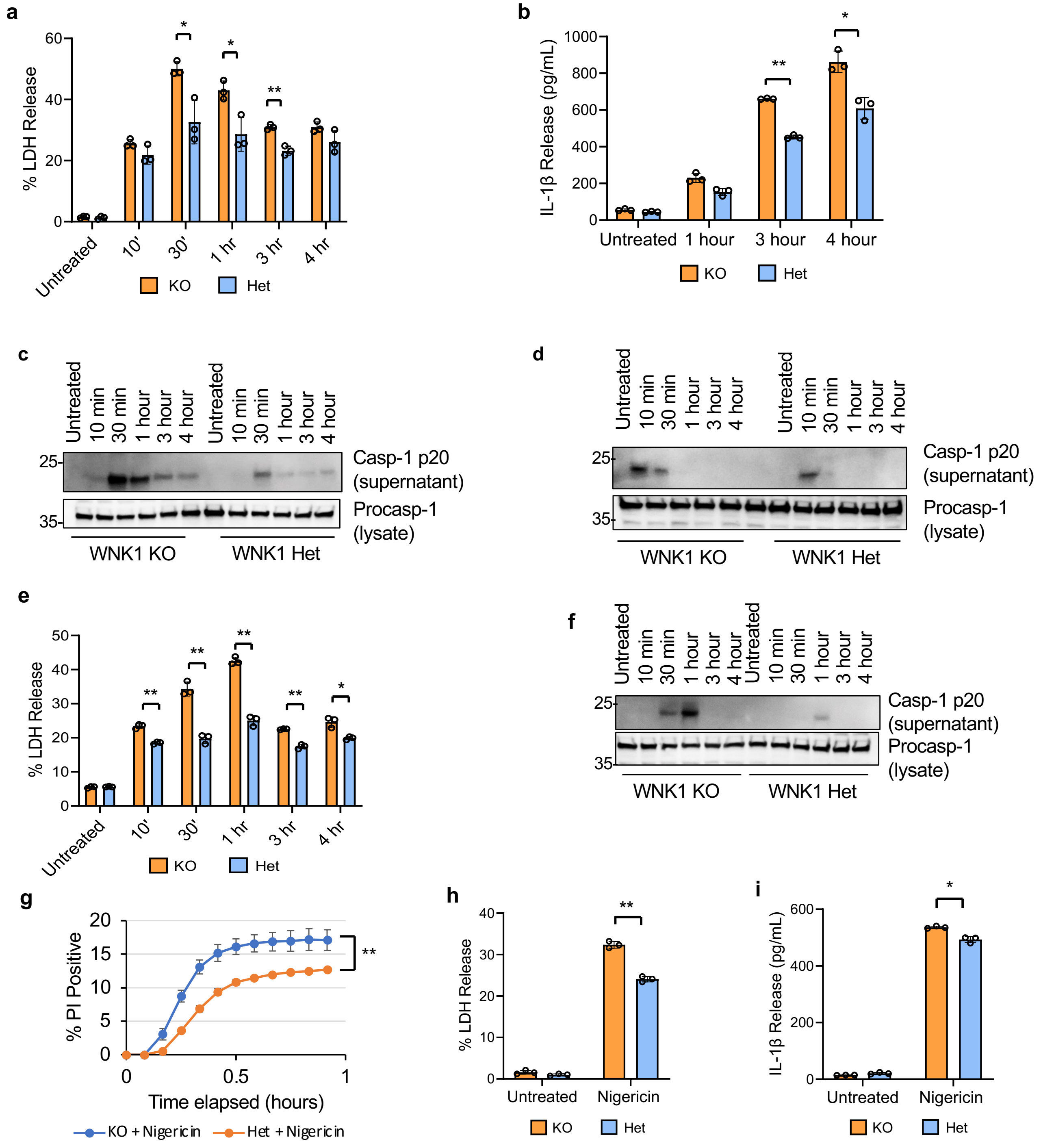

Supplementary Figure 4: Homozygous *Wnk1* knockout increases NLRP3 inflammasome activation compared to a heterozygous *Wnk1* deletion in macrophages. a, b LDH (**a**) and IL-1β (**b**) release in culture supernatants of immortalized *Wnk1*$^{-/flox}$-*CreERT2*$^{+}$ (WNK1 KO) or *WNK1*$^{+/flox}$-*CreERT2*$^{+}$ (WNK1 het) macrophages treated with tamoxifen for 48 hours prior to stimulation with LPS for the indicated times followed by ATP for 45 min. P values for a are 0.225, 0.2397, 0.04728, 0.0439, 0.002392, and 0.2347; b are 0.1382, 0.09416, 0.00120, and 0.04974. **c** Immunoblots of caspase-1 in culture supernatant (supernatant) and cell lysates (lysate) in immortalized *Wnk1*$^{-/flox}$-*CreERT2*$^{+}$ (WNK1 KO) or *WNK1*$^{+/flox}$-*CreERT2*$^{+}$ (WNK1 het) macrophages treated with tamoxifen for 48 hours prior to stimulation with LPS for the indicated times followed by ATP for 45 min. **d** Immunoblots of caspase-1 in culture supernatant (supernatant) and cell lysates (lysate) of immortalized *Wnk1*$^{-/flox}$-*CreERT2*$^{+}$ (WNK1 KO) or *WNK1*$^{+/flox}$-*CreERT2*$^{+}$ (WNK1 het) macrophages treated with tamoxifen for 48 hours prior to stimulation with PAM3csk4 the indicated times followed by ATP for 45 min. **e, f** LDH release (**e**) and Immunoblots (**f**) of caspase-1 in culture supernatant (supernatant) and cell lysates (lysate) of immortalized *Wnk1*$^{-/flox}$-*CreERT2*$^{+}$ (WNK1 KO) or *WNK1*$^{+/flox}$-*CreERT2*$^{+}$ (WNK1 het) macrophages treated with tamoxifen for 48 hours prior to stimulation with poly I:C for times indicated followed by ATP. P values for e are 0.6927, 0.00181, 0.0035, 0.00110, 0.003462, and 0.01881. **g-i** Propidium iodide uptake (**g**), LDH release (**h**), and IL-1β release (**i**) of immortalized *Wnk1*$^{-/flox}$-*CreERT2*$^{+}$ (WNK1 KO) or *WNK1*$^{+/flox}$-*CreERT2*$^{+}$ (WNK1 het) macrophages treated with tamoxifen for 48 hours prior to stimulation with LPS (4 hours) followed by nigericin (1h). P value for g is 0.0047; h are 0.1013 and 0.00250; i are 0.0645 and 0.0365. Results are representative of at least three independent experiments performed in duplicate or triplicate. Error bars in a-b, e, g-i are presented as mean values +/- standard deviation (S.D.); with n=3. Two-sided student's t-test, *p < 0.05, **p < 0.005.

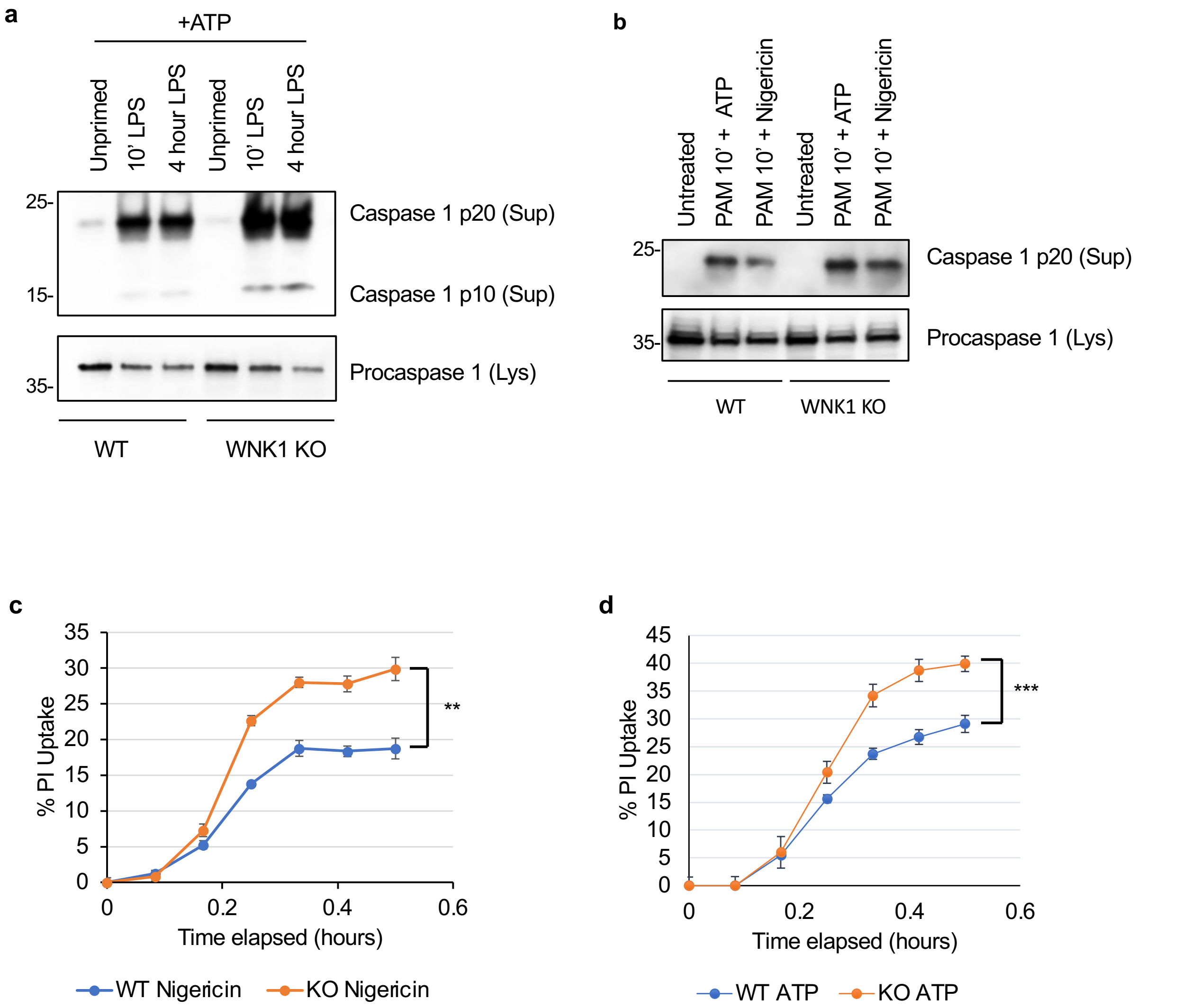

Supplementary Figure 5: WNK1 regulates NLRP3 activation independent of priming. a Immunoblots of caspase-1 p10 and p20 released in culture supernatants (Sup), and procaspase-1 in cell lysates (Lys) of unprimed, short-LPS (10 min), or long LPS-primed (4 hours) primary *Wnk1*$^{+/+}$ LysMCre^{+} (WNK1 WT) and *Wnk1*$^{flox/flox}$ LysMCre^{+} (WNK1 KO) BMDMs treated with ATP for 30 minutes. **b** Immunoblots of caspase-1 p20 released in culture supernatants (Sup) and procaspase-1 in cell lysates (Lys) of unprimed (Untreated) or short-Pam3CSK4 primed (PAM, 10 min) primary *Wnk1*$^{+/+}$ LysMCre^{+} (WNK1 WT) and *Wnk1*$^{flox/flox}$ LysMCre^{+} (WNK1 KO) BMDMs treated with ATP or nigericin for 30 minutes as indicated. **c-d** Propidium iodide uptake of short-Pam3CSK4 primed (10 min) primary *Wnk1*$^{+/+}$ LysMCre^{+} (WNK1 WT) and *Wnk1*$^{flox/flox}$ LysMCre^{+} (WNK1 KO) BMDMs treated with **c** nigericin or **d** ATP for 30 minutes as indicated. P value of c is 0.000855; d is 0.00009. Results are representative of at least three independent experiments performed in duplicate or triplicate. Error bars in c-d are presented as mean values +/- standard deviation (S.D.); with n=3. Two-sided student's t-test ***$p < 0.0005$, **$p < 0.005$.

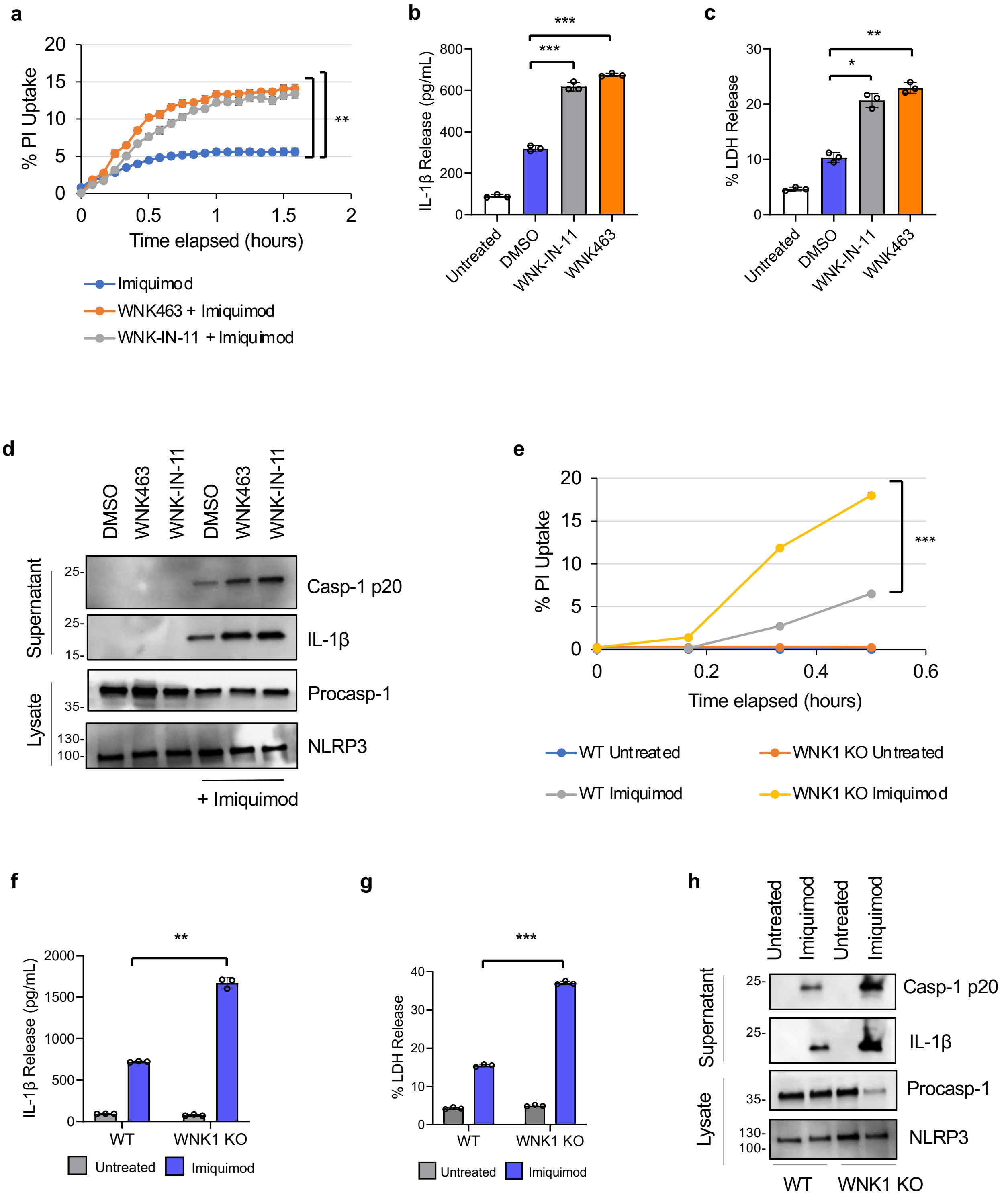

Supplementary Figure 6: WNK1 knockout increases NLPR3 inflammasome activation in imiquimod-treated macrophages. a-c Propidium iodide uptake (a), IL-1β release (b) and LDH release (c) of LPS-primed (4 hours) primary wild type bone marrow macrophages pretreated with or without 1 μM WNK 463 inhibitor or WNK-IN-11 for 10 minutes followed by 70 uM Imiquimod (R837). P values for a are 0.00394 and 0.00477; b are 0.000436 and 0.00008; c are 0.000879 and 0.0068. **d** Immunoblots of caspase-1 p20 and mature IL-1β released in culture supernatants (Supernatant) or procaspase-1 and NLRP3 in cell lysates (Lysate) of LPS-primed (4 hours) primary wild type bone marrow macrophages pretreated with or without 1 μM WNK463 inhibitor or WNK-IN 11 inhibitor for 10 minutes followed by Imiquimod. **e-g** Propidium iodide uptake (e), IL-1β release (f) and LDH release (g) of LPS-primed (4 hours) primary Wnk1$^{+/+}$ LysMCre^{+} (WNK1 WT) and Wnk1$^{flox/flox}$ LysMCre^{+} (WNK1 KO) BMDMs treated with 70 uM Imiquimod (R837). P value of e is 0.000389; f is 0.00167; g is 0.000267. **h** Immunoblots of caspase-1 p20 and mature IL-1β released in culture supernatants (Supernatant) or procaspase-1 and NLRP3 in cell lysates (Lysate) of LPS-primed (4 hours) primary Wnk1$^{+/+}$ LysMCre^{+} (WNK1 WT) and Wnk1$^{flox/flox}$ LysMCre^{+} (WNK1 KO) BMDMs treated with 70 uM Imiquimod (1h). Results are representative of at least three independent experiments performed in duplicate or triplicate. Error bars in a-c, e-g are presented as mean values +/- standard deviation (S.D.); with n=3. Two-sided student's t-test, *p<0.05, **p < 0.005, ***p < 0.0005.

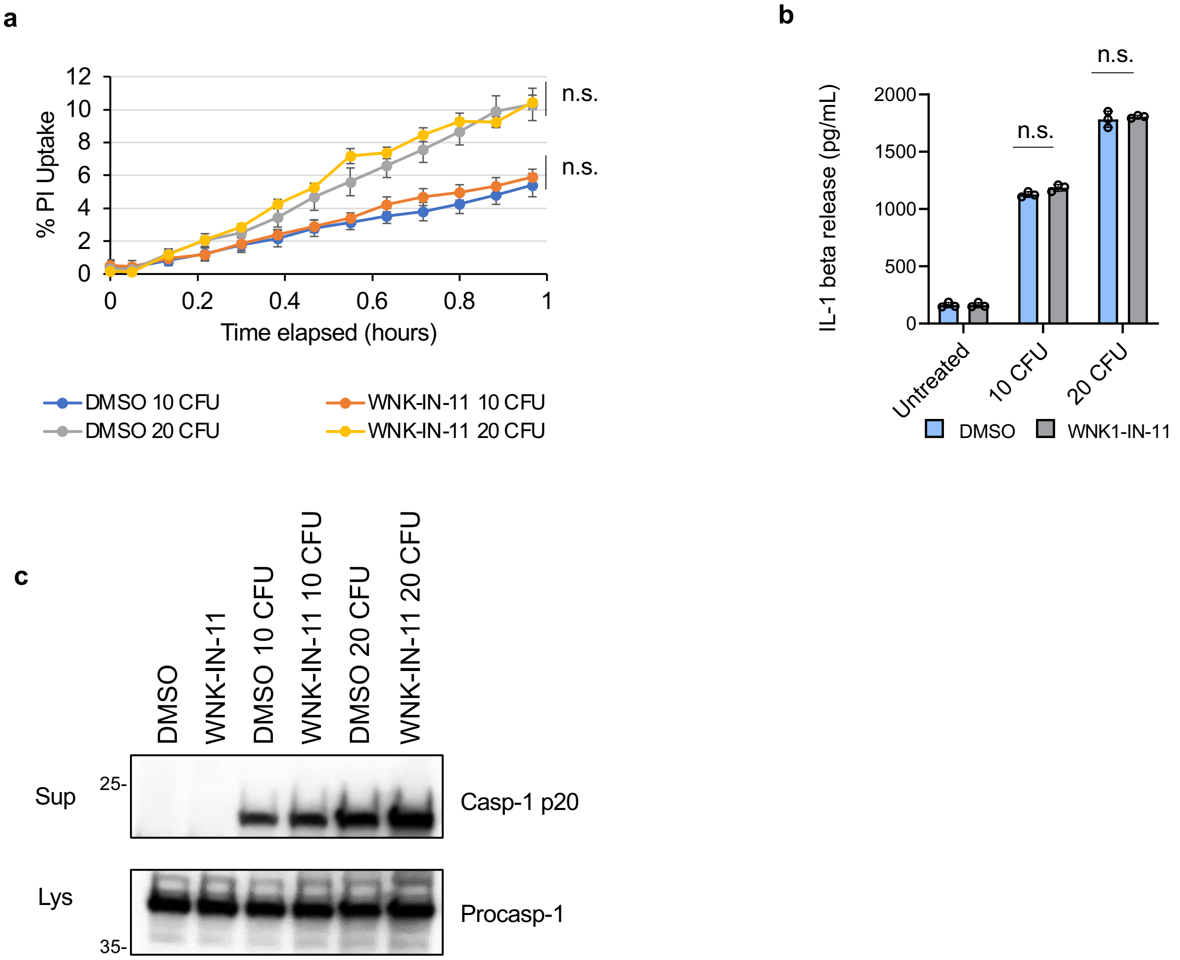

Supplementary Figure 7: WNK1 does not regulate the NLRC4 inflammasome. a, b Propidium iodide uptake (**a**) and IL-1β release (**b**) of LPS-primed (4 hours) immortalized NLRP3/AIM2 double knockout (DKO) BMDMs pretreated with DMSO or 1μM WNK-IN-11 for 15 minutes prior to infection with the indicated CFUs of Salmonella. P values in a are 0.1433 and 0.5293; b are 0.9897, 0.1835, and 0.6235. **c** Immunoblots of caspase-1 p20 released in culture supernatants (Sup) and procaspase-1 in cell lysates (Lys) of LPS-primed (4 hours) immortalized NLRP3/AIM2 DKO BMDMs pretreated with DMSO or 1μM WNK-IN-11 for 15 minutes prior to infection with the indicated CFUs of Salmonella. Results are representative of at least three independent experiments performed in duplicate or triplicate. Error bars in a-b are presented as mean values +/- standard deviation (S.D.); with n=3. Two-sided student's t-test results in this figure are not significant (n.s.).

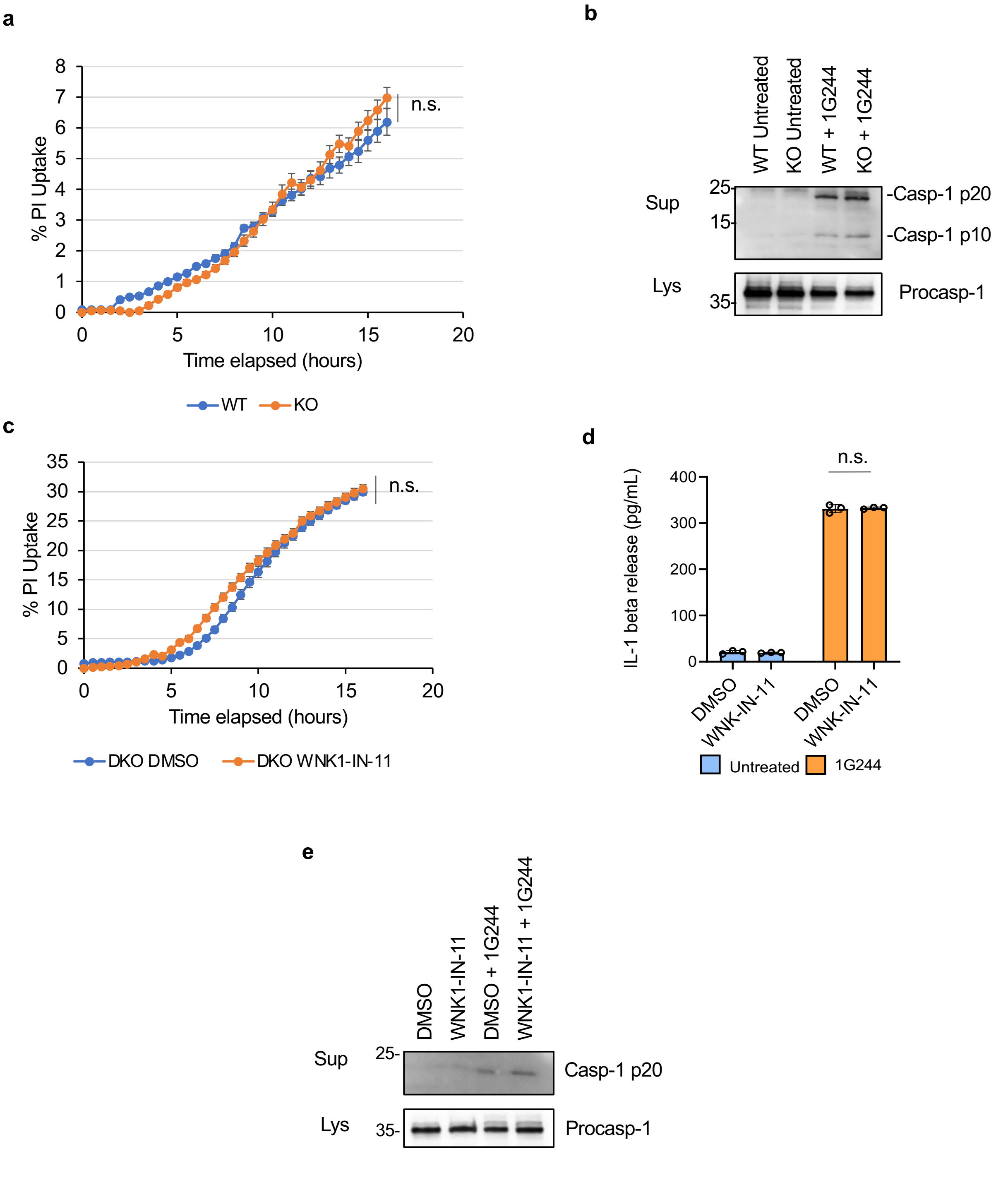

Supplementary Figure 8: WNK1 does not regulate the NLRP1 inflammasome. a Propidium iodide uptake of LPS-primed (4 hours) primary *Wnk1*$^{+/+}$ LysMCre^{+} (WNK1 WT) and *Wnk1*$^{flox/flox}$ LysMCre^{+} (WNK1 KO) BMDMs treated with 20 µM 1G244 for 16 hours. P value 0.0769. **b** Immunoblots of caspase-1 p10 and caspase-1 p20 released in culture supernatants (Sup) and procaspase-1 in cell lysates (Lys) of LPS-primed (4 hours) primary *Wnk1*$^{+/+}$ LysMCre^{+} (WNK1 WT) and *Wnk1*$^{flox/flox}$ LysMCre^{+} (WNK1 KO) BMDMs treated with 20 µM 1G244 for 16 hours. **c** Propidium iodide uptake of LPS-primed (4 hours) immortalized NLRP3/AIM2 DKO BMDMs pretreated with DMSO or 1µM WNK-IN-11 for 15 minutes prior to treatment with 20 µM 1G244 for 16 hours. P value 0.6415. **d** IL-1β release of LPS-primed (4 hours) immortalized NLRP3/AIM2 DKO BMDMs pretreated with DMSO or 1µM WNK-IN-11 for 15 minutes prior to treatment with 20 µM 1G244 for 16 hours. P values are 0.4695 and 0.8388. **e** Immunoblots of caspase-1 p20 released in culture supernatants (Sup) and procaspase-1 in cell lysates (Lys) of LPS-primed (4 hours) immortalized NLRP3/AIM2 DKO BMDMs pretreated with DMSO or 1µM WNK-IN-11 for 15 minutes prior to treatment with 20 µM 1G244 for 16 hours. Results are representative of at least three independent experiments performed in duplicate or triplicate. Error bars in a, c-d are presented as mean values +/- standard deviation (S.D.); with n=3. Two-sided student's t-test results in this figure are not significant (n.s.).

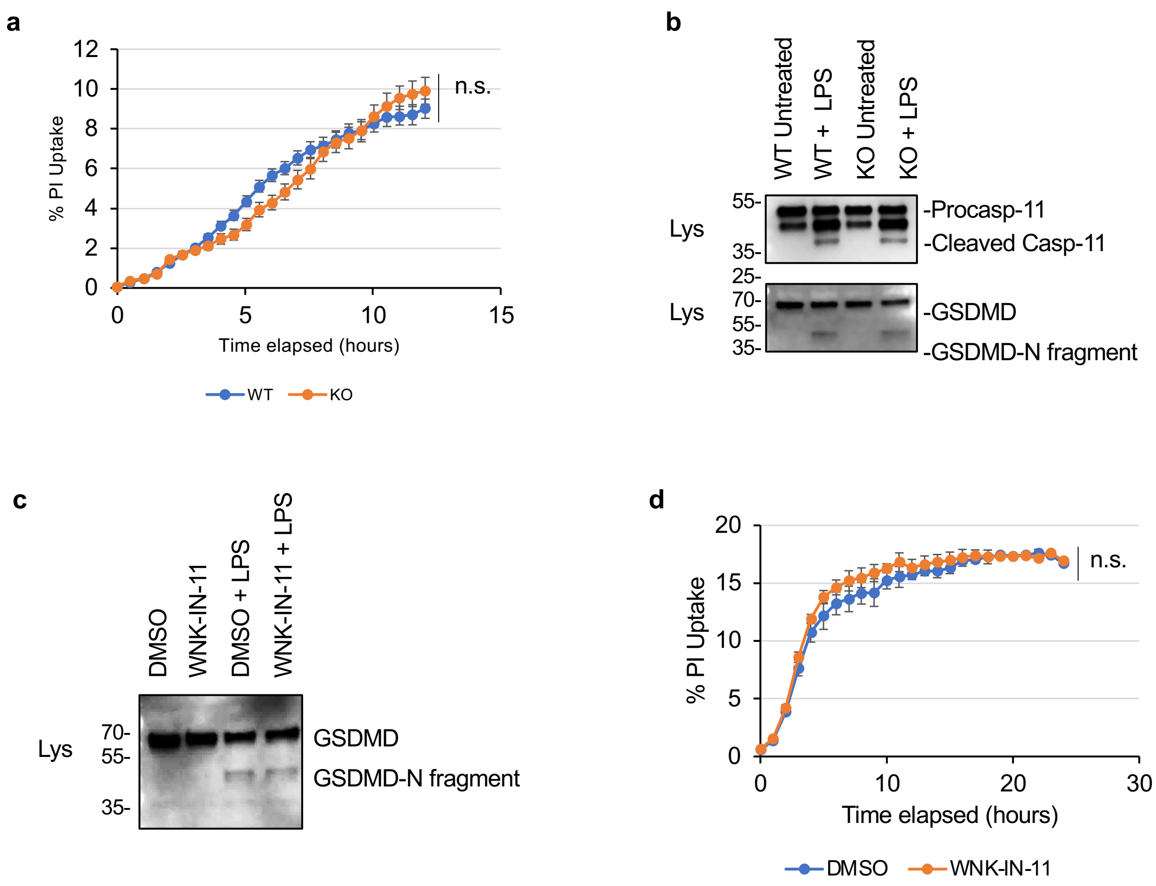

Supplementary Figure 9: WNK1 does not regulate non-canonical inflammasome activation. a Propidium iodide uptake of PAM-primed (4 hours) primary *Wnk1*$^{+/+}$ LysMCre^{+} (WNK1 WT) and *Wnk1*$^{flox/flox}$ LysMCre^{+} (WNK1 KO) BMDMs transfected with 1 μg/mL LPS. P value is 0.18290. **b** Immunoblots of caspase 11 and GSDMD in cell lysates of PAM-primed (4 hours) primary *Wnk1*$^{+/+}$ LysMCre^{+} (WNK1 WT) and *Wnk1*$^{flox/flox}$ LysMCre^{+} (WNK1 KO) BMDMs transfected with 1 μg/mL LPS. **c** Immunoblot of full-length and cleaved GSDMD in cell lysates of PAM-primed (4 hours) primary NLRP3 KO BMDMs pretreated with DMSO or 1μM WNK-IN-11 for 10 minutes prior to transfection with 1 μg/mL LPS. **d** Propidium iodide uptake of PAM-primed (4 hours) primary wildtype BMDMs pretreated with DMSO or 1μM WNK-IN-11 for 10 minutes prior to transfection with 1 μg/mL LPS. P value is 0.41225. Results are representative of at least three independent experiments performed in duplicate or triplicate. Error bars in a, d are presented as mean values +/- standard deviation (S.D.); with n=3. Two-sided student's t-test results in this figure are not significant (n.s.).

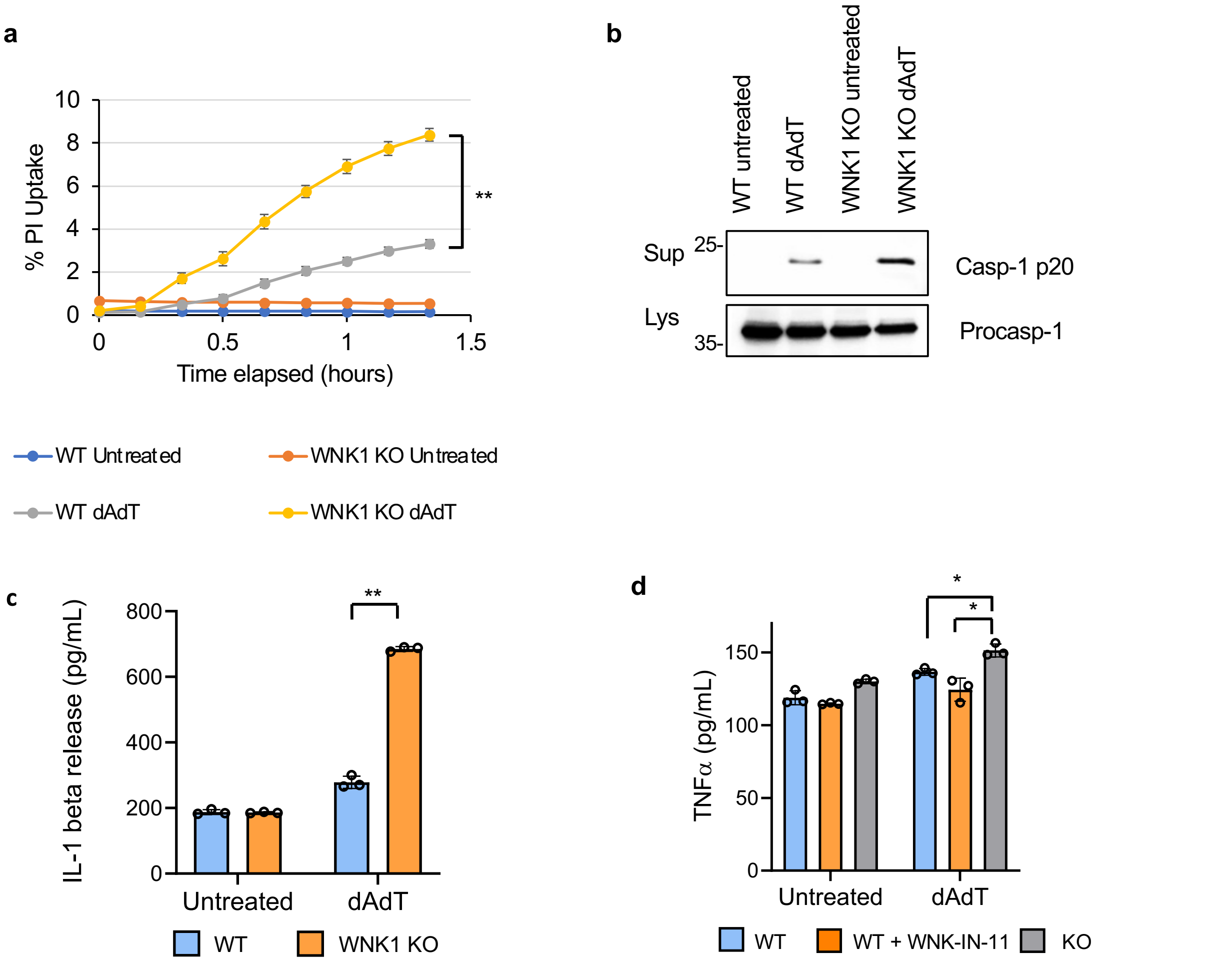

Supplementary Figure 10: WNK1 regulates the AIM2 inflammasome. a Propidium iodide uptake of PAM-primed (4 hours) primary *Wnk1*$^{+/+}$ LysMCre^{+} (WNK1 WT) and *Wnk1*$^{flox/flox}$ LysMCre^{+} (WNK1 KO) BMDMs transfected with 1 µg/mL Poly(dA:dT). **b** Immunoblots of caspase-1 p20 released in culture supernatants (Sup) and procaspase-1 in cell lysates (Lys) of PAM-primed (4 hours) primary *Wnk1*$^{+/+}$ LysMCre^{+} (WNK1 WT) and *Wnk1*$^{flox/flox}$ LysMCre^{+} (WNK1 KO) BMDMs transfected with 1 µg/mL Poly(dA:dT). **c** IL-1β release and **d** TNF-α release of PAM-primed (4 hours) primary *Wnk1*$^{+/+}$ LysMCre^{+} (WNK1 WT) and *Wnk1*$^{flox/flox}$ LysMCre^{+} (WNK1 KO) BMDMs transfected with 1 µg/mL Poly(dA:dT). P values in a are 0.003866; c 0.77247 and 0.0005353; and d 0.02937 and 0.00677. Results are representative of at least three independent experiments performed in duplicate or triplicate. Error bars in a, c-d are presented as mean values +/- standard deviation (S.D.); with n=3. Two-sided student's t-test *p < 0.05, **p<0.005.

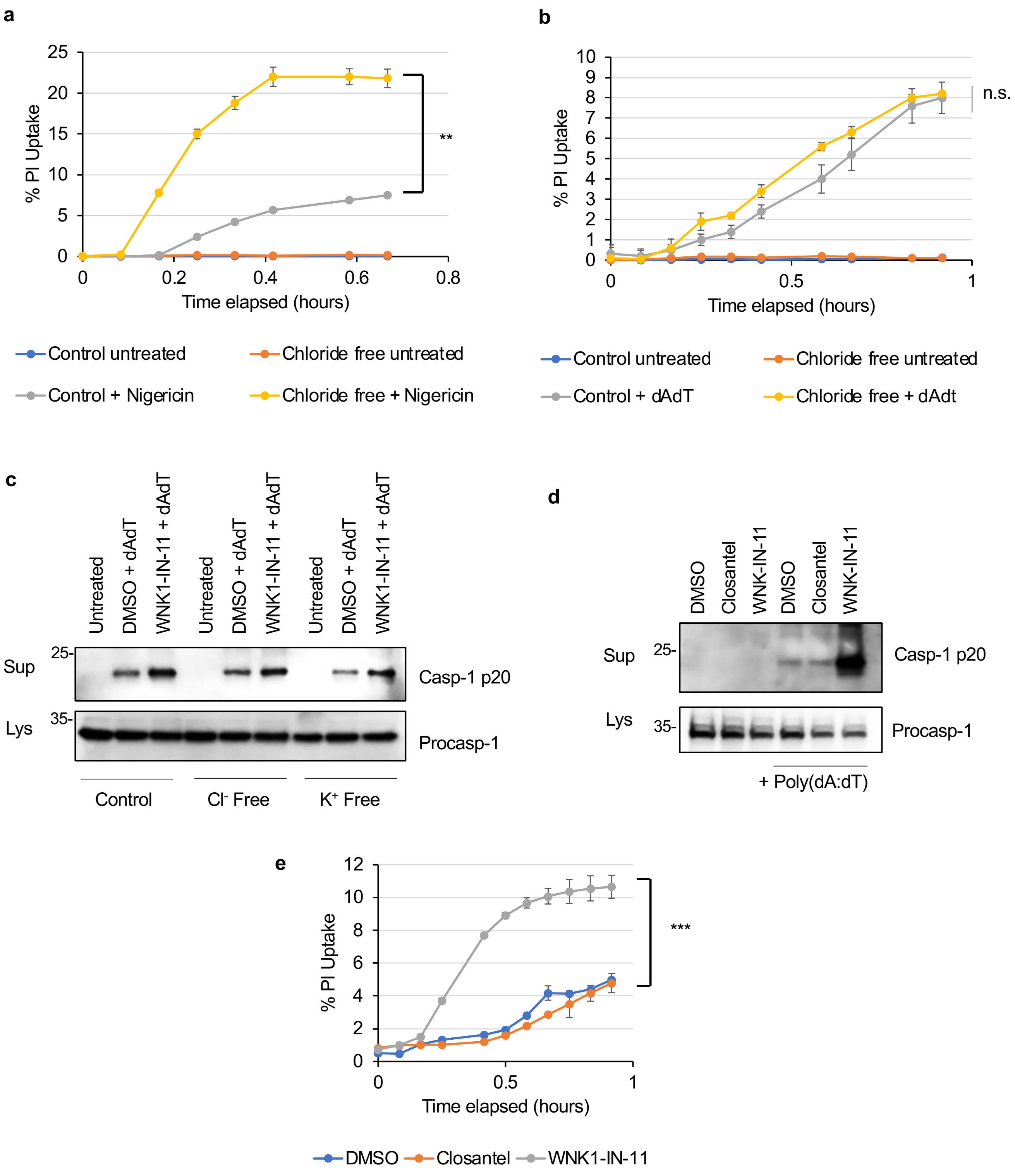

Supplementary Figure 11: WNK1 regulates AIM2 by a chloride/channel independent mechanism. a-b Propidium iodide uptake of PAM-primed (4 hours) primary wildtype BMDMs incubated for one hour in control or chloride free PBS prior to **a** treatment with nigericin or **b** transfection with 1 µg/mL Poly(dA:dT). P value of a is 0.000775; of b is 0.3659. **c** Immunoblots of caspase-1 p20 released in culture supernatants (Sup) and procaspase-1 in cell lysates (Lys) of PAM-primed (4 hours) wild type primary BMDMs pretreated with DMSO or 1µM WNK-IN-11 for 10 minutes and then incubated for one hour in control, chloride free, or potassium free media prior to transfection with 1 µg/mL Poly(dA:dT). **d** Immunoblots of caspase-1 p20 released in culture supernatants (Sup) and procaspase-1 in cell lysates (Lys) of PAM-primed (4 hours) wild type primary BMDMs pretreated with DMSO, 2µM Closantel, or 1µM WNK-IN-11 for 10 minutes prior to transfection with 1 µg/mL Poly(dA:dT). **e** Propidium iodide uptake of PAM-primed (4 hours) wild type primary BMDMs pretreated with DMSO, 2µM Closantel, or 1µM WNK-IN-11 for 10 minutes prior to transfection with 1 µg/mL Poly(dA:dT). P value is 0.0002154. Results are representative of at least three independent experiments performed in duplicate or triplicate. Error bars in a-b, e are presented as mean values +/- standard deviation (S.D.); with n=3. Two-sided student's t-test n.s. not significant, ***p < 0.0005, **p<0.005.

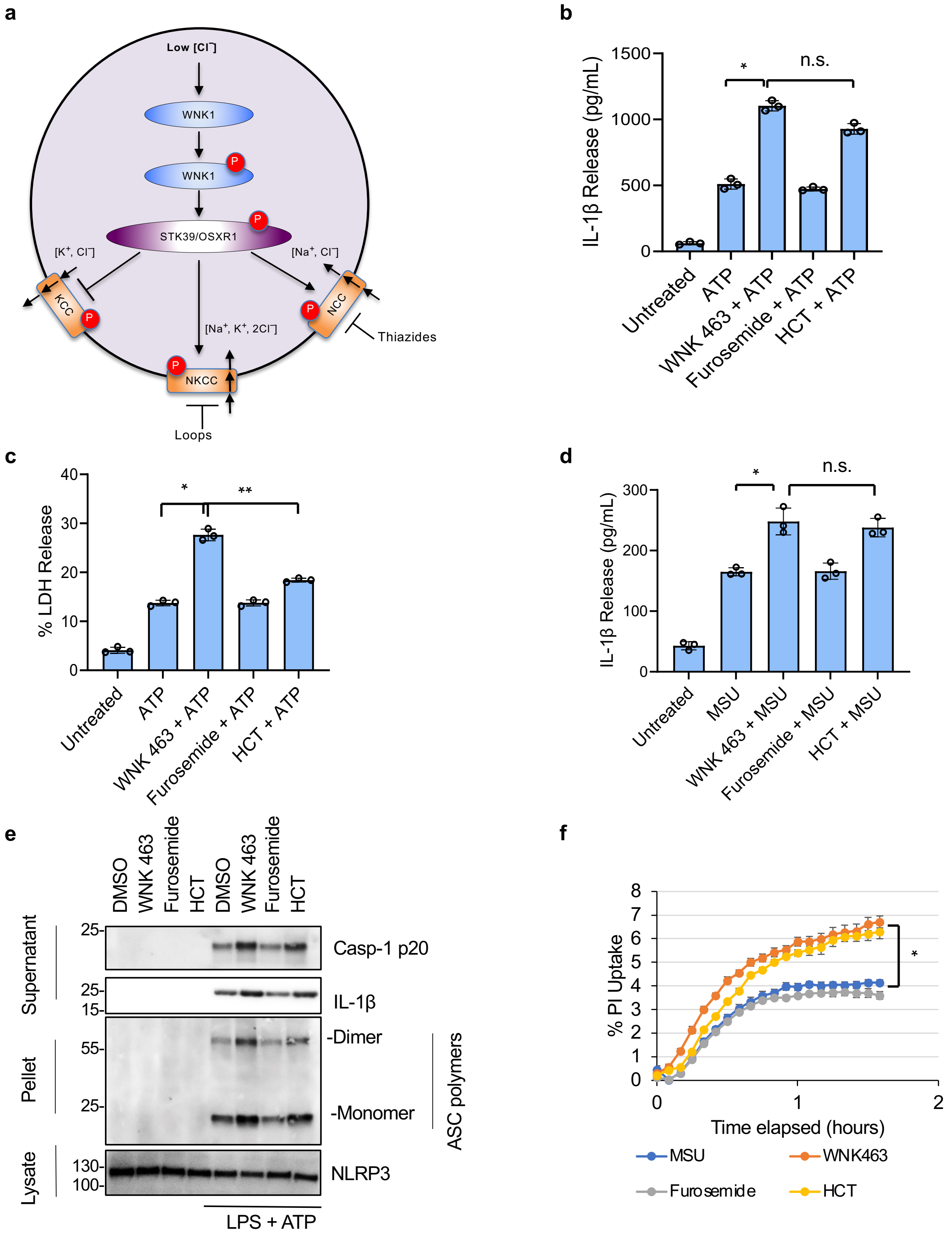

Supplementary Figure 12: Pharmacological inhibition of SLC12A3 (NCC) cotransporter by Hydrochlorothiazide increases NLRP3 inflammasome activation. **a** Model illustrating the WNK1 signaling pathway initiated by low intracellular chloride concentrations leading to activation of WNK1 which phosphorylates and activates STK39 and OSXR1which in turn phosphorylate and activate ion cotransporters, the targets of thiazide and loop diuretics. **b, c** IL-1β (**b**), and LDH (**c**) release in culture supernatants of LPS primed (4 hours) primary wild type BMDMs pretreated with 1 µM WNK463 inhibitor, 100 µM Furosemide, or 100 µM HCT for 30 minutes prior to stimulation with ATP. P values of b are 0.005569, 0.05348; c are 0.005186, 0.003590. **d** IL-1β release in culture supernatants of LPS primed (4 hours) primary wild type BMDMs pretreated with 1 µM WNK463 inhibitor, 100 µM Furosemide, or 100 µM HCT for 30 minutes prior to stimulation with MSU. P values are 0.03442, 0.1483. **e** Immunoblots of caspase-1 p20 and mature IL-1β released in culture supernatants (Supernatants), NLRP3 in cell lysates (Lysates) or ASC in NP40-insoluble pellets (Pellet) of LPS-primed (4 hours) primary wild type BMDMs pretreated with 1 µM WNK463 inhibitor, 100 µM Furosemide, or 100 µM HCT for 30 minutes prior to stimulation with ATP. **f** Propidium iodide uptake of LPS primed (4 hours) primary wild type cells pretreated with 100 µM diuretics for 30 minutes prior to stimulation with MSU. P value 0.01249. Results are representative of at least three independent experiments performed in duplicate or triplicate. Error bars in b-d, f are presented as mean values +/- standard deviation (S.D.); with n=3. Two-sided student's t-test, n.s. not significant, $^*p < 0.05$, $^{**}p < 0.005$.

a

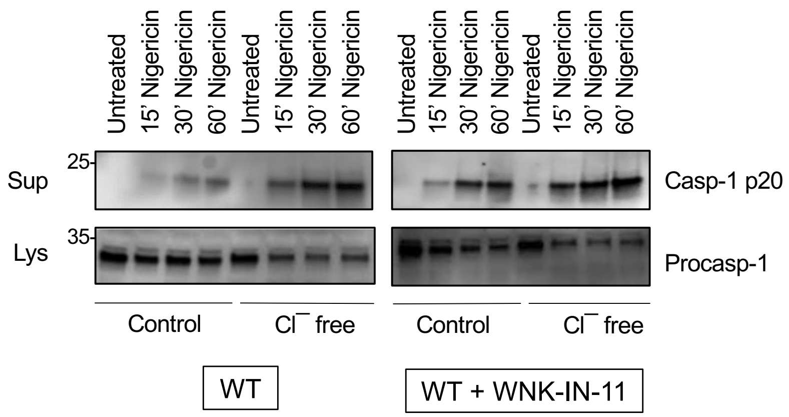

b

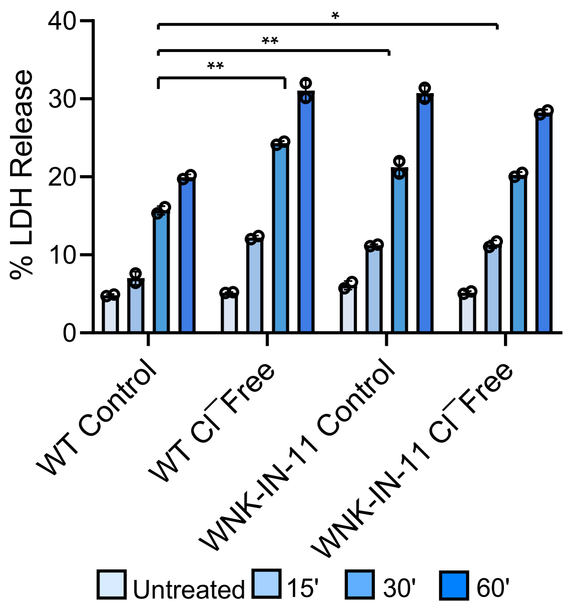

Supplementary Figure 13: WNK1 regulates NLRP3 inflammasome activation by a chloride-sensing mechanism. a Immunoblots of caspase-1 p20 released in culture supernatants (Sup) or procaspase-1 in cell lysates (Lys) of LPS-primed (4 hours) primary wild type bone marrow macrophages pretreated with or without 1 µM WNK-IN 11 inhibitor for 10 minutes followed by incubation in the indicated isotonic salt solution 1 hour prior to stimulation with nigericin for the indicated times. **b** LDH release in cell culture supernatants of LPS-primed (4 hours) primary wild type bone marrow macrophages pretreated with or without 1 µM WNK-IN 11 inhibitor for 10 minutes followed by incubation in the indicated isotonic salt solution 1 hour prior to stimulation with nigericin for the indicated times. P values left to right are 0.002640, 0.002164, 0.00829. Results are representative of at least three independent experiments performed in duplicate or triplicate. Error bars in b are presented as mean values +/- standard deviation (S.D.); with n=3. Two-sided student's t-test, *p < 0.05, **p < 0.005.

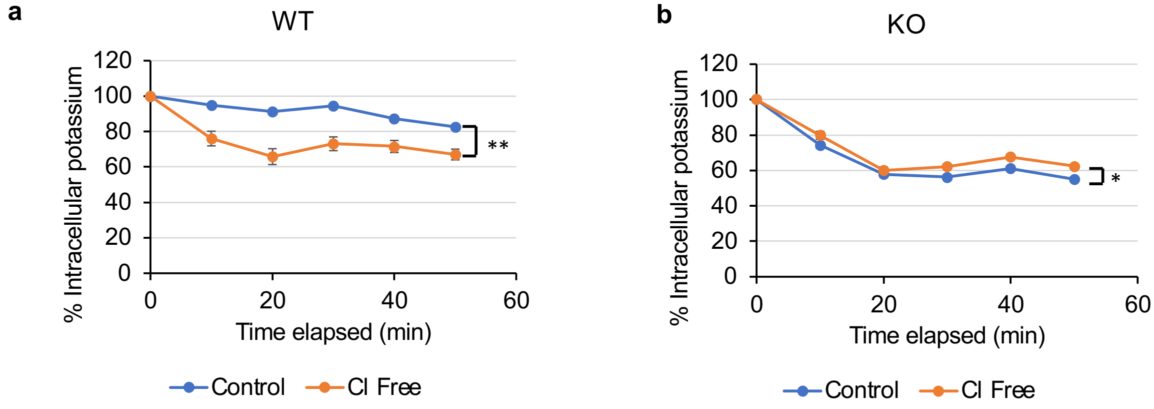

Supplementary Figure 14: Effect of chloride-free medium on intracellular potassium level. a, b Quantification of intracellular potassium of LPS-primed (4 hours) primary *Wnk1*$^{+/+}$ LysMCre^{+} (WNK1 WT) and *Wnk1*$^{flox/flox}$ LysMCre^{+} (WNK1 KO) BMDMs following treatment with nigericin for the indicated times in chloride-free media. P value of a is 0.004518; b is 0.008098. Results are representative of at least three independent experiments performed in duplicate or triplicate. Error bars in a-b are presented as mean values +/- standard deviation (S.D.); with n=3. Two-sided student's t-test, *p < 0.05, **p< 0.005.

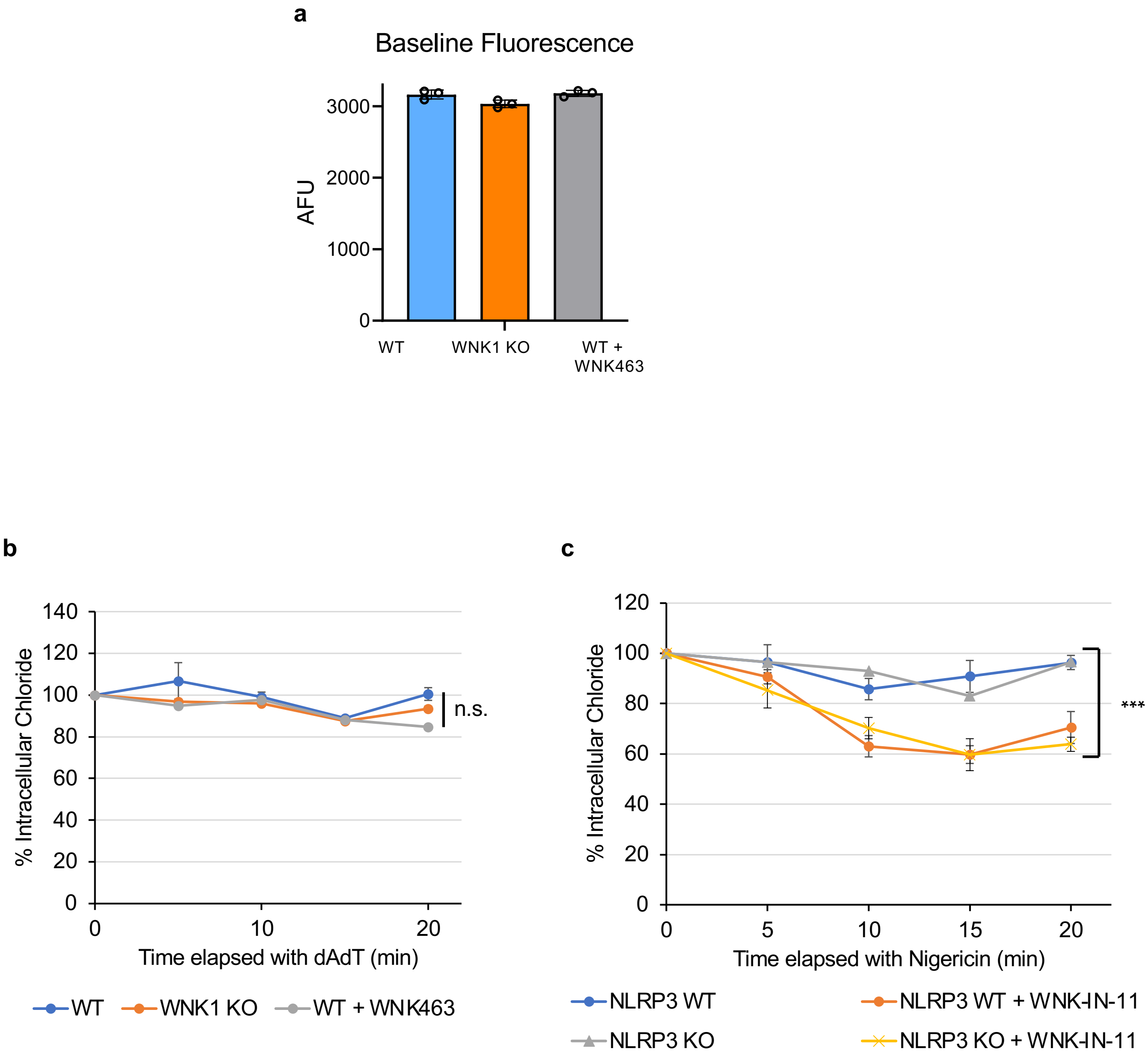

Supplementary Figure 15: a Measurement of baseline fluorescence of primary Wnk1$^{+/+}$ LysMCre^{+} (WNK1 WT), Wnk1$^{flox/flox}$ LysMCre^{+} (WNK1 KO), or wild type BMDMs pretreated with 1 μM WNK463 inhibitor where AFU= average fluorescence units. **b** Quantification of intracellular chloride in primary *Wnk1*$^{+/+}$ LysMCre^{+} (WNK1 WT), *Wnk1*$^{flox/flox}$ LysMCre^{+} (WNK1 KO), or WNK463-treated wild type BMDMs following dAdT transfection. P value 0.2627. **c** Quantification of intracellular chloride in primary wild type or NLRP3 knockout BMDMs treated with or without 1 μM WNK-IN 11 inhibitor. P value 0.000135. Results are representative of at least three independent experiments performed in duplicate or triplicate. Error bars in a-c are presented as mean values +/- standard deviation (S.D.); with n=3. Two-sided student's t-test, n.s. not significant, ***p < 0.0005.

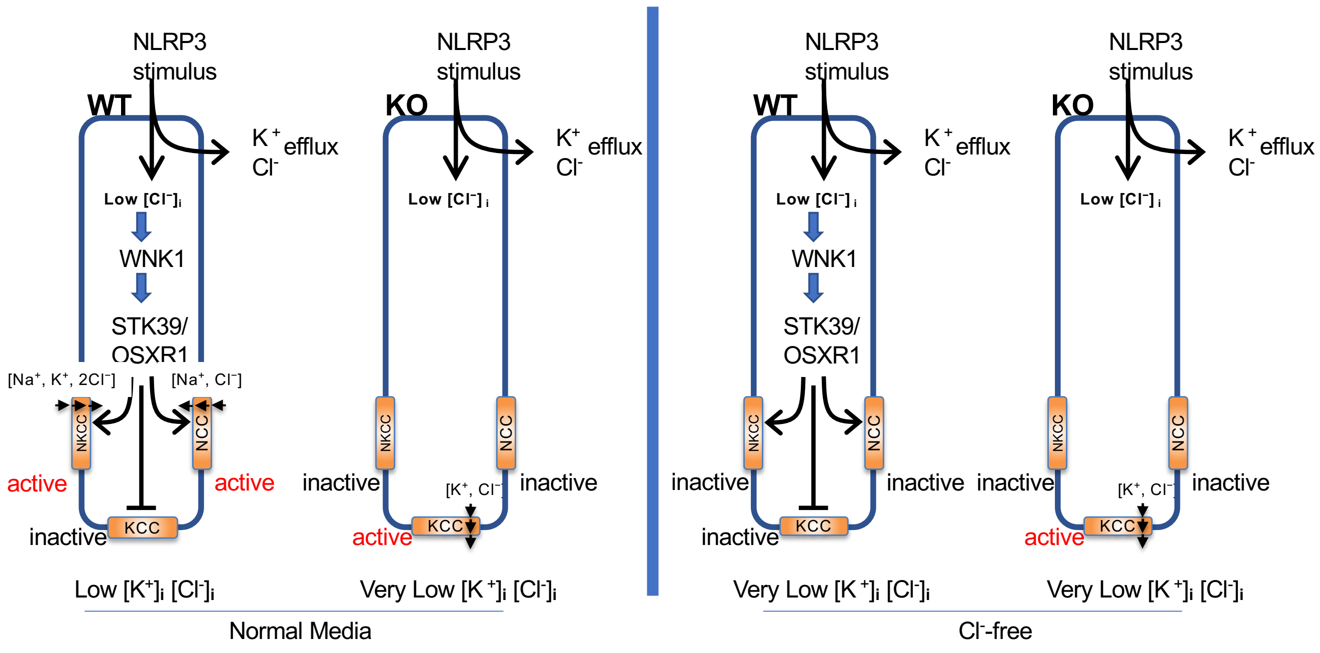

Supplementary Figure 16: Model illustrating regulation of cation-chloride cotransporters by WNK1 signaling. Low intracellular chloride levels induced by NLRP3 stimuli activates WNK1 which phosphorylates and activates STK39 and OSXR1, in turn phosphorylating and activating cation-chloride cotransporters NCC, NKCC and KCC. Left panels: in WT cells incubated in normal medium (left) WNK1 signaling activates NCC and NKCC and inhibits KCC to offset chloride and potassium loss. In WNK-KO cells incubated in normal medium (right) the absence of WNK1 signaling prevents NCC and NKCC activation and activates KCC leading to greater chloride and potassium loss. Right panels: in WT cells incubated in chloride-free medium (left), although WNK1 signaling can activate NCC and NKCC and inhibit KCC, the NCC and NKCC cotransporters remain inactive because they require extracellular chloride for their co-transporter activity. This leads to greater loss of intracellular chloride and potassium causing very low intracellular chloride and potassium levels. In WNK-KO cells incubated in chloride-free medium (right) the WNK1 deficiency, inactivation of NCC and NKCC by the absence of extracellular chloride, and activation of KCC cause a very similar drop in intracellular chloride and potassium levels.

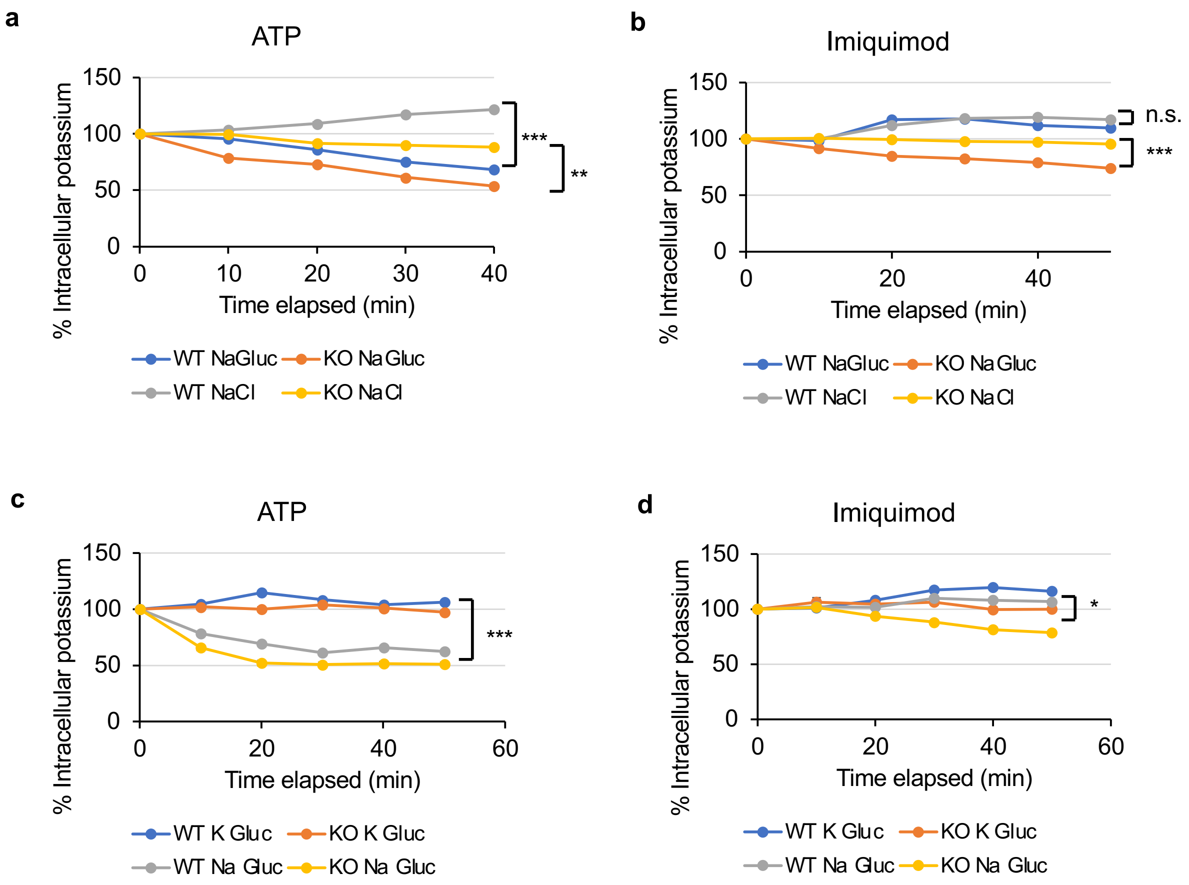

Supplementary Figure 17: Excess extracellular chloride or potassium, but not sodium, inhibits potassium efflux. a, b Intracellular potassium levels in primary *Wnk1*$^{+/+}$ LysMCre^{+} (WNK1 WT) and *Wnk1*$^{flox/flox}$ LysMCre^{+} (WNK1 KO) BMDMs stimulated with ATP (**a**) or imiquimod (**b**) in PBS infused with 130 mM NaCl or NaGluconate. **c**, **d** Intracellular potassium levels in primary *Wnk1*$^{+/+}$ LysMCre^{+} (WNK1 WT) and *Wnk1*$^{flox/flox}$ LysMCre^{+} (WNK1 KO) BMDMs stimulated with ATP (**c**) or imiquimod (**d**) in 65 mM KGluconate or NaGluconate in PBS. The potassium levels were expressed as percentage relative to untreated cells in PBS alone. P values in a are 0.00001, 0.00170; in b are 0.9160, 0.000377; in c are 0.001646, 0.0158; in d is 0.01452. Results are representative of at least three independent experiments performed in duplicate or triplicate. Error bars in a-d are presented as mean values +/- standard deviation (S.D.); with n=3. Two-sided student's t-test, n.s. not significant, *$p < 0.05$, **$p < 0.005$, ***$p < 0.0005$.

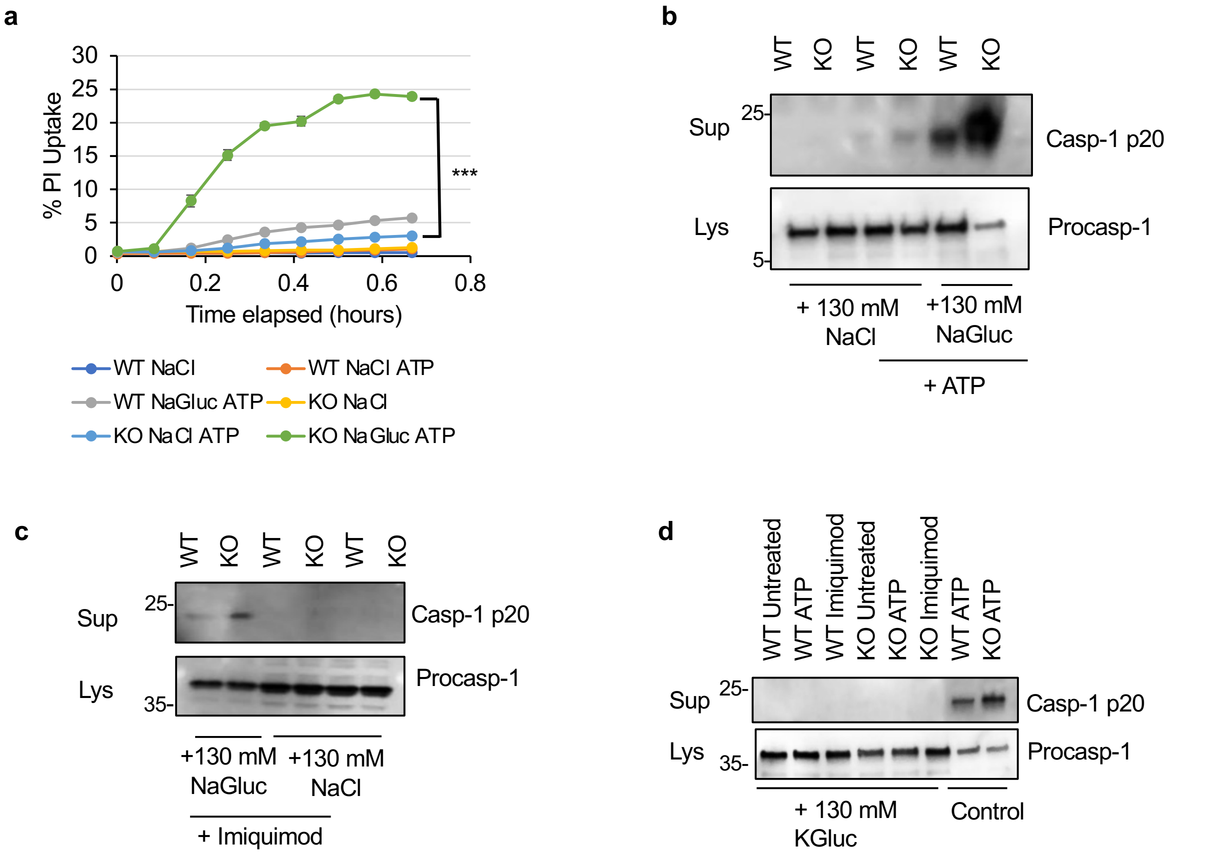

Supplementary Figure 18: Excess extracellular chloride or potassium, but not sodium, inhibits NLRP3 activation with or without WNK1. **a** Propidium iodide uptake of LPS-primed (4 hours) primary *Wnk1*$^{+/+}$ LysMCre^{+} (WNK1 WT) and *Wnk1*$^{flox/flox}$ LysMCre^{+} (WNK1 KO) BMDMs incubated in 130 mM NaCl or NaGluconate in Opti-MEM for one hour prior to stimulation with ATP. P value 0.000172. **b** Immunoblots of caspase-1 p20 released in culture supernatants (Sup) and procaspase-1 in cell lysates (Lys) of LPS-primed (4 hours) primary *Wnk1*$^{+/+}$ LysMCre^{+} (WNK1 WT) and *Wnk1*$^{flox/flox}$ LysMCre^{+} (WNK1 KO) BMDMs incubated in 130 mM NaCl or NaGluconate in Opti-MEM for one hour prior to stimulation with ATP. **c** Immunoblots of caspase-1 p20 released in culture supernatants (Sup) and procaspase-1 in cell lysates (Lys) of LPS-primed (4 hours) primary *Wnk1*$^{+/+}$ LysMCre^{+} (WNK1 WT) and *Wnk1*$^{flox/flox}$ LysMCre^{+} (WNK1 KO) BMDMs incubated with 130 mM NaGluconate or NaCl in Opti-MEM for one hour prior to stimulation with imiquimod. **d** Immunoblots of caspase-1 p20 released in culture supernatants (Sup) and procaspase-1 in cell lysates (Lys) of LPS-primed (4 hours) primary *Wnk1*$^{+/+}$ LysMCre^{+} (WNK1 WT) and *Wnk1*$^{flox/flox}$ LysMCre^{+} (WNK1 KO) BMDMs incubated in Opti-MEM (control) or 130 mM KGluconate in Opti-MEM. Results are representative of at least three independent experiments performed in duplicate or triplicate. Error bars in a are presented as mean values +/- standard deviation (S.D.); with n=3. Two-sided student's t-test ***p<0.0005.

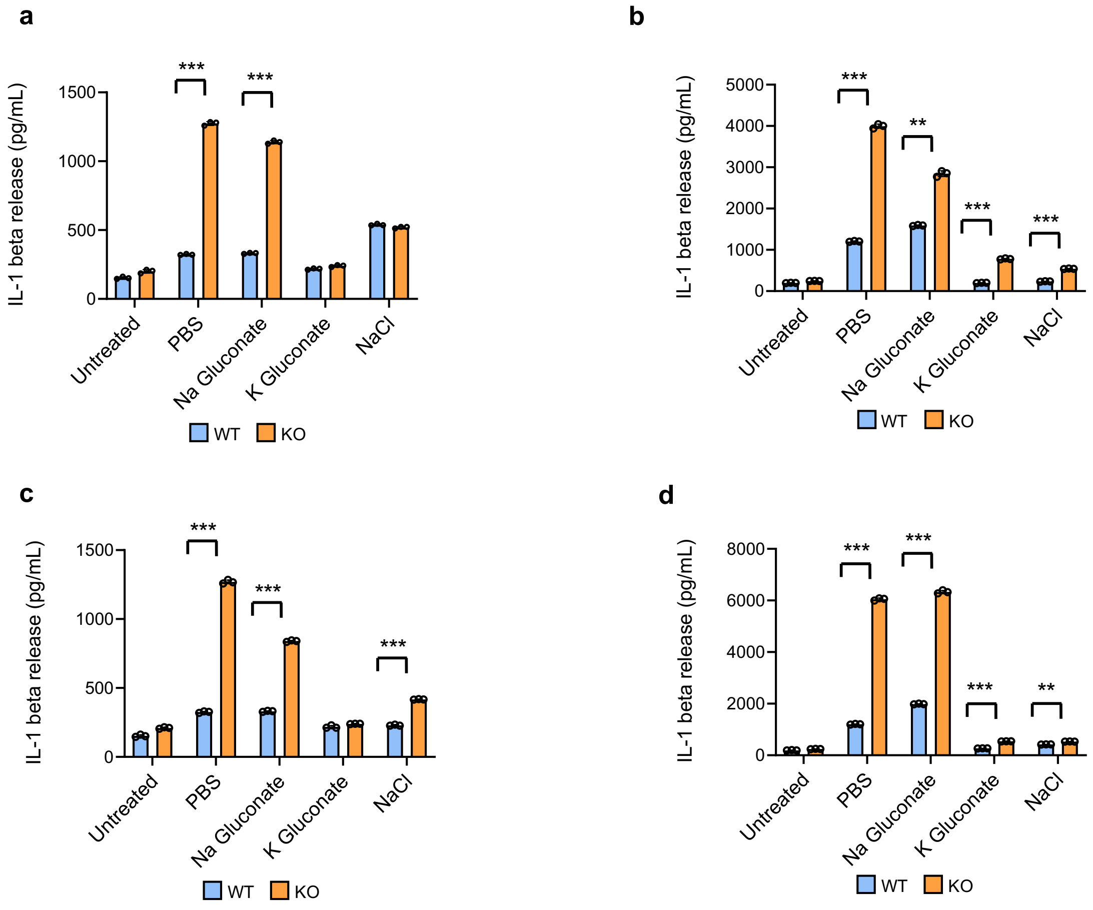

Supplementary Figure 19: Excess extracellular chloride or potassium, but not sodium, inhibits IL-1β generation in response to ATP or imiquimod. a-d IL-1β release of LPS-primed (4 hours) primary *Wnk1*$^{+/+}$ LysMCre^{+} (WT) and *Wnk1*$^{flox/flox}$ LysMCre^{+} (KO) BMDMs incubated in 65 mM (**a**, **b**) or 130 mM (**c**, d) NaGluconate, KGluconate or NaCl in PBS for one hour prior to stimulation with imiquimod (**a**, **c**) or ATP (**b**, **d**). P values in a are 0.0572, 0.0001, 0.0001, 0.0880, 0.1153; in b are 0.0547, 0.000175, 0.001215, 0.0003089, 0.0004205; in c are 0.00102, 0.000129, 0.0000050, 0.0506953, 0.0002467; in d are 0.0565, 0.000018, 0.000041, 0.000028, 0.000630. Results are representative of at least three independent experiments performed in duplicate or triplicate. Error bars in a-d are presented as mean values +/- standard deviation (S.D.); with n=3. Two-sided student's t-test, **$p < 0.005$, ***$p < 0.0005$.

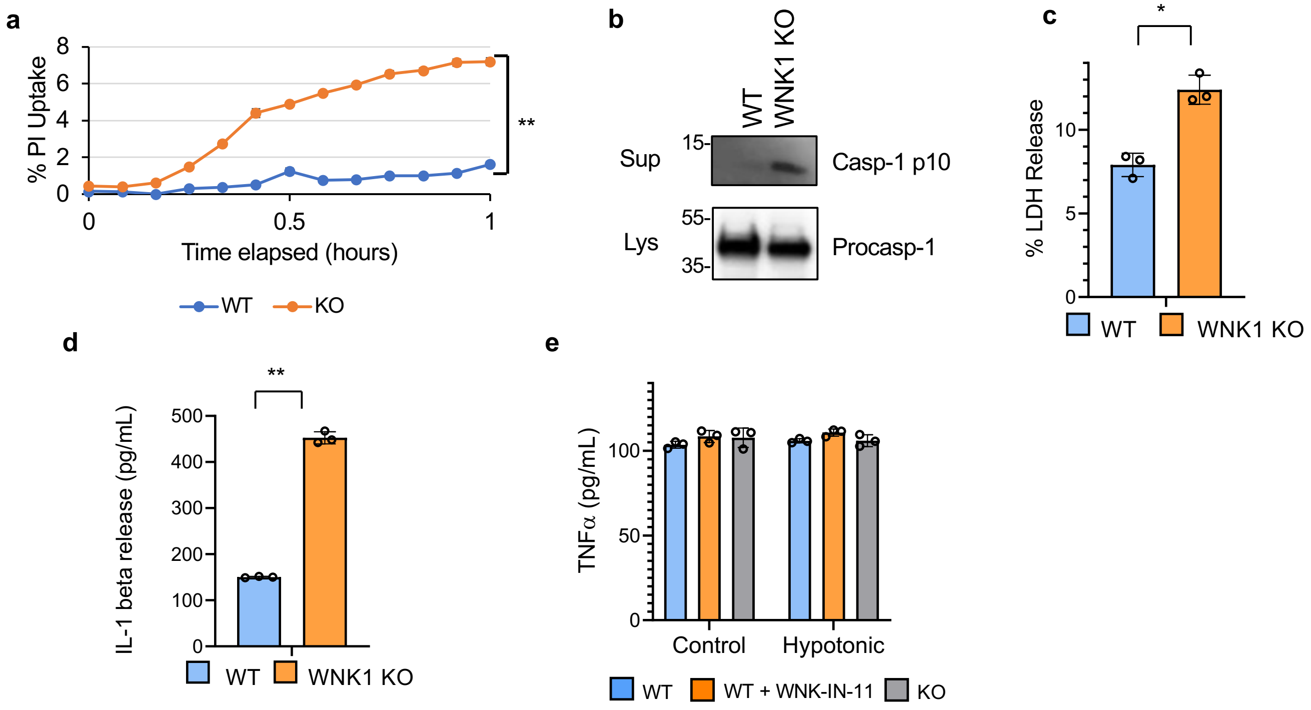

Supplementary Figure 20: Hypotonicity-induced NLRP3 activation is regulated by WNK1. **a** Propidium iodide uptake of LPS-primed (4 hours) primary *Wnk1*$^{+/+}$ LysMCre^{+} (WNK1 WT) and *Wnk1*$^{flox/flox}$ LysMCre^{+} (WNK1 KO) BMDMs incubated in a hypotonic solution with supplemented 5mM potassium gluconate for one hour. P value 0.0034. **b** Immunoblots of caspase-1 p20 released in culture supernatants (Sup) and procaspase-1 in cell lysates (Lys) of LPS-primed (4 hours) primary *Wnk1*$^{+/+}$ LysMCre^{+} (WNK1 WT) and *Wnk1*$^{flox/flox}$ LysMCre^{+} (WNK1 KO) BMDMs incubated in a hypotonic solution with supplemented 5mM potassium gluconate for one hour. **c** LDH release and **d** IL-1β release and **e** TNF-α release into cell culture supernatants of LPS-primed (4 hours) primary *Wnk1*$^{+/+}$ LysMCre^{+} (WNK1 WT) and *Wnk1*$^{flox/flox}$ LysMCre^{+} (WNK1 KO) BMDMs incubated in a hypotonic solution with supplemented 5mM potassium gluconate for one hour. P value for c is 0.01086 and d is 0.00062. Results are representative of at least three independent experiments performed in duplicate or triplicate. Error bars in a, c-e are presented as mean values +/- standard deviation (S.D.); with n=3. Two-sided student's t-test *p < 0.05, **p < 0.005.

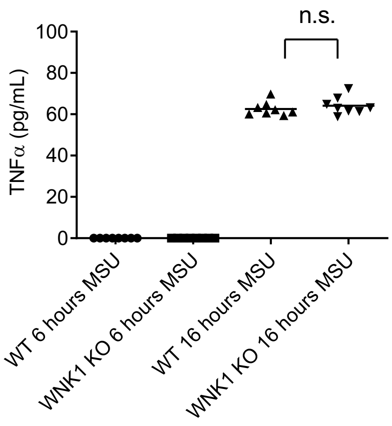

Supplementary Figure 21: TNFα is not impacted by WNK1 during MSU-induced peritonitis. TNF-α released from peritoneal exudate of *Wnk1*$^{+/+}$ LysMCre^{+} (WNK1 WT) and *Wnk1*$^{flox/flox}$ LysMCre^{+} (WNK1 KO) mice injected with 1 mg MSU for 6 hours or 16 hours; n=8 mice per group. Two-sided student's t-test results in this figure are not significant (n.s.); p value 0.3583.

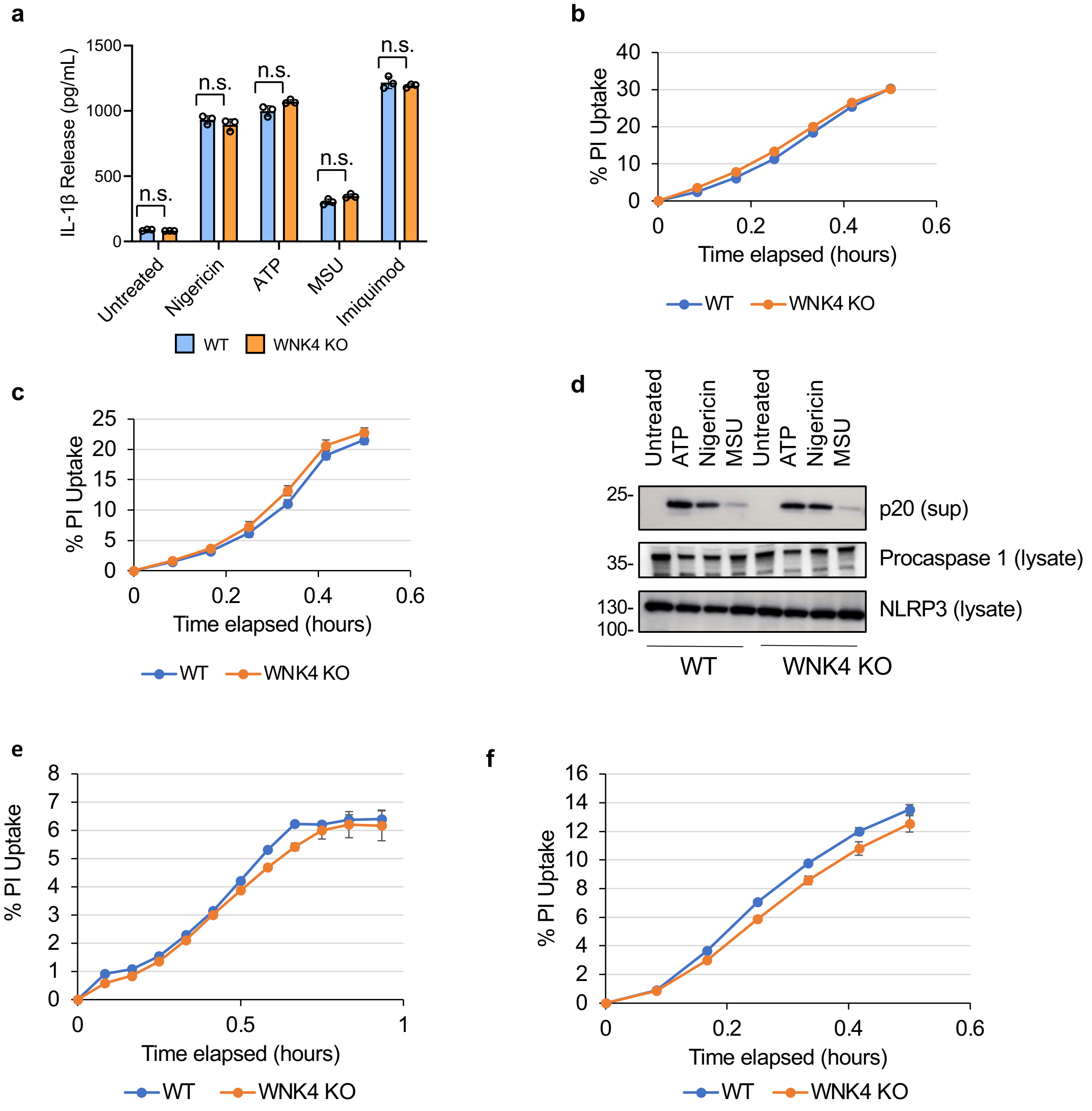

Supplementary Figure 22. WNK4 does not regulate NLRP3 activation. a IL-1β release in cell culture supernatants of LPS primed (4 hours) primary WNK4 wild type or WNK4 knockout BMDMs treated with nigericin, ATP, imiquimod, or MSU as indicated. P values left to right: 0.1839, 0.0551, 0.1507, 0.1263, 0.3891. **b, c**, **e, f** Propidium iodide uptake of LPS primed (4 hours) primary WNK4 wild type or WNK4 knockout BMDMs treated with Nigericin (**b**), ATP (**c**), imiquimod (**e**), or MSU (**f**). **d** Immunoblots of caspase-1 p20 released in culture supernatants (sup) and procaspase-1 and NLRP3 in cell lysates (lysate) of LPS primed (4 hours) primary WNK4 wild type or WNK4 knockout BMDMs treated with nigericin, ATP, or MSU as indicated. Results are representative of at least three independent experiments performed in duplicate or triplicate. Error bars in a-c, e-f are presented as mean values +/- standard deviation (S.D.); with n=3. Two-sided student's t-test results in this figure are not significant (n.s.).

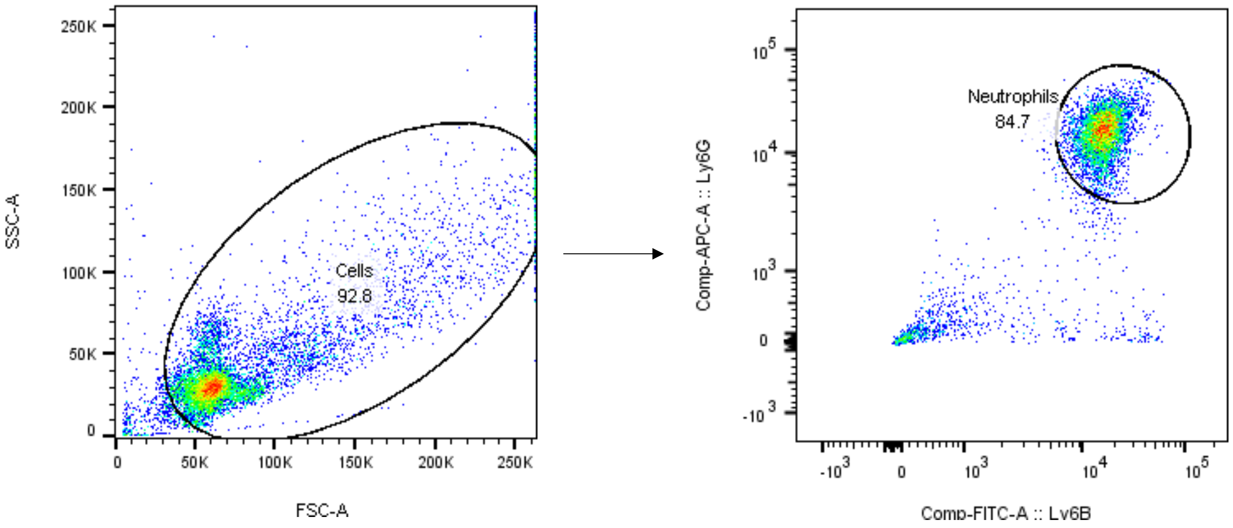

Supplementary Figure 23. Gating strategy to determine neutrophil population from peritoneal exudate. After excluding debris (left) cells that were $Ly6G^{+}$ and $Ly6B.2^{+}$ were considered neutrophils. This strategy was applied to the experiments displayed in Fig. 8 b-c, e-f and Fig. 9 d, e.

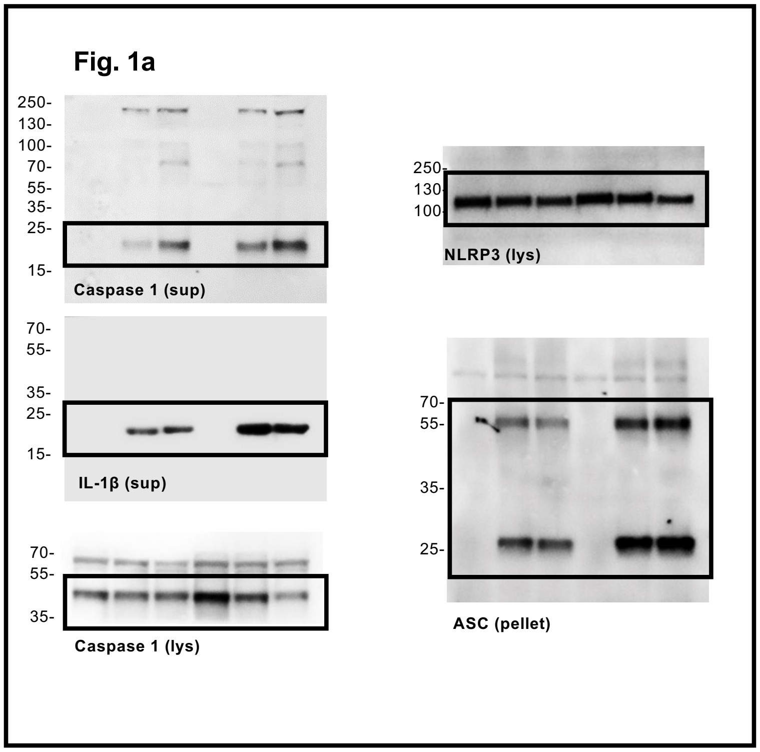

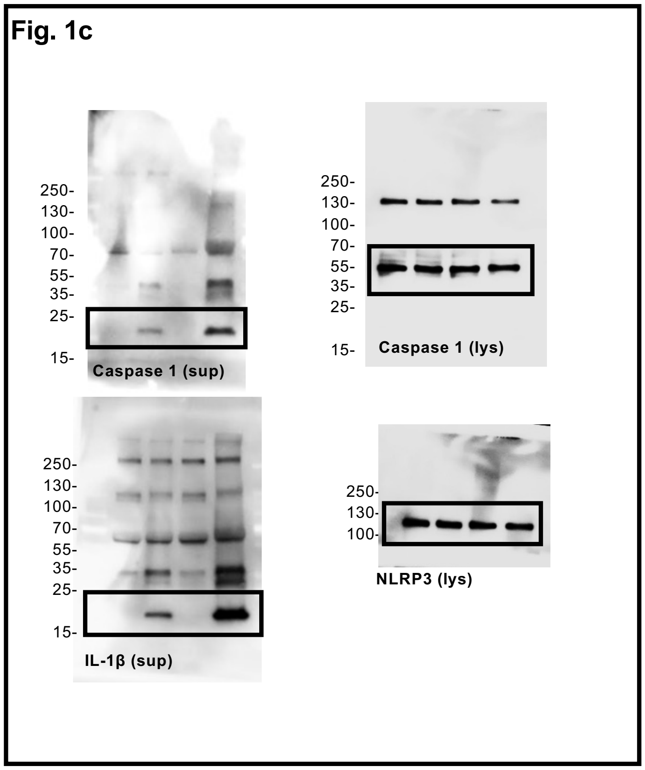

Supplementary Figure 24. Uncropped scans of western blots shown in Figure 1.

Fig. 2a

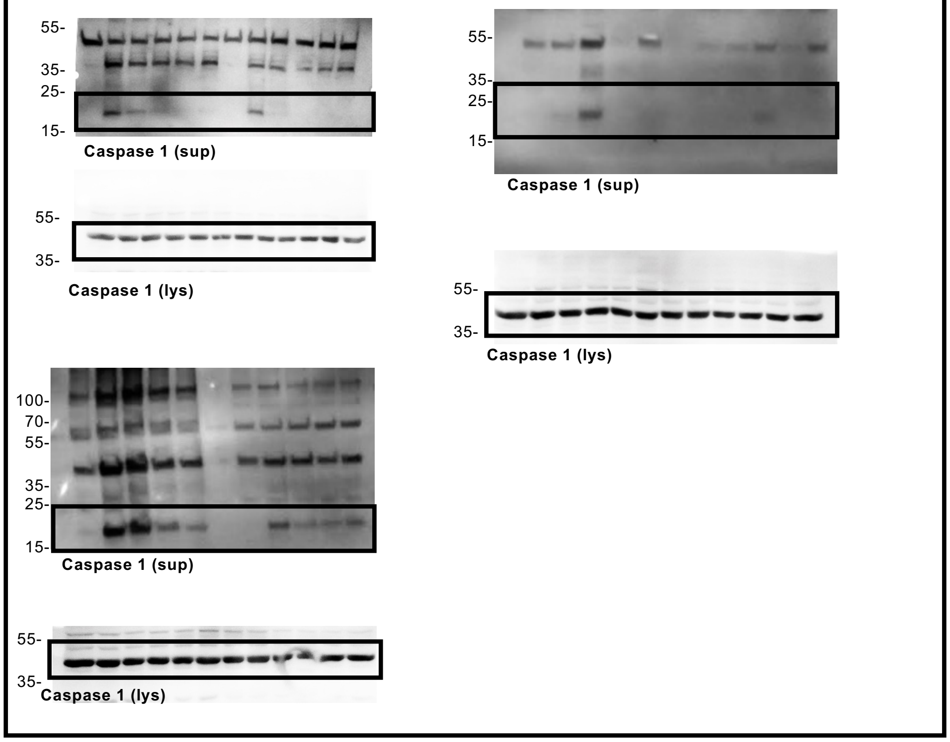

Fig. 2d

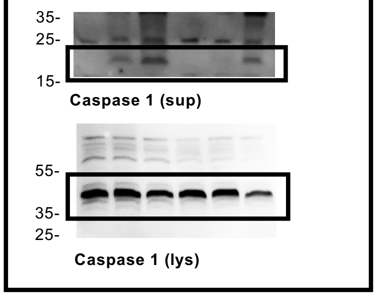

Supplementary Figure 25. Uncropped scans of western blots shown in Figure 2.

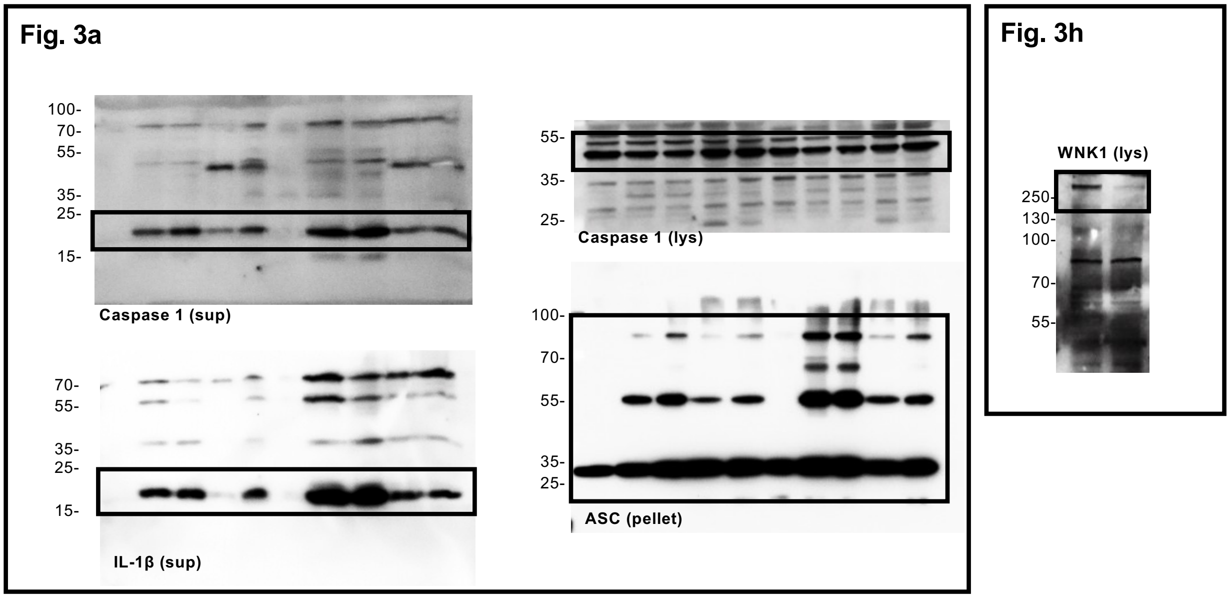

Supplementary Figure 26. Uncropped scans of western blots shown in Figure 3.

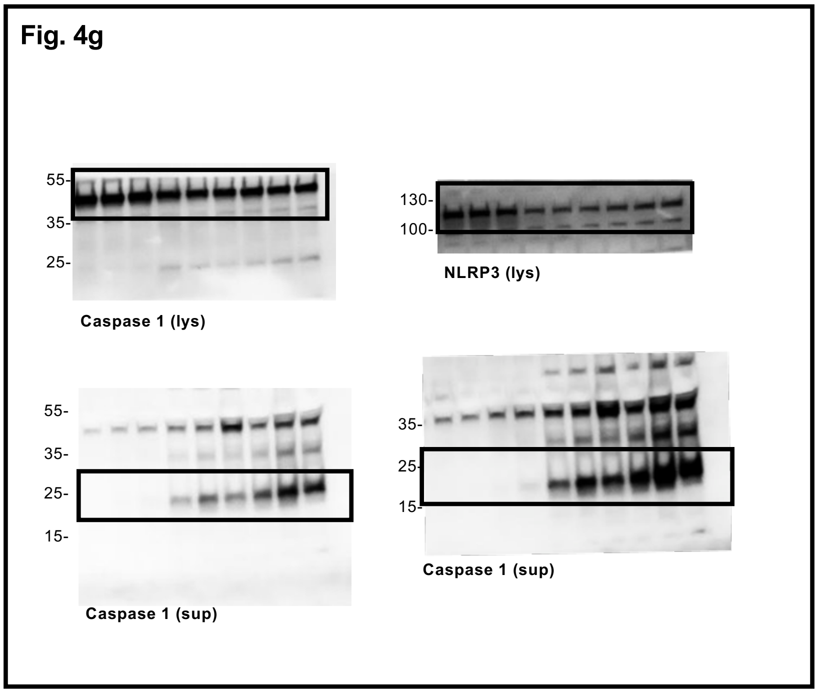

Supplementary Figure 27. Uncropped scans of western blots shown in Figure 4.

Fig. 5c

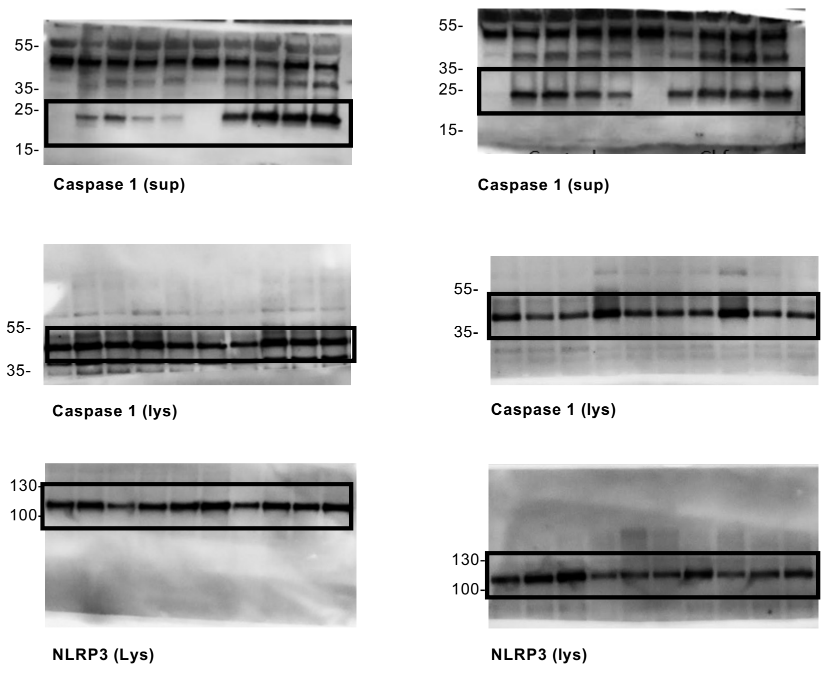

Supplementary Figure 28. Uncropped scans of western blots shown in Figure 5.

Fig. 9c

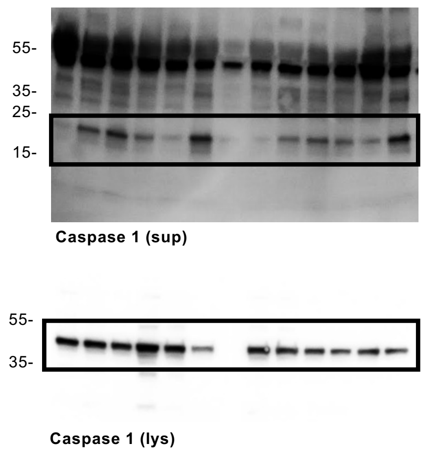

Supplementary Figure 29. Uncropped scans of western blots shown in Figure 9.

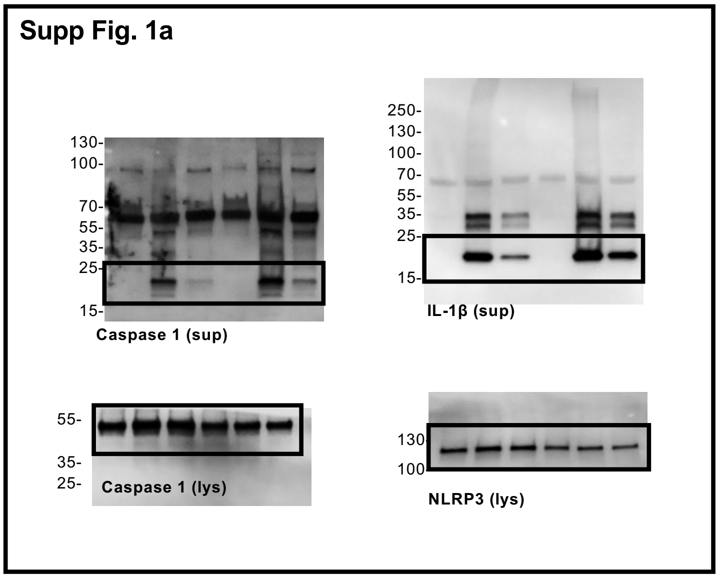

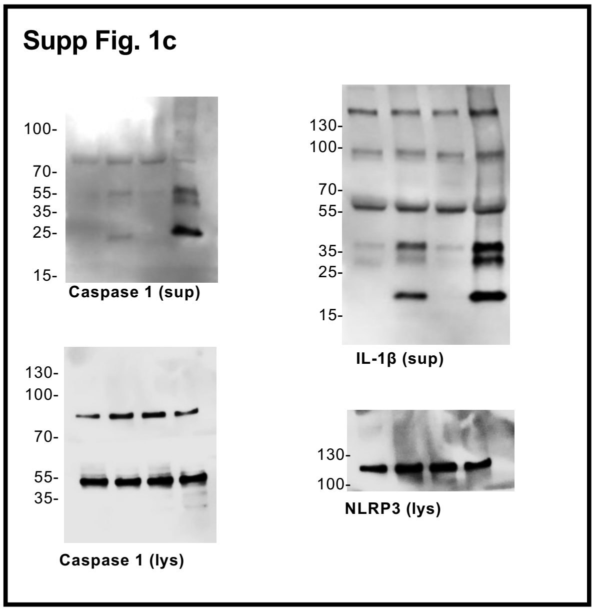

Supplementary Figure 30. Uncropped scans of western blots shown in Supplementary Figure 1.

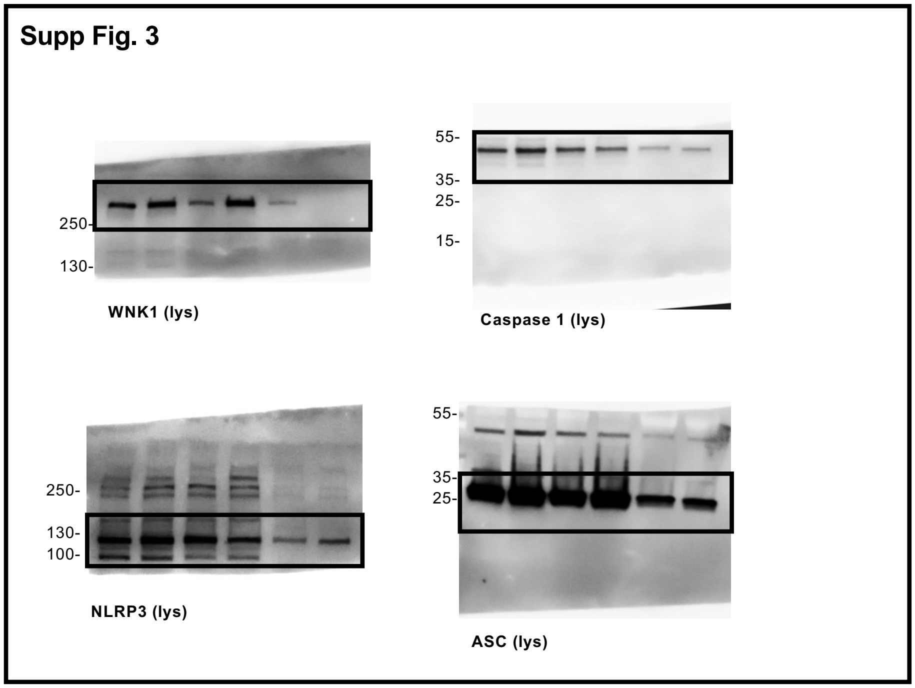

Supplementary Figure 31. Uncropped scans of western blots shown in Supplementary Figure 3.

Supp Fig. 4c

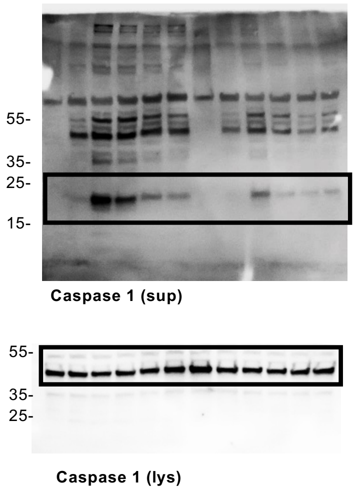

Supp Fig. 4d

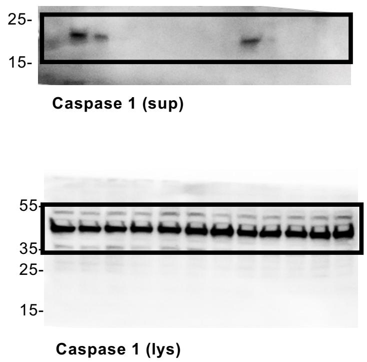

Supp Fig. 4f

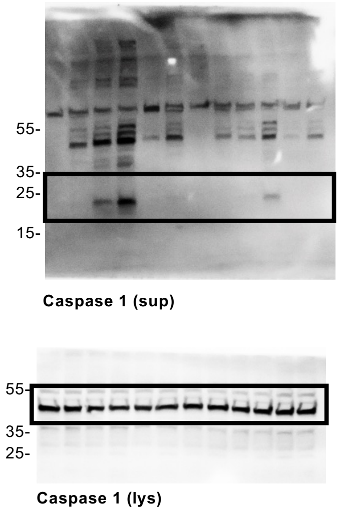

Supplementary Figure 32. Uncropped scans of western blots shown in Supplementary Figure 4.

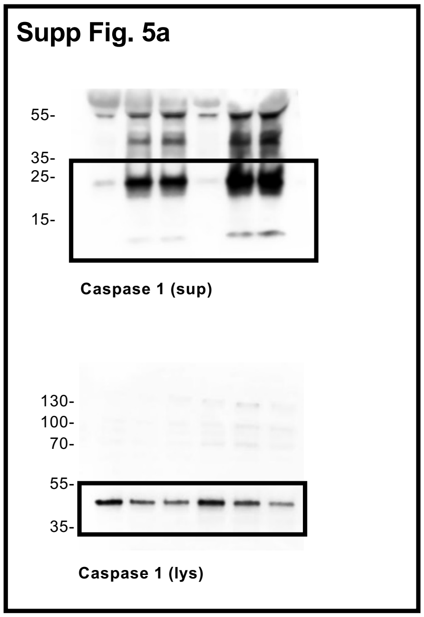

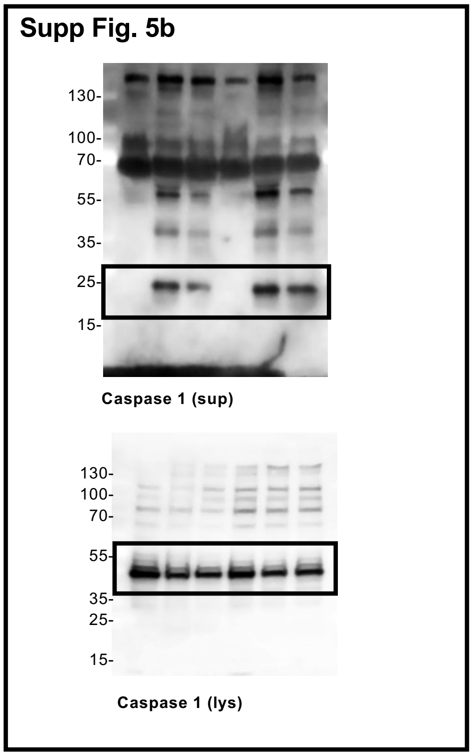

Supplementary Figure 33. Uncropped scans of western blots shown in Supplementary Figure 5.

Fig. 6d

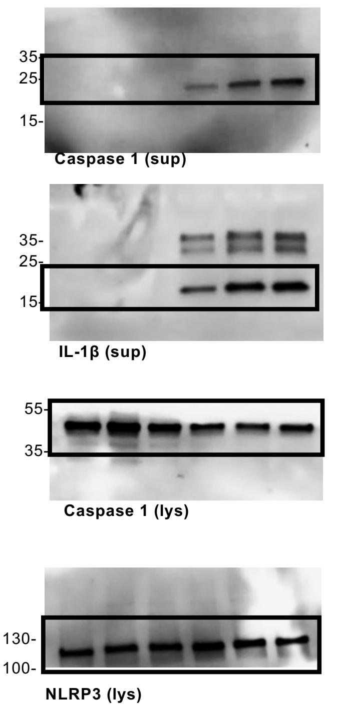

Fig. 6h

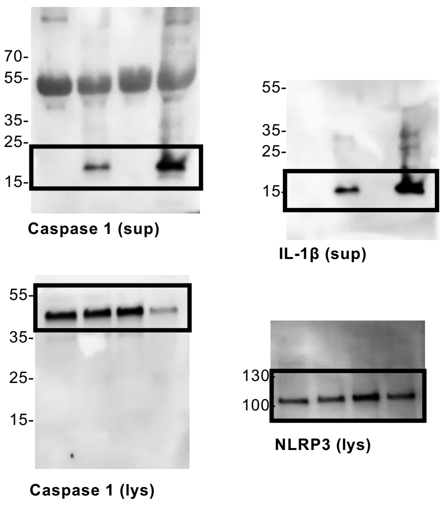

Supplementary Figure 34. Uncropped scans of western blots shown in Supplementary Figure 6.

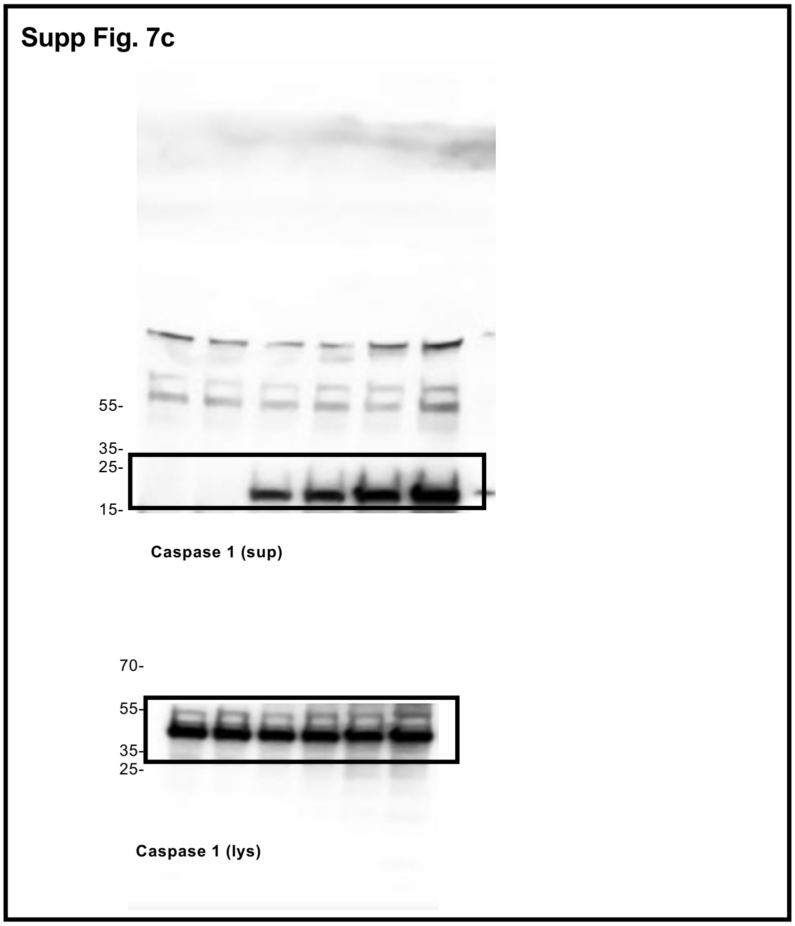

Supplementary Figure 35. Uncropped scans of western blots shown in Supplementary Figure 7.

Supp Fig. 8b

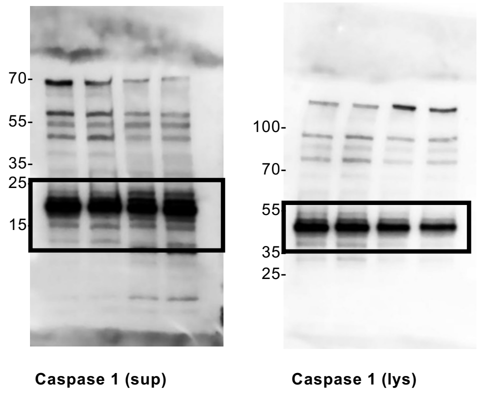

Supp Fig. 8e

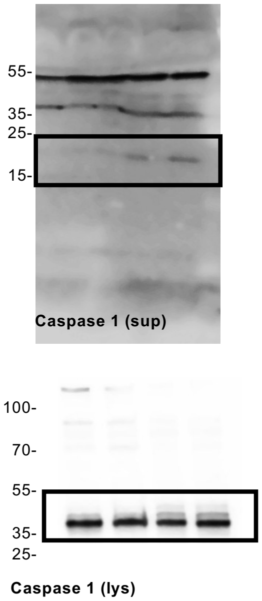

Supplementary Figure 36. Uncropped scans of western blots shown in Supplementary Figure 8.

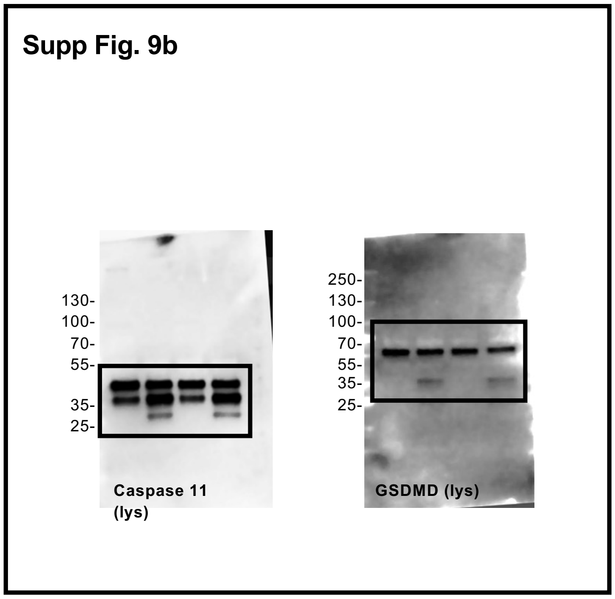

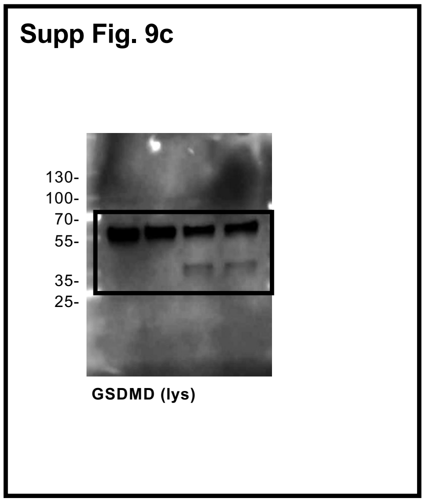

Supplementary Figure 37. Uncropped scans of western blots shown in Supplementary Figure 9.

Supp Fig. 10b

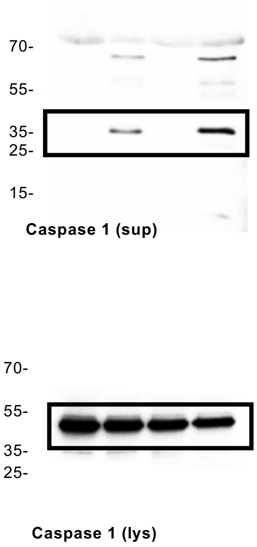

Supplementary Figure 38. Uncropped scans of western blots shown in Supplementary Figure 10.

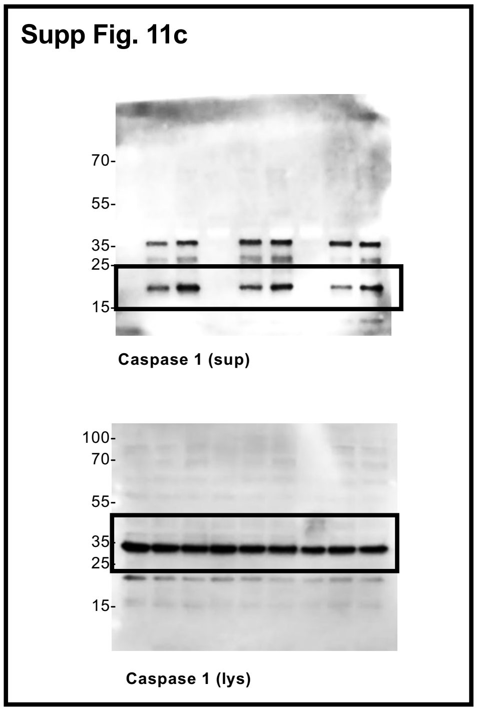

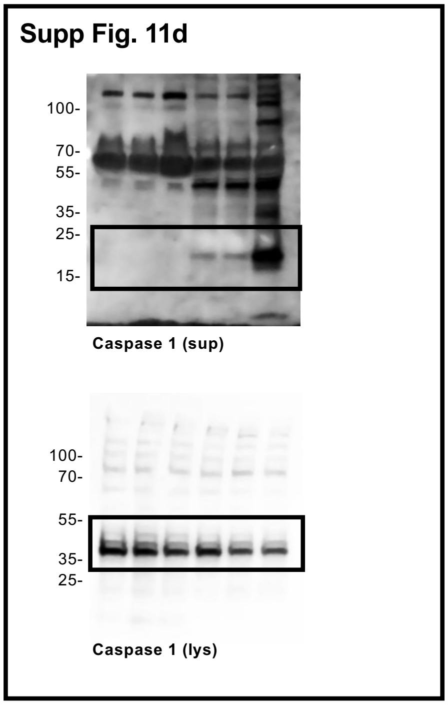

Supplementary Figure 39. Uncropped scans of western blots shown in Supplementary Figure 11.

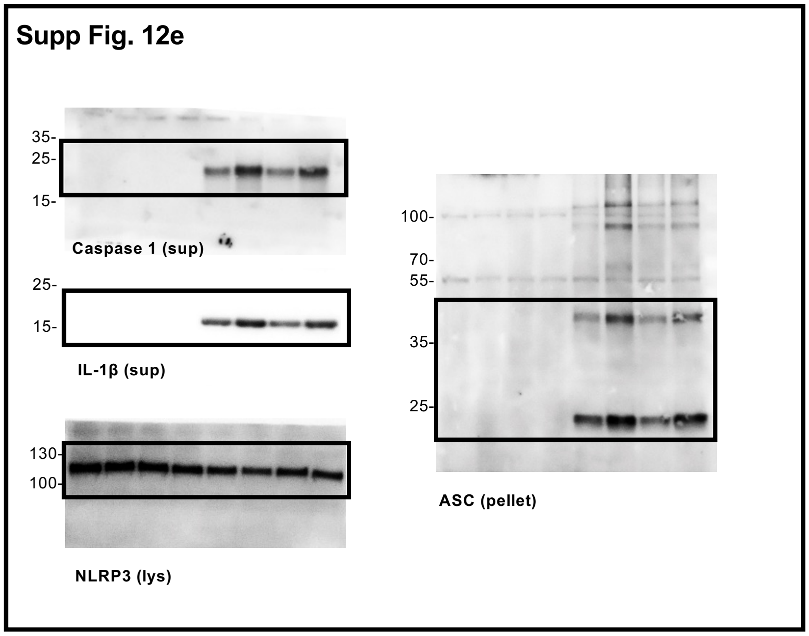

Supplementary Figure 40. Uncropped scans of western blots shown in Supplementary Figure 12.

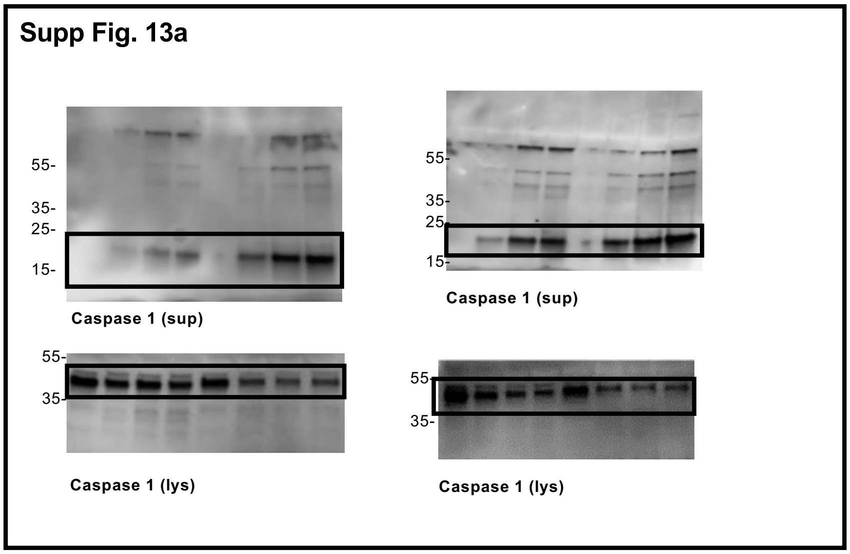

Supplementary Figure 41. Uncropped scans of western blots shown in Supplementary Figure 13.

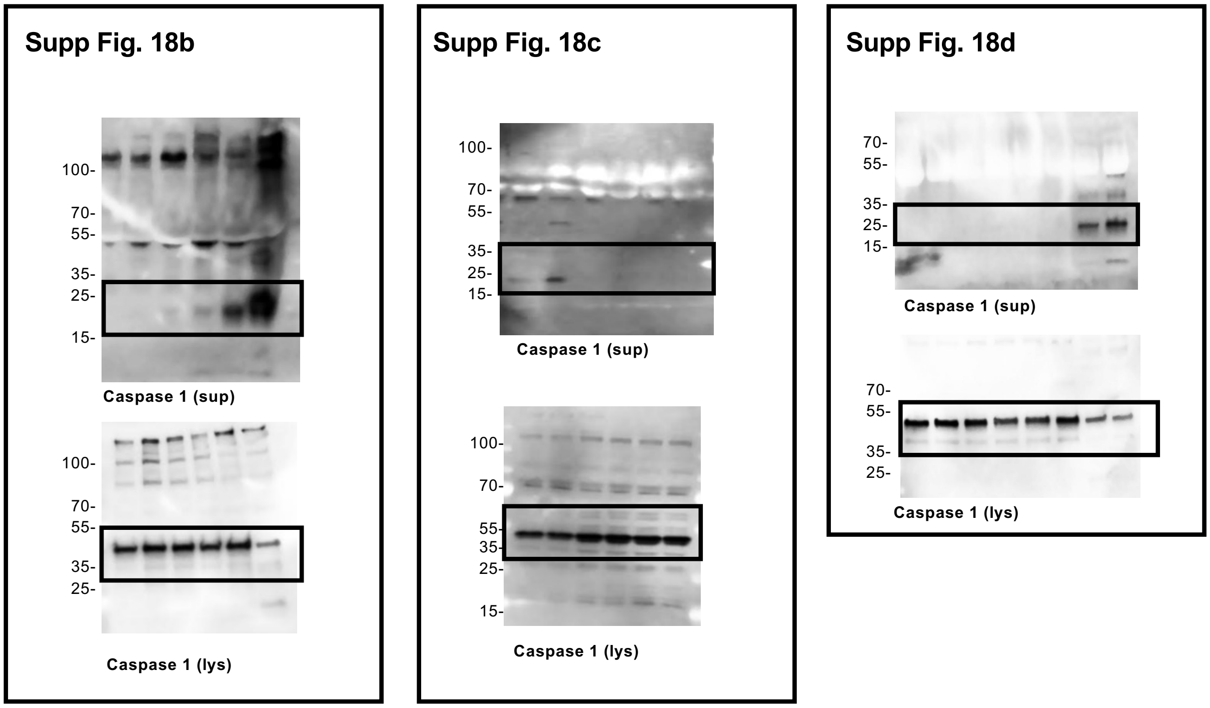

Supplementary Figure 42. Uncropped scans of western blots shown in Supplementary Figure 18.

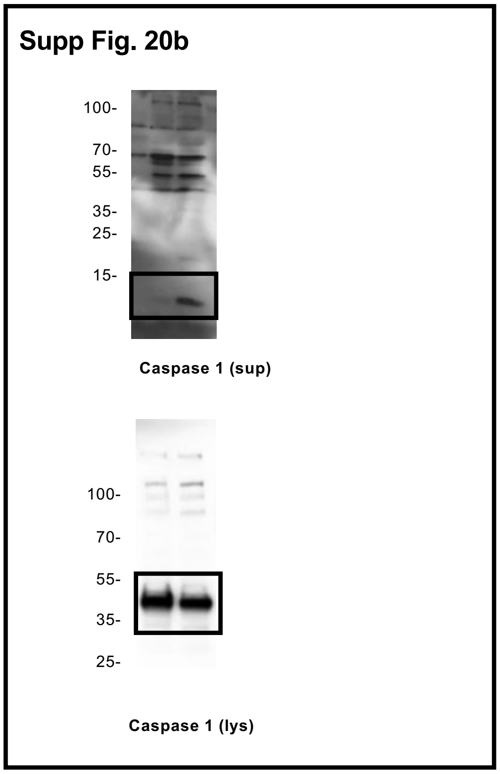

Supplementary Figure 43. Uncropped scans of western blots shown in Supplementary Figure 20.

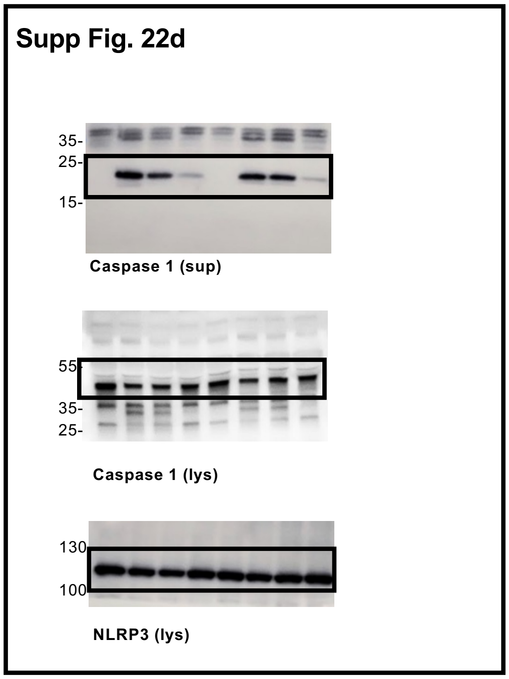

Supplementary Figure 44. Uncropped scans of western blots shown in Supplementary Figure 22.

Primer Name	Primer Sequence
Wnk1 exon 1 sg1	5’ CCGAGAAGCAAAGCGGCACTC 3’
Wnk1 exon 1 sg2	5’ TACAACGGCTTGTTCCCCAG 3’

Supplementary Table 1. Primers used in the study.
