# Supplementary material for: Chloride sensing by WNK1 regulates NLRP3 inflammasome activation and pyroptosis
Source: Nat Commun. 2021 Jul 27;12:4546. doi: 10.1038/s41467-021-24784-4 (PMC8316491; doi:10.1038/s41467-021-24784-4)
Supplement: Supplementary file 2 — Reporting Summary [file 41467_2021_24784_MOESM2_ESM.pdf]

nature portfolio

Corresponding author(s): Emad S. Alnemri

Last updated by author(s): Jun 14, 2021

Reporting Summary

Nature Portfolio wishes to improve the reproducibility of the work that we publish. This form provides structure for consistency and transparency in reporting. For further information on Nature Portfolio policies, see our Editorial Policies and the Editorial Policy Checklist.

Statistics

For all statistical analyses, confirm that the following items are present in the figure legend, table legend, main text, or Methods section.

n/a	Confirmed	
☐	☒	The exact sample size (n) for each experimental group/condition, given as a discrete number and unit of measurement
☐	☒	A statement on whether measurements were taken from distinct samples or whether the same sample was measured repeatedly
☐	☒	The statistical test(s) used AND whether they are one- or two-sided<br>*Only common tests should be described solely by name; describe more complex techniques in the Methods section.*
☐	☒	A description of all covariates tested
☐	☒	A description of any assumptions or corrections, such as tests of normality and adjustment for multiple comparisons
☐	☒	A full description of the statistical parameters including central tendency (e.g. means) or other basic estimates (e.g. regression coefficient) AND variation (e.g. standard deviation) or associated estimates of uncertainty (e.g. confidence intervals)
☐	☒	For null hypothesis testing, the test statistic (e.g. F, t, r) with confidence intervals, effect sizes, degrees of freedom and P value noted<br>*Give P values as exact values whenever suitable.*
☒	☐	For Bayesian analysis, information on the choice of priors and Markov chain Monte Carlo settings
☒	☐	For hierarchical and complex designs, identification of the appropriate level for tests and full reporting of outcomes
☒	☐	Estimates of effect sizes (e.g. Cohen's d, Pearson's r), indicating how they were calculated

Our web collection on statistics for biologists contains articles on many of the points above.

Software and code

Policy information about availability of computer code

Data collection	BD LSRFortessa was used for flow cytometry experiments.
Data analysis	FlowJo Version 10 was used to analyze flow cytometry experiments. Biorender.com was used to create the model in Fig. 10.

For manuscripts utilizing custom algorithms or software that are central to the research but not yet described in published literature, software must be made available to editors and reviewers. We strongly encourage code deposition in a community repository (e.g. GitHub). See the Nature Portfolio guidelines for submitting code & software for further information.

Data

Policy information about availability of data

All manuscripts must include a data availability statement. This statement should provide the following information, where applicable:
- Accession codes, unique identifiers, or web links for publicly available datasets
- A description of any restrictions on data availability
- For clinical datasets or third party data, please ensure that the statement adheres to our policy

The data that support the findings of this study are available within the article and supplementary files including a source data file, or are available from the corresponding author upon request.

Field-specific reporting

Please select the one below that is the best fit for your research. If you are not sure, read the appropriate sections before making your selection.

☒ Life sciences ☐ Behavioural & social sciences ☐ Ecological, evolutionary & environmental sciences

For a reference copy of the document with all sections, see nature.com/documents/nr-reporting-summary-flat.pdf

Life sciences study design

All studies must disclose on these points even when the disclosure is negative.

Sample size	A sample size of n= 8 was chosen for in vivo experiments based on previous published literature and protocols that performed similar experiments (Ex: Tang, T., Lang, X., Xu, C. et al. CLICs-dependent chloride efflux is an essential and proximal upstream event for NLRP3 inflammasome activation. Nat Commun 8, 202 (2017). https://doi.org/10.1038/s41467-017-00227-x). In support of our predetermined sample size, results/measurements were statistically different between groups. For all other experiments, a sample size of at least n=3 was chosen to provide sufficient results to generate significance values.
Data exclusions	No data were excluded.
Replication	All experimental findings were reproducible. To confirm this, individual experiments were performed either in duplicate or triplicate, and experiments were independently repeated at least three times.
Randomization	All samples were randomly assigned to experimental groups. Mice of equal age were alloted to experimental groups to ensure equal age factors.
Blinding	Investigators were blinded during collection and analysis of in vivo experiments. During this time, mice were assigned a number rather than being labeled with genotype and experimental treatment information. Blinding was not possible for all other experiments.

Reporting for specific materials, systems and methods

We require information from authors about some types of materials, experimental systems and methods used in many studies. Here, indicate whether each material, system or method listed is relevant to your study. If you are not sure if a list item applies to your research, read the appropriate section before selecting a response.

Materials & experimental systems

n/a	Involved in the study	
☐	☒	Antibodies
☐	☒	Eukaryotic cell lines
☒	☐	Palaeontology and archaeology
☐	☒	Animals and other organisms
☒	☐	Human research participants
☒	☐	Clinical data
☒	☐	Dual use research of concern

Methods

n/a	Involved in the study	
☒	☐	ChIP-seq
☐	☒	Flow cytometry
☒	☐	MRI-based neuroimaging

Antibodies

Antibodies used	Rabbit polyclonal antibodies against NLRP3 (immunogen: human amino acids 1-198) and caspase-1 (immunogen: mouse caspase 1 p20 subunit) were generated through Invitrogen. In brief, recombinant proteins were expressed in bacteria, affinity purified, and verified on Coomassie-stained PAGE gels. Titers >2048000 were obtained for both antibodies. NLRP3 antibody was used at 1:2000 dilution and Caspase-1 antibody was used at 1:1500 dilution. Anti-mouse ASC antibody was a gift from J. Sagara (Japan) and used at 1:2000 dilution. Anti-IL-1β was from GeneTex (Catalog No. GTX74034) and used at 1:10,000 dilution. Anti-WNK1 (2360 – 2382) [Sheep No. S062B] antibody was obtained from MRC PPU Reagents and was used at a 1:250 dilution. For in vivo experiments, Alexa Fluor 594 anti-mouse CD3ε (BioLegend Cat No. 152317), Ly-6B.2 FITC (Biorad Ref MCA771FT), Anti-mouse Ly6G APC (Tonbo Ref 20-1276-UO25), and Anti-mouse CD45 violetFluor 450 (Tonbo Ref 75-0451-UO25) were used at 1:200 dilution each.
Validation	NLRP3, ASC, and caspase-1 antibody specificities were verified by western blot analysis of protein lysates from immortalized wild type and knockout mouse macrophages validated by PCR genotyping, and HEK293T cells were transfected with full length human and mouse expression plasmids that had previously been verified by Sanger sequencing. IL-1beta- was validated by GeneTex for western blot; reactivity: human, mouse, rat; additional information is provided on GeneTex's website (catalog number mentioned above and in manuscript). WNK1-antibody information is provided on MRC PPU's website: https://mrcppureagents.dundee.ac.uk/reagents-view-antibodies/589520. Ly-6B.2- was validated for flow cytometry by BioRad; reactivity: mouse; additional information is provided on BioRad's website (catalog number MCA771FT). Ly6G was validated for flow cytometry by Tonbo; reactivity: mouse; additional information is provided on Tonbo's website (catalog number 20-1276-UO25) CD45 was validated by Tonbo for flow cytometry; reactivity: mouse; additional information is provided on Tonbo's website (catalog number 20-1276-UO25).

Eukaryotic cell lines

Policy information about cell lines

Cell line source(s)	Cell lines were derived from mouse bone marrow.
Authentication	Cell lines were validated by DNA sequencing and/or western blot to demonstrate knockout of applicable protein(s) when possible. Mice from which macrophages were derived were also genotyped prior to and following bone marrow collection to verify genotype.
Mycoplasma contamination	Cells were not tested for mycoplasma contamination.
Commonly misidentified lines (See ICLAC register)	None

Animals and other organisms

Policy information about studies involving animals; ARRIVE guidelines recommended for reporting animal research

Laboratory animals	Mice were all backcrossed (N6) on C57BL/6 background. Both females and males, aged 2-6 months, were used for generating bone marrow derived macrophages and for in vivo experiments. Mice were housed in pathogen-free conditions at 68-72 degrees Fahrenheit and 30-70% humidity on a 6pm/6am nocturnal dark/light cycle.
Wild animals	None
Field-collected samples	None
Ethics oversight	The authors complied with all relevant ethical regulations for animal testing and research. All experiments performed in this study received previous ethical approval and study protocols were approved by the Thomas Jefferson University Institutional Animal Care and Use Committee.

Note that full information on the approval of the study protocol must also be provided in the manuscript.

Flow Cytometry

Plots

Confirm that:

- [x] The axis labels state the marker and fluorochrome used (e.g. CD4-FITC).
- [x] The axis scales are clearly visible. Include numbers along axes only for bottom left plot of group (a 'group' is an analysis of identical markers).
- [x] All plots are contour plots with outliers or pseudocolor plots.
- [x] A numerical value for number of cells or percentage (with statistics) is provided.

Methodology

Sample preparation	1 million cells collected from the peritoneal lavage of mice treated with PBS or MSU for the indicated times were stained at 1:200 dilution of Ly-6B.2, Ly6G, and CD45 antibodies. After washing, samples were fixed with fixation buffer and stored at 4 degrees C until sorting.
Instrument	BD LSRFortessa 5 laser 18 color (355/405/488/561/640)
Software	Data was acquired on LSRFortessa (BD Biosciences) and analyzed using FlowJo software Version 10.
Cell population abundance	The abundance of relevant cell populations was determined by the presence or absence of cell markers corresponding to the antibodies used in the experiment.
Gating strategy	For measuring % infiltrating neutrophils, SSC-A and FSC-A gated cells were analyzed for neutrophil markers Ly6B and Ly6G. Neutrophils are highly positive for both markers (~10^4).

- [x] Tick this box to confirm that a figure exemplifying the gating strategy is provided in the Supplementary Information.
